# Total synthesis of a sorrentanone ester library and evaluation of its antimicrobial potential

**DOI:** 10.1038/s41598-025-05360-y

**Published:** 2025-07-04

**Authors:** Tobias M. Milzarek, Finn H. Gattwinkel, Sanja Š. Bogojević, Dušan Milivojević, Sandra Vojnović, Tobias A. M. Gulder, Jasmina Nikodinovic-Runic

**Affiliations:** 1 Technical University of Dresden, Bergstraße 66, 01069 Dresden, Germany; 2 Department of Natural Product Biotechnology, Helmholtz Institute for Pharmaceutical Research Saarland (HIPS), Helmholtz Centre for Infection Research (HZI) and Department of Pharmacy at Saarland University, PharmaScienceHub (PSH), 66123 Saarbrücken, Germany; 3 Institute of Molecular Genetics and Genetic Engineering, University of Belgrade, VojvodeStepe 444a, 11000 Belgrade, Serbia; 4 Institut Für Chemie, Technische Universität Berlin, Straße Des 17. Juni 115, 10623 Berlin, Germany

**Keywords:** Biocatalysis, Toxicology, Organic chemistry

## Abstract

Sorrentanone belongs to the class of monomeric sorbicillinoids and displays bioactivities against various Gram-positive bacteria including *Bacillus subtilis*, *Enterococcus faecalis*, and *Streptococcus* as well as *Staphylococcus* species. Within this work, we present the optimization of the synthetic access towards the sorrentanone scaffold, facilitating the formation of a focused library of structural analogs with ester side chains. Exploration of the bioactivities of this compound library displayed the improvement of antibacterial properties and the presence of additional antifungal activities. Initial mode of action analyses showed the influence of the compounds on fungal filament propagation and biofilm production. Overall, this work reveals the pharmaceutical relevance of less studied monomeric sorbicillinoids.

## Introduction

**Fig. 1:**
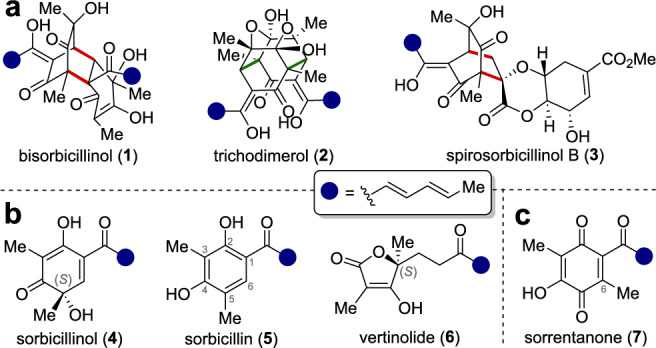
Overview of different sorbicillinoid natural products. (**a**) Dimeric and hybrid sorbicillinoids formed by [4 + 2] cycloaddition (red bonds) or Michael addition (green bonds) reactions. (**b**) Selection of monomeric sorbicillinoids. (**c**) Quinone-type related sorrentanone (**7**).^1,2^

Within the plethora of natural products, the structural diversity as well as numerous biological activities render the class of sorbicillinoids particularly interesting for pharmaceutical research.^1,2^ The best-known representatives include the DPPH radical scavenger bisorbicillinol (**1**) and the cytotoxic TNF-α inhibitor trichodimerol (**2**), which both belong to the subclass of dimeric sorbicillinoids (Fig. 1a).^3–5^ Spirosorbicillinol B (**3**) is a hybrid sorbicillinoid and is formed by a Diels–Alder reaction (red bonds) between sorbicillinol (**4**) and scytolide.^6,7^ Bioretrosynthetically, most dimeric and hybrid sorbicillinoids are deduced from the monomeric building block sorbicillin (**5**), which is transformed into the reactive key intermediate sorbicillinol (**4**) using the flavin-dependent monooxygenase SorbC (Fig. 1b).^8,9^ In addition to dimerization reactions, **4** can also undergo rearrangement reactions, leading to considerable structural diversity also within the class of monomeric sorbicillinoids, with vertinolide (**6**) being one example.^10^.

**Fig. 2:**
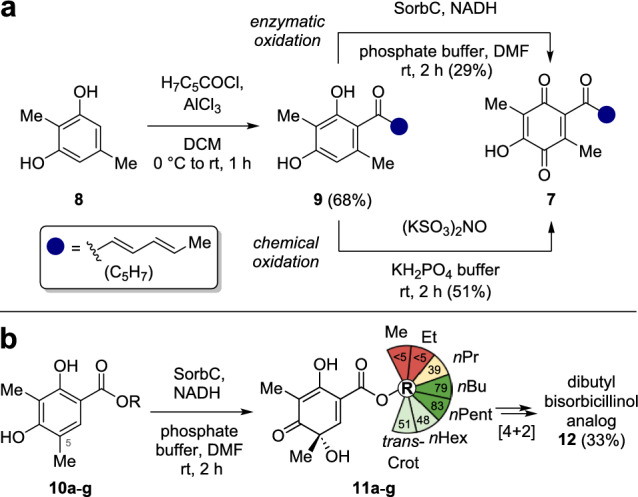
Synthetic routes for the formation of sorrentanone and substrate-mimicking strategy. (**a**) Enzymatic oxidation using SorbC *versus* chemical oxidation with Frémy’s salt.^11,12^ (**b**) Ester mimicking strategy.^13^.

Compared to their dimeric and hybrid counterparts, monomeric sorbicillinoids are underinvestigated, despite their significant bioactivities and easier synthetic access. An outstanding representative of this class is the quinone type natural product sorrentanone (**7**). It has a wide range of antibacterial activities, for example against *Bacillus subtilis* (IC_50_ = 32.0 µg/mL), *Enterococcus faecalis* (IC_50_ = 128 µg/mL), and various *Streptococcus* and *Staphylococcus* species (IC_50_ = 16.0–64.0 µg/mL).^14,15^ Bioretrosynthetically, sorrentanone (**7**) most likely stems from a SorbC-catalyzed oxidative dearomatization of sorbicillin analog **9** (Fig. 1c). In the past, our group established two total syntheses of **7** starting from resorcinol **8** (Fig. 2a). Friedel–Crafts acylation of **8** with sorbyl chloride provides **9** in up to 68% yield.^11^ The subsequent biocatalytic oxidative dearomatization to quinone **7** with SorbC yields 29%, whereas the chemical method utilizing Frémy's salt yields 51%.^11,12^ The combination of antibiotic activity and straightforward synthetic access provides the ideal basis for the generation of a sorrentanone analog library facilitating the exploration of its antimicrobial potential. Furthermore, our previously developed ester mimicking strategy using esters **10a**-**g** as substrates can be used as a template for possible derivatizations (Fig. 2b).^13,16^ Depending on the side chain length, esters **10a**-**g** can be converted enzymatically into corresponding sorbicillinols **11a**-**g** allowing the formation of dimers, such as **12**, bearing a flexible functionality. Building on the above mentioned insights, we thus set out to synthesize an ester-type sorrentanone library to screen its activity against several pathogenic bacterial and fungal strains.

## Results and discussion

### Synthesis of sorrentanones

**Fig. 3:**
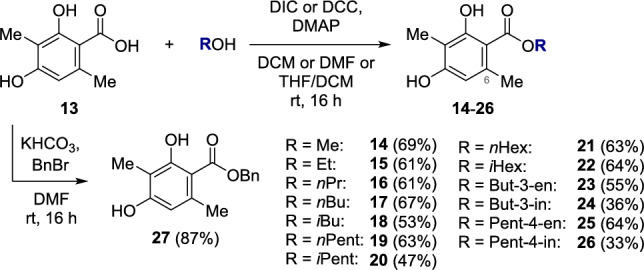
Formation of esters **14**–**27**. Steglich-type esterification (**14**–**26**) and carboxylic acid benzyl protection (**27**).

The sorrentanone analogs are derived of 2,4-dihydroxy-3,6-dimethylbenzoic acid (**13**), which is either used in a Steglich-type esterification or a benzyl protection (Fig. 3). Esterification with different alcohols enabled the formation of 6-methyl sorbicillin esters with a wide range of functionalities, including linear carbon chains (Me: **14**, 69%; Et: **15**, 61%; *n*Pr: **16**, 61%; *n*Bu: **17**, 67%; *n*Pent: **19**, 63%; *n*Hex: **21**, 63%), branched analogs (*i*Bu: **18**, 53%; *i*Pent: **20**, 47%; *i*Hex: **22**, 64%), double (but-3-enyl: **23**, 55%; pent-4-enyl: **25**, 64%), and triple bonds (but-3-inyl: **24**, 36%; pent-4-inyl: **26**, 33%).^13^ The aromatic benzyl ester **27** was prepared by an S_N_2 reaction between benzoic acid **13** and benzyl bromide (87% yield).

Based on the previously reported synthesis of sorrentanone (**7**) using Frémy’s salt ((KSO_3_)_2_NO), we investigated the oxidation of model butyl ester **17** under the same conditions (Table 1, entry 1).^12^ To our surprise, no corresponding quinone oxidation product could be isolated. Adaptation of the chemical oxidation using the hypervalent iodine reagent (bis(trifluoroacetoxy)iodo) benzene (PIFA) allowed the formation of the desired butylquinone **31** in 10% yield (entry 2).^17^ Further attempts to optimize the reaction by increasing the temperature to 100 °C or by employing stoichiometric amounts of oxidant resulted in product decomposition. The application of other oxidants (e.g. IBX, salcomine) showed no improvement in quinone formation (see Supplementary, Table S1). In addition to chemical oxidation, the enzymatic method using the monooxygenase SorbC was also explored (entry 3).^9^ Corresponding enzymatic assay including HPLC analysis showed formation of sorrentanone butyl ester with 82% conversion.

**Table 1:** Optimization of the chemical oxidation.


	Oxidizing agent(s)	Solvent(s)	Temp	Time	Yield^a^
1^12^	Frémy’s salt (3.6 equiv.)	acetone/H_2_O = 1:1	25 °C	2 h	no reaction
**2**^13^	PIFA (1.2 equiv.)	MeCN/H_2_O = 10:1	25 °C	1 h	10%^b^
**3**	SorbC (0.5 mol%)	phosphate buffer (pH 8.0), acetone	25 °C	1.5 h	82%^c^

^a^Screening reactions depicted in the table were performed on 0.20 mmol scale using butyl ester **17**. Isolated yield.

^b^Higher temperatures (100 °C and stochiometric amounts (2.4 equiv.) led to product decomposition.

^c^Conversion was analyzed by HPLC using corresponding substrate calibration curves. Further approaches can be found in the Supplementary Material (Table S1).

**Fig. 4:**
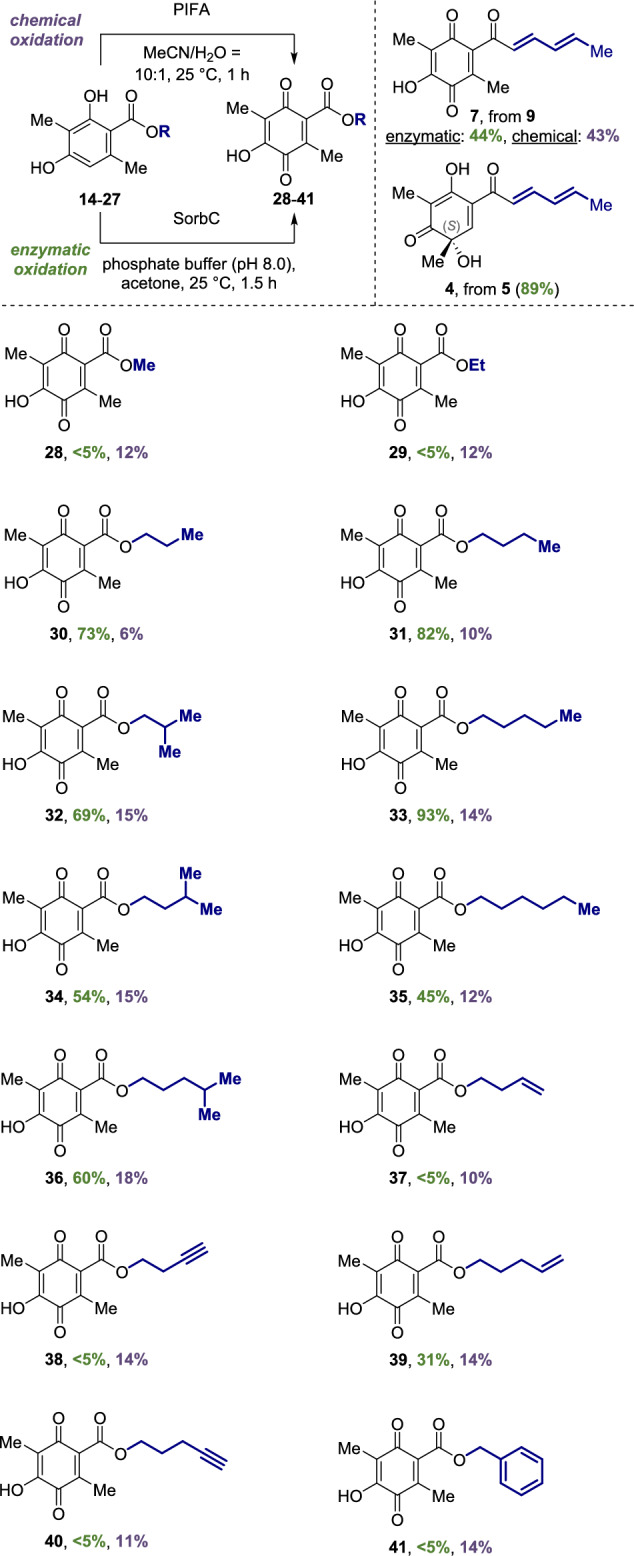
Scope of sorrentanone analogs **28**–**41**. Comparison of the chemical yield (purple) with the enzymatic conversion (green) using the monooxygenase SorbC. Reactions were performed on 0.25–0.40 mmol scale.

With two oxidation conditions in hands (Fig. 4, top left), we studied the scope using the different 6-methyl sorbicillin esters **14**–**27**. The synthesis of sorrentanone (**7**) from **9** and the SorbC-catalyzed oxidation of the natural substrate sorbicillin (**5**) to sorbicillinol (**4**) served as references (Fig. 4, top right). Sorrentanone (**7**) can be obtained by PIFA-mediated oxidation in 43% yield or with an enzymatic conversion of 44%, whereas sorbicillinol (**4**) can be produced in 89% yield. First, saturated linear and branched carbohydrate chains were investigated (Fig. 4, bottom). Methyl (**28**) and ethyl quinone (**29**) were obtained in 12% yield each by chemical oxidation, while no enzymatic conversion was observed (< 5%). Propyl (**30**) and *n*-butyl analog **31** were obtained in slightly lower yields using PIFA (**30**: 6%, **31**: 10%). In contrast, the enzymatic reaction showed a turnover similar to sorbicillin (**5**, 89%, **30**: 73%, **31**: 82%). This trend continued for branched *iso*-butyl derivative **32** (chemical: 15%, enzymatic 69%), pentyl derivatives **33** (chemical: 14%, enzymatic: 93%) and **34** (chemical: 15%, enzymatic: 54%) as well as for hexyl analogues **35** (chemical: 12%, enzymatic: 45%) and **36** (chemical: 18%, enzymatic: 60%). The unsaturated derivatives **37**–**40** were synthesized in average chemical yields (**37**: 10%, **38**: 14%, **29**: 14%, **40**: 11%). However, the enzymatic turnover was generally lower (**37**: < 5%, **38**: < 5%, **40**: < 5%), with the exception of **39** (31%). Aromatic ester **28** was obtained from the PIFA oxidation to give benzyl quinone **41** in 14% yield. Submission to the enzymatic assay revealed a lack of tolerance of SorbC (< 5%) towards this substrate.

In general, sorrentanone analogues **28**–**41** were obtained in lower yields compared to natural product **7**, with a maximum of 18% yield in chemical oxidation. The enzymatic conversion is best for chains with three to six carbon atoms (**30**–**36**, 45–93% yield), supporting previous substrate tolerance studies of SorbC.^13,16^ Carbon chains that are too short (**28**, **29**), unsaturated functionalities (**37**–**40**), and bulky aromatic systems (**41**) are less readily accepted by SorbC. Thus, chemical oxidation with PIFA and biocatalytic conversion with SorbC complement each other almost perfectly. In total a library of 14 sorrentanone analogs was synthesized and submitted to bioactivity evaluation.

### Biological activity evaluation

The synthesized sorrentanone library and the natural product **7** were evaluated for their antimicrobial activity against *Staphylococcus*, *Bacillus*, *Escherichia*, and *Pseudomonas* sp. and fungal *Candida* sp. (Table 2). Based on the reported antibacterial activity of **7**, we started with the analysis of the bioactivity of the library against Gram-positive bacteria.^14,15^ The complete library showed antibacterial activity against two *S. aureus* strains and *B. subtilis* ATCC6633. Compared to sorrentanone (**7**), quinone derivatives **33** (*n*Pent), **34** (*i*Pent), **36** (*i*Hex), **39** (pent-4-enyl), and **41** (benzyl), demonstrated a two- to four-fold improved activity. No bioactivity was observed against Gram-negative bacteria, such as *E. coli* or *P. aeruginosa*. In addition to the antibacterial properties, the antifungal activity of sorrentanone (**7**) and its derivatives was also investigated for the first time in this study. To our delight, quinone esters **33** (*n*Pent), **34** (*i*Pent), **35** (*n*Hex), **36** (*i*Hex), **39** (pent-4-enyl), **40** (pent-4-inyl), and **41** (benzyl), again, exhibited two- to four-fold improved activity against *Candida albicans* species in comparison to natural product **7,** while the activity against other *Candida* species such as *C. parapsilosis*, *C. krusei*, and *C. glabrata* was minimal or completely absent (data not shown). To further support these promising results and to assess selectivity, the cytotoxicity of the most active derivatives was analyzed.

**Table 2:**
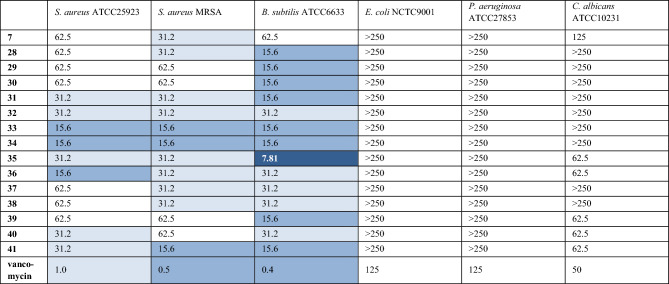
Antimicrobial activity screening of sorrentanone (**7**) and synthesized analogs **28**–**41**.

Minimum inhibitory concentration (MIC) against different *Staphylococcus*, *Bacillus*, *Escheria*, *Pseudomonas, and Candida albicans* species are given in µg/mL. Blue color indicates antimicrobial activity (darker blue equals higher activity). ATCC = American Type Culture Collection, NCTC = National Collection of Type Cultures (Culture Collection of Public Health, Salisbury UK).

### Analysis of cytotoxicity

**Fig. 5:**
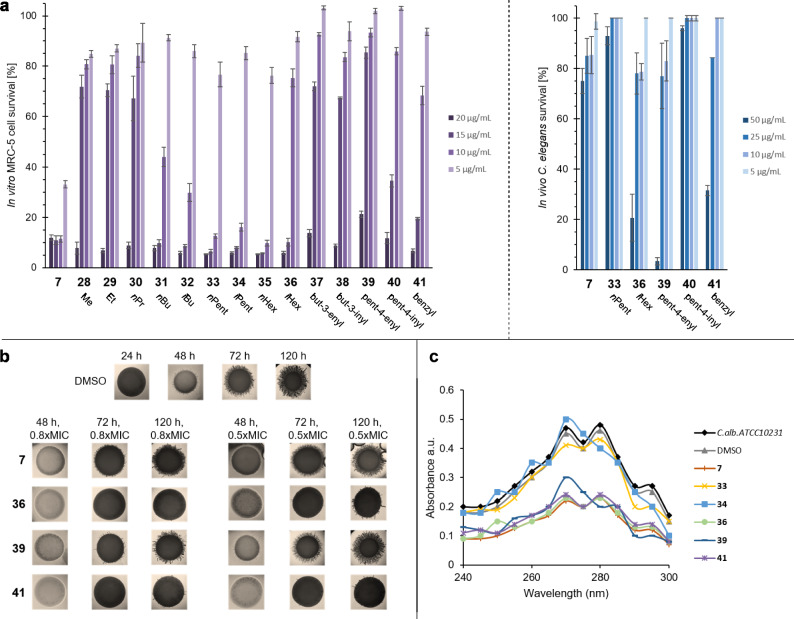
Overview of cytotoxicities, influence on *Candida* filamentation, and mode of action analysis. (**a**) Quantification of in vitro MRC-5 (left) and in vivo* C. elegancs* cytotoxicity (right). (**b**) Filamentation of *C. albicans* ATCC 10231 in the presence of sorrentanone (**7**) or derivatives **36**, **39**, **41** on solid spider medium. (**c**) Mode of action determination by analysis of ergosterol (component of fungal cell membranes) UV profiles in *C. albicans*.

Selected compounds were evaluated for their in vitro cytotoxic properties against healthy human fibroblasts (MRC-5, Fig. 5a, left), as well as in vivo toxicity in the *Caenorhabditis elegans* model (Fig. 5a, right). In MRC-5 cells, all compounds induced 85% or higher cell death at a concentration of 20 µg/mL. The lowest in vitro toxicity was observed for quinone esters **28**–**30** (Me, Et, *n*Pr) and **37**–**39** (but-3-enyl, but-3-inyl, pent-4-enyl), showing over 60% cell survival at a concentration of 15 µg/mL. Derivatives **36** (*i*Hex), **40** (pent-4-inyl), and **41** (benzyl) displayed the same behavior from a concentration of 10 µg/mL. At the lowest concentration of 5 µg/mL, all tested compounds eventually achieved complete cell survival. Interestingly, the natural sorrentanone (**7**) shows the highest toxicity in this study, followed by the ester quinones **31**–**35**. The toxicity can therefore be fine-tuned by the length of the ester side chains and the incorporation of unsaturated functionalities. Longer carbon chains (**31**–**36**, butyl to hexyl) lead to more toxic analogs than short chains (**28**: Me, **29**: Et, **30**: *n*Pr), while the incorporation of double (**37**, **39**) and triple bonds (**38**, **40**) or aromatic moieties (**41**) generally reduces cell toxicity.

The *C. elegancs* model showed a comparatively lower in vivo toxicity of compounds **33**, **36** and **39**–**41** (Fig. 5a, right). The *n*-hexyl and pent-4-inyl quinone esters **36** and **41** displayed the lowest toxicity with survival rates of over 90% at the highest tested concentration of 50 µg/mL. Again, all analyzed compounds attained complete cell survival at a concentration of 5 µg/mL with the exception of sorrentanone (**7**).

Sorrentanone (**7**) and the esters shown are less active than vancomycin in terms of antibiotic potential (Table 2). This is also reflected in the selectivity indices (SIs): 200 for vancomycin, 1.12 for sorrentanone (**7**), and 0.77 for the benzyl ester **41**. Additionally, the SIs indicate that the antimicrobial activity of sorrentanone and its derivatives is related to general toxicity rather than a selective antibacterial effect. A similar effect has already been observed in case of the sorbicatechols, which were described as being antiviral and for which we could show that activity was rahter due to cytotoxicity to the viral host cells.^18,19^.

### Influence on fungal filamentation and biofilm activity

In medicinal chemistry, quinones are known antifungal compounds against *C. albicans* by inhibition of cell growth and morphological transition.^20^ Despite the rather weak antifungal properties of our sorrentanone library and the fact that one of the main pathogenic properties of *Candida* species is the morphological transformation from yeast to hyphal form, we set out to analyze the influence of the synthesized compounds on the hyphal formation in *C. albicans* ATCC10231 (Fig. 5b). Without the addition of sorrentanone (**7**) or corresponding esters (**36**, **39**, **41**), filamentation is observed after 48 h, which becomes very pronounced after 120 h (see DMSO control). When using 0.8 times the MIC of the tested compounds, an inhibition of filament formation at 72 h is directly visible under the microscope. Sorrentanone (**7**) and pent-4-enyl quinone (**39**) are less active than *iso*-hexyl (**36**) and the benzyl derivative (**41**). This becomes apparent when analyzing the filament formation at a reduced concertation of 0.5 times MIC. Assays with sorrentanone (**7**) already show filamentation after 120 h, similar to the DMSO control, whereas this is hardly observed with derivatives **36** and **41**.

During the infection process, yeast to hyphae transition represents an early step in *Candida* biofilm formation. Given that the ability to form biofilms is another important feature of *Candida* pathogenicity, we next evaluated the antibiofilm activity of the compounds. All four tested compounds (**7**, **36**, **39**, **41**) displayed inhibition of biofilm formation in *C. albicans* ATCC10231 (data not shown). Sorrentanone (**7**) and pent-4-enyl quinone (**41**) possessed the highest inhibition with 60–77% at sub-inhibitory concentrations (0.5 times MIC). The outstanding performance of sorrentanone (**7**) was surprising, as we had expected a close correlation to the hyphal formation experiments expected. Therefore, the influence on filamentation is not directly related to the adhesion effect.

To further investigate potential mechanisms of action of the different sorrentanones, we performed an ergosterol biosynthesis assay.^21^ Ergosterol is an important component of fungal cell membranes, analogous to cholesterol in animal cells. Antifungal drugs can interfere with the biosynthesis of this membrane building block by binding to a specific fungal cytochrome P450 catalyzing the oxidation of lanosterol into ergosterol. Due to the different absorption of lanosterol and ergosterol, this enzymatic reaction or its inhibition can be monitored by UV spectrometry (Fig. 5c). For the tested compounds, sorrentanone (**7**) and derivatives **36** (*i*Hex), **39** (pent-4-enyl), **41** (benzyl) were most potent in reducing the total amount of ergosterol in *Candida* cells. In addition to the impact on filamentation and biofilm formation, this is an indication that sorrentanones target the cell membrane.

## Conclusions

In summary, we synthesized sorrentanone (**7**) and 14 ester-type derivatives (**28**–**41**) by either PIFA-mediated chemical or SorbC-catalyzed biocatalytic oxidation. Analysis of antimicrobial properties showed an improvement in activity against Gram-positive bacteria for compounds **28**–**41** compared to natural product **7**. In addition, the sorrentanone library also exhibits weak antifungal activity against various *Candida* species in combination with acceptable in vitro and in vivo cytotoxicity. Potential target analysis revealed the influence on the filamentation and biofilm production in *C. albicans*. In addition, the tested compounds were shown to target cell wall biosynthesis. Overall, sorrentanone and the synthesized ester analogs possess both antibacterial and antifungal activities expanding the pharmaceutical relevance of monomeric sorbicillinoids.

## Methods

### General methods

For instrumentation and material, see Supplementary Information including experimental procedures, supplementary figures and NMR spectra of new compounds (see Table S1, Figure S1-S67).

## Supplementary Information

Supplementary Information.

## Data Availability

All data generated and analyzed during this study are included in this article, its Supplementary Information and also available from the corresponding authors upon reasonable request.
