# Supplementary material for: Total synthesis of a sorrentanone ester library and evaluation of its antimicrobial potential
Source: Sci Rep. 2025 Jul 4;15:23877. doi: 10.1038/s41598-025-05360-y (PMC12227560; doi:10.1038/s41598-025-05360-y)
Supplement: Supplementary file 1 — Supplementary Information. [file 41598_2025_5360_MOESM1_ESM.pdf]

Total Synthesis of a Sorrentanone Ester Library and Evaluation of its Antimicrobial Potential

Tobias M. Milzarek[1,2,5], Finn H. Gattwinkel[1,4,5], Š. Bogojević[3,5], Dušan Milivojević[3], Sandra Vojnović[3], Tobias A. M. Gulder[1,2]* & Jasmina Nikodinovic-Runic[3]*

[1] Chair of Technical Biochemistry, Technical University of Dresden, Bergstraße 66, 01062 Dresden, Germany.
[2] Department of Natural Product Biotechnology, Helmholtz Institute for Pharmaceutical Reseaech Saarland (HIPS), Helmholtz Centre for Infection Research (HZI) and Department of Pharmacy at Saarland University, PharmaScienceHub (PSH), 66123 Saarbrücken, Germany.

[3] Institute of Molecular Genetics and Genetic Engineering, University of Belgrade, VojvodeStepe 444a, 11000 Belgrade, Serbia.

[4] Present Address: Institut für Chemie, Technische Universität Berlin, Straße des 17. Juni 115, 10623 Berlin, Germany

[5] These authors contributed equally

* Corresponding Author: tobias.gulder@helmholtz-hips.de, jasmine.nikodinovic@imgge.bg.ac.rs

Supplementary Information

Contents

1. GENERAL METHODS S2
2. CHEMICAL PROCEDURES S4
2.1 Synthesis of Sorrentanone (7) S4
2.2 Synthesis of Benzoates (14-26) S6
2.3 Optimization of Oxidation S14
2.4 Chemical Synthesis of Sorrentanone Analogs (28-41) S15
2.5 Enzymatic Synthesis of Sorrentanone Analogs (28-41) S20
3. BIOACTIVITY AND TOXICITY ASSAYS S21
4. NMR DATA S23
5. SUPPLEMENTARY REFERENCES S63

SUPPLEMENTARY TABLE S14

Supplementary Methods

1. General Methods

Reagents: Solvents for HPLC and MS analysis, such as acetonitrile and methanol, were purchased from Fisher Scientific and VWR in a purity of over 99% (HPLC-grade). Water was purified using a TKA GenPure water treatment system and deionized. Dry solvents such as diethyl ether, dichloromethane, methanol, tetrahydrofuran and toluene for procedures under inert atmosphere were prepared by distillation and drying over molecular sieves (3 Å or 4 Å). Commercial materials and other solvents were purchased at the highest commercial quality from the providers Acros Organics, Alfa Aesar, Carbolution, Carl Roth, Merck, Sigma Aldrich, VWR, TCI Chemicals and Thermo Fisher Scientific. Air- and moisture-sensitive reactions were performed under argon atmosphere using a Schlenk line. Before application, the flasks were repeatedly evacuated (external heating) and refilled with argon.

NMR: ^{1}H and ^{13}C Nuclear Magnetic Resonance Spectra (NMR) were recorded on Bruker AV-300 and AV-600 spectrometers at 298 K. The chemical shifts are given in δ-values (ppm) and are calibrated on the residual peak of the deuterated solvent ($CDCl_3$: δ_H = 7.26 ppm, δ_C = 77.0 ppm; MeOD-d_4: δ_H = 3.31 ppm, δ_C = 49.0 ppm; acetone-d_6: δ_H = 2.05 ppm, δ_C = 29.8 ppm). The coupling constants *J* are given in Hertz [Hz]. Following abbreviations were used for the allocation of signal multiplicities: bs – broad signal, s – singlet, d – doublet, dd – doublet of doublets, dq – doublet of quartets, ddt – doublet of doublets of triplets, t – triplet, td – triplet of doublets, tt – triplet of triplets, q – quartet, qt – quartet of triplets, p – pentet, sex – sextet, h – heptet, m – multiplet.

MS: Elektrospray-Ionisation Mass spectra (ESI-MS) were recorded on an Advion expressionL CMS system using a single-quadrupole mass analyzer, a Peak Scientific N118LA nitrogen generator, an Edwards RV12 high vacuum pump and a Jasco PU-1580 Intelligent HPLC Pump, or a LCQ Fleet ion trap system (Thermo Scientific), which was combined with an UltiMate 300 HPLC system. For high resolution mass spectrometry (HRMS) an Agilent mass spectrometer 6538 with atmospheric pressure chemical ionization (APCI), high resolution Q-TOF mass analyzer and microchannel plate detector was used.

Chromatography: Thin-layer chromatography (TLC) was performed on precoated plates of silica gel F254 (Merck) with UV detection at 254 and 365 nm. Column chromatography was performed on silica gel 60 Geduran® Si 60 (40–60 µm) (Merck). High Performance Liquid Chromatograms (HPLC) were recorded on a computer-controlled Jasco system including a UV-1575 Intelligent UV/VIS Detector, DG-2080-53 3-Line Degaser, two PU-1580 Intelligent HPLC Pumps, AS-1550 Intelligent Sampler, HG-1580-32 Dynamic Mixer. A Eurospher II 100-3 C18 A (150 × 4.6 mm) column with integrated precolumn manufactured by Knauer was used. The eluent system consisted of A = H_2O + 0.05% TFA, B = MeCN + 0.05% TFA. The analytical method used the following elution gradient: 0–1 min 5% B, 1–15 min to 95% B, 15–18 min 95% B, 18–

18.5 min to 5% B, 18.5–20 min 5% B with a flowrate of 1 mL/min. For medium pressure liquid chromatography (MPLC) the Reveleris® X2 MPLC system (Grace) was used together with Reverleris® Reverse Phase (RP) C18 columns (Grace) using UV-detection at 220 nm, 254 nm, and 280 nm. Isolation of the chemo-enzymatically produced compounds was carried out by semi-preparative HPLC controlled by a Jasco HPLC system consisting of an UV-1575 Intelligent UV/VIS Detector, two PU-2068 Intelligent prep. Pumps, a MIKA 1000 Dynamic Mixing Chamber (1000 µL Portmann Instruments AG Biel-Benken), a LC-NetII/ ADC, and a Rheodyne injection valve. The system was controlled by the Galaxie-Software and the eluent system consisted of: A = H_2O + 0.05% TFA and B = ACN + 0.05% TFA. A Eurosphere II 100-5 C18 A (250 x 16 mm) column with precolumn (30 x 16 mm) provided by Knauer was used as the stationary phase. General HPLC condition: gradient: 0–1 min 95% H_2O + 0.05% TFA (A) / 5% acetonitrile + 0.05% TFA (B), 1–55 min 5% A / 95% B, 55–56 min 5% A / 95% B, 56–58 min 95% A / 5% B, 58–60 min 95% A / 5% B, flow rate: 10 mL/min, running time: 60 min.

Optical rotataion: The specific rotation was measured with a PerkinElmer Model 341 LLC Polarimeter at 20 °C. The concentration for the specific rotation measurements is given in 10 mg/mL.

Recombinant protein production and purification: The bacterial strain *Escherichia coli* Δmtn was used as host to produce the monooxygenase SorbC. *E. coli* were grown in 2xYT media using 3 L Fernbach flasks. Glycerol stocks were used to inoculate starting cultures for production. The bacteria were grown at 37 °C in LB medium containing 50 µg/mL kanamycin sulfate overnight as preculture. The starting culture was further used to inoculate 2xYT (1:100) medium supplemented with the same concentration of antibiotic. When an OD_{600} = 0.5 was reached, the medium was cooled down and protein production was induced by adding 0.1 mM Isopropyl β-D-thiogalactoside (IPTG), followed by an overnight incubation at 16 °C with shaking (180 rpm). The cells were harvested by centrifugation and re-suspended in lysis buffer (50 mM NaH_2PO_4, 300 mM NaCl, 15 mM imidazole, 10% glycerol, pH 7.5). After sonification on ice and subsequent centrifugation (12000 rpm, 4 °C, 30 min), the supernatant was incubated with PureCube Ni-NTA Agarose (Cube Biotech) with shaking (100 rpm) for at least one hour. The suspension was applied to an empty 50 mL BioRad column, which was washed with one bed volume of lysis buffer. The protein was eluted with a higher concentrated imidazole buffer (50 mM NaH_2PO_4, 300 mM NaCl, 250 mM imidazole, 10% glycerol, pH 7.5). PD-10 columns (GE Healthcare) were used to exchange the buffer to the storage solution (50 mM NaH_2PO_4, 300 mM NaCl, 10% glycerol, pH 7.5). The protein concentration was determined photometrically using the Nanophotometer 330 (Implen) at 280 nm using the extinction coefficient of SorbC ε(280 nm) = 52420 M^{-1} cm^{-1} and a molecular weight of 61582.52 Da. The enzymatic assays were performed in phosphate buffer (50 mM, pH 8.0).

2. Chemical Procedures

2.1 Synthesis of Sorrentanone (7)

(2*E*,4*E*)-1-(2,4-Dihydroxy-3,6-dimethylphenyl)hexa-2,4-dien-1-one (9)

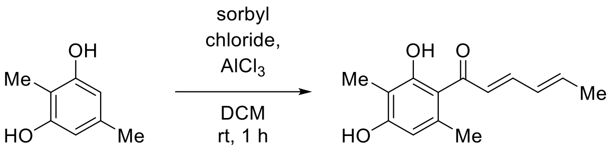

9

The reaction was performed under dry conditions using inert gas. 2,5-Dimethylresorcinol (**8**, 400 mg, 2.90 mmol, 1.0 equiv.) was dissolved in dry DCM (37 mL). The obtained solution was cooled down to 0 °C and aluminum chloride (425 mg, 3.18 mmol, 1.10 equiv.) and sorbyl chloride (416 mg, 3.18 mmol, 1.10 equiv.) was added slowly. After completion of the addition, the mixture was allowed to warm up to room temperature and was stirred for further 1.5 h. After dilution with 1 M HCl solution, the reaction mixture was extracted with DCM (3x). The combined organic layers were washed with brine, dried, filtered and concentrated in vacuo. The obtained residue was purified by column chromatography (*n*-pentane/EtOAc = 4:1) to give the desired product **9** as a yellow solid (532 mg, 2.29 mmol, 79%). Analytical data were in agreement with literature values.[1]

TLC: R_f (*n*-pentane/EtOAc = 4:1) = 0.37. **^{1}H-NMR** (300 MHz, MeOD-d_4): δ = 7.19 (dd, *J* = 15.0, 10.3 Hz, 1 H, C*H*), 6.63 (d, *J* = 15.1 Hz, 1 H, C*H*), 6.41–7.17 (m, 3 H, C*H*, Ar*H*), 2.34 (s, 3 H, CH_3), 2.00 (s, 3 H, CH_3), 1.87 (d, *J* = 6.3 Hz, 1 H, CH_3). **MS** (ESI+): *m/z* = 233.1 [M+H]$^+$.

Sorrentanone (7)

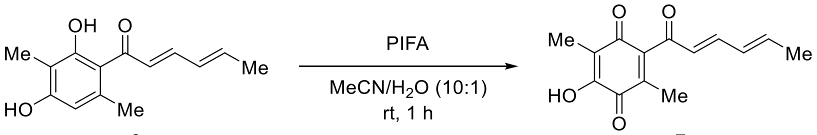

9 7

(2*E*,4*E*)-1-(2,4-Dihydroxy-3,6-dimethylphenyl)hexa-2,4-dien-1-one (**9**, 100 mg, 0.43 mmol, 1.00 equiv.) was dissolved in acetonitrile (8.6 mL) and water (0.86 mL). The obtained solution was cooled to 0 °C and PIFA (222 mg, 0.52 mmol, 1.20 equiv.) was added. The reaction mixture was allowed to warm up to room temperature and was stirred for another 1 h. After dilution with EtOAc and water, the reaction mixture was extracted with EtOAc (3x). The combined organic layers were washed with brine, dried, filtered and concentrated in vacuo. The obtained residue was purified by column chromatography (*n*-pentane/EtOAc = 3:1) to give the desired product **7** as an orange solid (48.0 mg, 0.20 mmol, 45%). Analytical data were in agreement with literature values.[2]

TLC: R_f (*n*-pentane/EtOAc = 3:1) = 0.21. **^{1}H-NMR** (300 MHz, $CDCl_3$): δ = 7.04–6.86 (m, 2 H, C*H*), 6.34–6.15 (m, 3 H, C*H*, O*H*), 1.96 (s, 3 H, CH_3), 1.95 (s, 3 H, CH_3), 1.88 (d, *J* = 5.3 Hz, 1 H, CH_3). **MS** (ESI+): *m/z* = 269.0 [M+Na]$^+$.

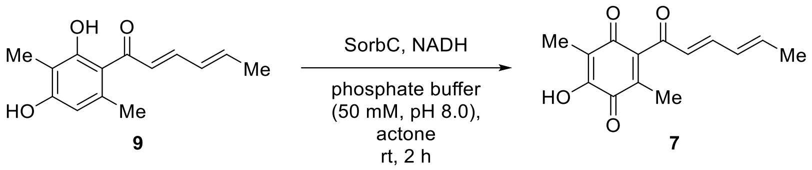

For comparison, the chemo-enzymatic synthesis of Sib[3]: To (2*E*,4*E*)-1-(2,4-dihydroxy-3,6-dimethylphenyl)hexa-2,4-dien-1-one (**9**, 20.0 mg, 86.2 µmol, 1.00 equiv.) dissolved in acetone (4 mL) was added to phosphate buffer (20 mL, 50 mM, pH = 8) with the enzyme SorbC (3.0 mL, 8.15 mg/mL in Tris-buffer). The reaction was started by addition of NADH (50.0 mg, 0.1 mmol, 1.30 equiv.) and incubated for 120 min at room temperature. The reaction mixture was washed with dichloromethane (3x 50 mL), then the aqueous and the organic phase were separately evaporated under reduced pressure. Purification by preperative HPLC (retention time: 19.0 min). The substrate **9** was re-isolated in 34% yield (6.8 mg, 29.3 µmol), based on which the desired product **7** was obtained in 29% yield (4.1 mg, 16.6 µmol, 19% without re-isolation of **9**).

^{1}H-NMR (300 MHz, MeOD-d_4): δ = 7.11 (dd, *J* = 15.7, 9.9 Hz, 1 H, C*H*), 6.37–6.34 (m, 2 H, C*H*), 6.19 (d, *J* = 15.7 Hz, 1 H, C*H*), 1.89–1.85 (m, 9 H, CH_3). **^{13}C-NMR** (75 MHz, MeOD-d_4): δ = 196.3, 188.0, 184.3, 155.1, 150.2, 144.4, 143.5, 138.6, 131.6, 129.3, 117.7, 19.1, 12.3, 7.7. **HRMS** (ESI+) *m/z* calcd for $C_{14}H_{15}O_4$ [M+H]$^+$: 247.0965; found: 247.0964.

2.2 Synthesis of Benzoates (14-26)

Methyl 2,4-dihydroy-3,6-dimethylbenzoate (14)

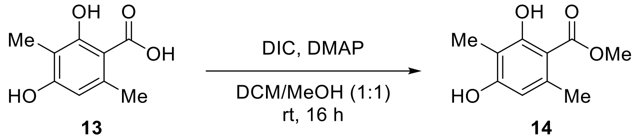

The reaction was performed under dry conditions. 2,4-Dihydroxy-3,6-dimethylbenzoeic acid (**13**, 200 mg, 1.10 mmol, 1.00 equiv.) was suspended in DCM (7.70 mL) and methanol (7.70 mL) was added. The obtained clear solution was cooled down to 0 °C followed by the addition of DMAP (13.0 mg, 0.11 mmol, 0.10 equiv.) and DIC (190 µL, 152 mg, 1.21 mmol, 1.10 equiv.). The reaction mixture was allowed to warm up to room temperature and was stirred for further 16 h. After dilution with 1 M HCl solution, the reaction mixture was extracted with EtOAc (3x). The combined organic layers were washed with brine, dried, filtered and concentrated in vacuo. The obtained residue was purified by column chromatography (*n*-pentane/EtOAc = 3:1) to give the desired product **14** as a colorless solid (148 mg, 0.75 mmol, 69%). Analytical data were in agreement with literature values.[4]

TLC: R_f (*n*-pentane/EtOAc = 3:1) = 0.47. **^{1}H-NMR** (300 MHz, $CDCl_3$): δ = 12.05 (bs, 1 H, O*H*), 6.21 (s, 1 H, Ar*H*), 5.06 (bs, 1 H, O*H*), 3.92 (s, 3 H, OC*H*$_3$), 2.46 (s, 3 H, C*H*$_3$), 2.10 (s, 3 H, C*H*$_3$). **MS** (ESI+): *m/z* = 197.0 $[M+H]^+$.

Ethyl 2,4-dihydroy-3,6-dimethylbenzoate (15)

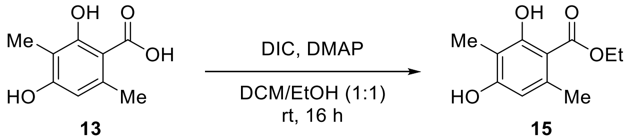

The reaction was performed under dry conditions. 2,4-Dihydroxy-3,6-dimethylbenzoeic acid (**13**, 200 mg, 1.10 mmol, 1.00 equiv.) was suspended in DCM (7.70 mL) and ethanol (7.70 mL) was added. The obtained clear solution was cooled down to 0 °C followed by the addition of DMAP (13.0 mg, 0.11 mmol, 0.10 equiv.) and DIC (190 µL, 152 mg, 1.21 mmol, 1.10 equiv.). The reaction mixture was allowed to warm up to room temperature and was stirred for further 16 h. After dilution with 1 M HCl solution, the reaction mixture was extracted with EtOAc (3x). The combined organic layers were washed with brine, dried, filtered and concentrated in vacuo. The obtained residue was purified by column chromatography (*n*-pentane/EtOAc = 3:1) to give the desired product **15** as a colorless solid (140 mg, 0.67 mmol, 61%).

TLC: R_f (*n*-pentane/EtOAc = 3:1) = 0.60. **^{1}H-NMR** (300 MHz, $CDCl_3$): δ = 12.13 (bs, 1 H, O*H*), 6.20 (s, 1 H, Ar*H*), 5.13 (bs, 1 H, O*H*), 4.39 (q, *J* = 7.1 Hz, 2 H, OC*H*$_2$CH$_3$), 2.47 (s, 3 H, C*H*$_3$), 2.10 (s, 3 H, C*H*$_3$), 1.41 (t, *J* = 7.1 Hz, 3 H, OCH$_2$C*H*$_3$). **^{13}C-NMR** (75 MHz, $CDCl_3$): δ = 172.3, 163.3, 158.1, 140.1, 110.7, 108.7, 105.5, 61.4, 24.3, 14.4, 7.8. **HRMS** (ESI+) *m/z* calcd for $C_{11}H_{14}O_4Na$ $[M+Na]^+$: 233.0784; found: 233.0778.

Propyl 2,4-dihydroy-3,6-dimethylbenzoate (16)

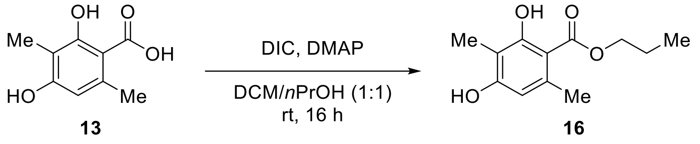

The reaction was performed under dry conditions. 2,4-Dihydroxy-3,6-dimethylbenzoeic acid (**13**, 200 mg, 1.10 mmol, 1.00 equiv.) was suspended in DCM (7.70 mL) and propanol (7.70 mL) was added. The obtained clear solution was cooled down to 0 °C followed by the addition of DMAP (13.0 mg, 0.11 mmol, 0.10 equiv.) and DIC (190 µL, 152 mg, 1.21 mmol, 1.10 equiv.). The reaction mixture was allowed to warm up to room temperature and was stirred for further 16 h. After dilution with 1 M HCl solution, the reaction mixture was extracted with EtOAc (3x). The combined organic layers were washed with brine, dried, filtered and concentrated in vacuo. The obtained residue was purified by column chromatography (*n*-pentane/EtOAc = 3:1) to give the desired product **16** as a colorless solid (140 mg, 0.67 mmol, 61%).

TLC: R_f (*n*-pentane/EtOAc = 3:1) = 0.68. **^{1}H-NMR** (300 MHz, $CDCl_3$): δ = 12.17 (bs, 1 H, O*H*), 6.21 (s, 1 H, Ar*H*), 5.11 (bs, 1 H, O*H*), 4.30 (t, *J* = 6.6 Hz, 2 H, $OCH_2CH_2CH_3$), 2.48 (s, 3 H, CH_3), 2.10 (s, 3 H, CH_3), 1.80 (h, *J* = 7.1 Hz, 2 H, $OCH_2CH_2CH_3$), 1.04 (t, *J* = 7.4 Hz, 3 H, $OCH_2CH_2CH_3$). **^{13}C-NMR** (75 MHz, $CDCl_3$): δ = 172.5, 163.4, 158.1, 140.3, 110.7, 108.7, 105.5, 67.2, 24.4, 22.1, 10.9, 7.8. **HRMS** (ESI+) *m/z* calcd for $C_{12}H_{16}O_4Na$ $[M+Na]^+$: 247.0941; found: 247.0936.

Butyl 2,4-dihydroy-3,6-dimethylbenzoate (17)

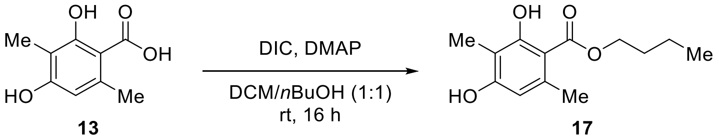

The reaction was performed under dry conditions. 2,4-Dihydroxy-3,6-dimethylbenzoeic acid (**13**, 200 mg, 1.10 mmol, 1.00 equiv.) was suspended in DCM (7.70 mL) and butanol (7.70 mL) was added. The obtained clear solution was cooled down to 0 °C followed by the addition of DMAP (13.0 mg, 0.11 mmol, 0.10 equiv.) and DIC (190 µL, 152 mg, 1.21 mmol, 1.10 equiv.). The reaction mixture was allowed to warm up to room temperature and was stirred for further 16 h. After dilution with 1 M HCl solution, the reaction mixture was extracted with EtOAc (3x). The combined organic layers were washed with brine, dried, filtered and concentrated in vacuo. The obtained residue was purified by column chromatography (*n*-pentane/EtOAc = 4:1) to give the desired product **17** as a colorless solid (176 mg, 0.74 mmol, 67%).

TLC: R_f (*n*-pentane/EtOAc = 4:1) = 0.68. **^{1}H-NMR** (300 MHz, $CDCl_3$): δ = 12.16 (bs, 1 H, O*H*), 6.20 (s, 1 H, Ar*H*), 5.10 (bs, 1 H, O*H*), 4.34 (t, *J* = 6.6 Hz, 2 H, $OCH_2CH_2CH_2CH_3$), 2.47 (s, 3 H, CH_3), 2.10 (s, 3 H, CH_3), 1.76 (m, 2 H, $OCH_2CH_2CH_2CH_3$), 1.47 (m, 2 H, $OCH_2CH_2CH_2CH_3$), 0.97 (t, *J* = 7.4 Hz, 3 H, $OCH_2CH_2CH_2CH_3$). **^{13}C-NMR** (75 MHz, $CDCl_3$): δ = 172.5, 163.4, 158.0, 140.3, 110.6, 108.7, 105.6, 65.3, 30.8, 24.4, 19.6, 13.8, 7.8. **HRMS** (ESI+) *m/z* calcd for $C_{13}H_{18}O_4Na$ $[M+Na]^+$: 261.1103; found: 261.1099.

Isobutyl 2,4-dihydroy-3,6-dimethylbenzoate (18)

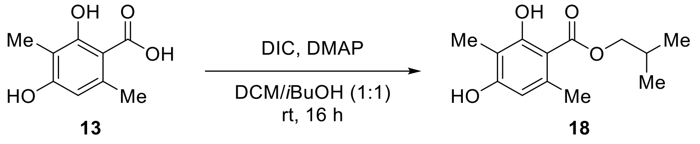

The reaction was performed under dry conditions. 2,4-Dihydroxy-3,6-dimethylbenzoeic acid (**13**, 200 mg, 1.10 mmol, 1.00 equiv.) was suspended in DCM (7.70 mL) and *iso*-butanol (7.70 mL) was added. The obtained clear solution was cooled down to 0 °C followed by the addition of DMAP (13.0 mg, 0.11 mmol, 0.10 equiv.) and DIC (190 µL, 152 mg, 1.21 mmol, 1.10 equiv.). The reaction mixture was allowed to warm up to room temperature and was stirred for further 16 h. After dilution with 1 M HCl solution, the reaction mixture was extracted with EtOAc (3x). The combined organic layers were washed with brine, dried, filtered and concentrated in vacuo. The obtained residue was purified by column chromatography (*n*-pentane/EtOAc = 10:1) to give the desired product **18** as a colorless solid (139 mg, 0.58 mmol, 53%).

TLC: R_f (*n*-pentane/EtOAc = 10:1) = 0.30. **^{1}H-NMR** (300 MHz, $CDCl_3$): δ = 12.19 (bs, 1 H, O*H*), 6.21 (s, 1 H, Ar*H*), 5.13 (bs, 1 H, O*H*), 4.14 (d, *J* = 6.5 Hz, 2 H, $OC\mathit{H_2}CH(CH_3)_2$), 2.50 (s, 3 H, $C\mathit{H_3}$), 2.18–2.02 (m, 4 H, $OCH_2C\mathit{H}(CH_3)_2$, $C\mathit{H_3}$), 1.03 (d, *J* = 6.7 Hz, 6 H, $OCH_2CH(C\mathit{H_3})_2$). **^{13}C-NMR** (75 MHz, $CDCl_3$): δ = 172.5, 163.5, 158.1, 140.2, 110.6, 108.7, 105.5, 71.9, 27.8, 24.6, 19.5, 7.8. **HRMS** (ESI+) *m/z* calcd for $C_{13}H_{18}O_4Na$ $[M+Na]^+$: 261.1097; found: 261.1095.

Pentyl 2,4-dihydroy-3,6-dimethylbenzoate (19)

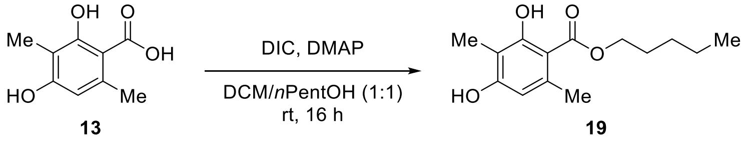

The reaction was performed under dry conditions. 2,4-Dihydroxy-3,6-dimethylbenzoeic acid (**13**, 200 mg, 1.10 mmol, 1.00 equiv.) was suspended in DCM (7.70 mL) and pentanol (7.70 mL) was added. The obtained clear solution was cooled down to 0 °C followed by the addition of DMAP (13.0 mg, 0.11 mmol, 0.10 equiv.) and DIC (190 µL, 152 mg, 1.21 mmol, 1.10 equiv.). The reaction mixture was allowed to warm up to room temperature and was stirred for further 16 h. After dilution with 1 M HCl solution, the reaction mixture was extracted with EtOAc (3x). The combined organic layers were washed with brine, dried, filtered and concentrated in vacuo. The obtained residue was purified by column chromatography (*n*-pentane/EtOAc = 4:1) to give the desired product **19** as a colorless solid (176 mg, 0.69 mmol, 63%).

TLC: R_f (*n*-pentane/EtOAc = 4:1) = 0.72. **^{1}H-NMR** (300 MHz, $CDCl_3$): δ = 12.15 (bs, 1 H, O*H*), 6.21 (s, 1 H, Ar*H*), 5.04 (bs, 1 H, O*H*), 4.33 (t, *J* = 6.6 Hz, 2 H, $OC\mathit{H_2}CH_2CH_2CH_2CH_3$), 2.48 (s, 3 H, $C\mathit{H_3}$), 2.10 (s, 3 H, $C\mathit{H_3}$), 1.78 (pen, 2 H, $OCH_2C\mathit{H_2}CH_2CH_2CH_3$), 1.50–1.30 (m, 4 H, $OCH_2CH_2C\mathit{H_2}C\mathit{H_2}CH_3$), 0.92 (t, *J* = 7.5 Hz, 3 H, $OCH_2CH_2CH_2CH_2C\mathit{H_3}$). **^{13}C-NMR** (75 MHz,

$CDCl_3$): δ = 172.4, 163.4, 158.0, 140.3, 110.6, 108.6, 105.6, 65.6, 28.5, 28.4, 24.4, 22.4, 14.1, 7.8. **HRMS** (ESI+) *m/z* calcd for $C_{14}H_{20}O_4Na$ $[M+Na]^+$: 275.1254; found: 275.1250.

Isopentyl 2,4-dihydroy-3,6-dimethylbenzoate (20)

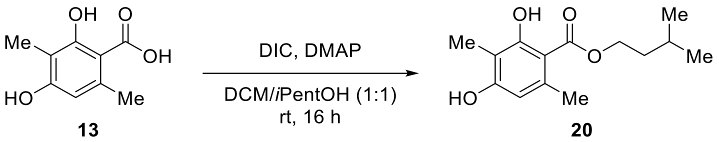

The reaction was performed under dry conditions. 2,4-Dihydroxy-3,6-dimethylbenzoeic acid (**13**, 200 mg, 1.10 mmol, 1.00 equiv.) was suspended in DCM (7.70 mL) and *iso*-pentanol (7.70 mL) was added. The obtained clear solution was cooled down to 0 °C followed by the addition of DMAP (13.0 mg, 0.11 mmol, 0.10 equiv.) and DIC (190 µL, 152 mg, 1.21 mmol, 1.10 equiv.). The reaction mixture was allowed to warm up to room temperature and was stirred for further 16 h. After dilution with 1 M HCl solution, the reaction mixture was extracted with EtOAc (3x). The combined organic layers were washed with brine, dried, filtered and concentrated in vacuo. The obtained residue was purified by column chromatography (*n*-pentane/EtOAc = 10:1) to give the desired product **20** as a colorless solid (130 mg, 0.52 mmol, 47%).

TLC: R_f (*n*-pentane/EtOAc = 10:1) = 0.35. **^{1}H-NMR** (300 MHz, $CDCl_3$): δ = 12.15 (bs, 1 H, O*H*), 6.20 (s, 1 H, Ar*H*), 5.07 (bs, 1 H, O*H*), 4.37 (t, *J* = 6.7 Hz, 2 H, $OCH_2CH_2CH(CH_3)_2$), 2.47 (s, 3 H, CH_3), 2.10 (s, 3 H, CH_3), 1.83–1.63 (m, 3 H, $OCH_2CH_2CH(CH_3)_2$), 0.96 (d, *J* = 6.4 Hz, 6 H, $OCH_2CH_2CH(CH_3)_2$). **^{13}C-NMR** (75 MHz, $CDCl_3$): δ = 172.4, 163.4, 158.0, 140.3, 110.6, 108.7, 105.6, 64.1, 37.4, 25.3, 24.4, 22.6, 7.8. **HRMS** (ESI+) *m/z* calcd for $C_{14}H_{20}O_4Na$ $[M+Na]^+$: 275.1254; found: 261.1249.

Hexyl 2,4-dihydroy-3,6-dimethylbenzoate (21)

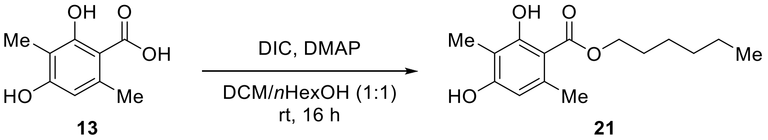

The reaction was performed under dry conditions. 2,4-Dihydroxy-3,6-dimethylbenzoeic acid (**13**, 200 mg, 1.10 mmol, 1.00 equiv.) was suspended in DCM (7.70 mL) and hexanol (7.70 mL) was added. The obtained clear solution was cooled down to 0 °C followed by the addition of DMAP (13.0 mg, 0.11 mmol, 0.10 equiv.) and DIC (190 µL, 152 mg, 1.21 mmol, 1.10 equiv.). The reaction mixture was allowed to warm up to room temperature and was stirred for further 16 h. After dilution with 1 M HCl solution, the reaction mixture was extracted with EtOAc (3x). The combined organic layers were washed with brine, dried, filtered and concentrated in vacuo. The obtained residue was purified by column chromatography (*n*-pentane/EtOAc = 30:1) to give the desired product **21** as a colorless solid (176 mg, 0.69 mmol, 63%).

TLC: R_f (*n*-pentane/EtOAc = 30:1) = 0.15. **^{1}H-NMR** (300 MHz, $CDCl_3$): δ = 12.16 (bs, 1 H, O*H*), 6.20 (s, 1 H, Ar*H*), 5.10 (bs, 1 H, O*H*), 4.33 (t, *J* = 6.6 Hz, 2 H, $OC\mathit{H}_2CH_2CH_2CH_2CH_2CH_3$), 2.48 (s, 3 H, $C\mathit{H}_3$), 2.10 (s, 3 H, $C\mathit{H}_3$), 1.77 (p, 2 H, $OCH_2C\mathit{H}_2CH_2CH_2CH_2CH_3$), 1.52–1.20 (m, 6 H, $OCH_2CH_2C\mathit{H}_2C\mathit{H}_2C\mathit{H}_2CH_3$), 0.92–0.76 (m, 3 H, $OCH_2CH_2CH_2CH_2CH_2C\mathit{H}_3$). **^{13}C-NMR** (75 MHz, $CDCl_3$): δ = 172.5, 163.4, 158.0, 140.3, 110.6, 108.7, 105.6, 65.7, 31.5, 28.7, 26.0, 24.4, 22.7, 14.1, 7.8. **HRMS** (ESI+) *m/z* calcd for $C_{15}H_{22}O_4Na$ $[M+Na]^+$: 289.1410; found: 289.1405.

Isohexyl 2,4-dihydroy-3,6-dimethylbenzoate (22)

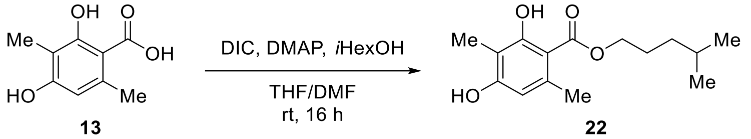

13 **22**

The reaction was performed under dry conditions. 2,4-Dihydroxy-3,6-dimethylbenzoeic acid (**13**, 200 mg, 1.10 mmol, 1.00 equiv.) was suspended in THF (7.7 mL) and isohexylalcohol (1.00 mL, 813 mg, 7.96 mmol, 7.25 equiv.). DMF (excess) was added to generate a clear reaction solution. The reaction mixture was cooled down to 0 °C followed by the addition of DMAP (13.0 mg, 0.11 mmol, 0.10 equiv.) and DIC (190 µL, 152 mg, 1.21 mmol, 1.10 equiv.). The reaction mixture was allowed to warm up to room temperature and was stirred for further 16 h. After dilution with 1 M HCl solution, the reaction mixture was extracted with EtOAc (3x). The combined organic layers were washed with brine, dried, filtered and concentrated in vacuo. The obtained residue was purified by MPLC (C18-12g cartridge, 30 mL/min flow rate, 0-2 min 5% MeCN, 2-37 min 5-95% MeCN, 37-40 min 95% MeCN) to give the desired product **22** as a colorless solid (187 mg, 0.70 mmol, 64%).

^{1}H-NMR (300 MHz, $CDCl_3$): δ = 12.16 (bs, 1 H, O*H*), 6.21 (s, 1 H, Ar*H*), 5.05 (bs, 1 H, O*H*), 4.32 (t, *J* = 6.7 Hz, 2 H, $OC\mathit{H}_2CH_2CH_2CH(CH_3)_2$), 2.48 (s, 3 H, $C\mathit{H}_3$), 2.10 (s, 3 H, $C\mathit{H}_3$), 1.81–1.77 (m, 2 H, $OCH_2C\mathit{H}_2CH_2CH(CH_3)_2$), 1.60 (m, 1 H, $OCH_2CH_2CH_2C\mathit{H}(CH_3)_2$), 1.35–1.28 (m, 2 H, $OCH_2CH_2C\mathit{H}_2CH(CH_3)_2$), 0.91 (d, *J* = 6.6 Hz, 2 H, $OCH_2CH_2CH_2CH(C\mathit{H}_3)_2$). **^{13}C-NMR** (75 MHz, $CDCl_3$): δ = 172.5, 163.4, 158.1, 140.3, 110.7, 108.7, 105.5, 65.9, 35.4, 27.9, 26.6, 24.4, 22.6, 7.8. **HRMS** (ESI+) *m/z* calcd for $C_{15}H_{22}O_4Na$ $[M+Na]^+$: 289.1410; found: 289.1406.

But-3-en-1-yl 2,4-dihydroy-3,6-dimethylbenzoate (23)

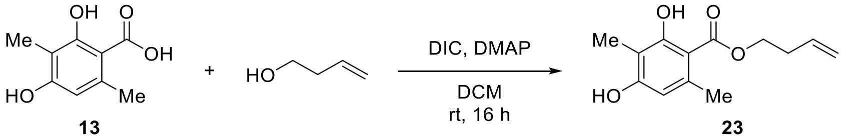

13 **23**

The reaction was performed under dry conditions. 2,4-Dihydroxy-3,6-dimethylbenzoeic acid (**13**, 200 mg, 1.10 mmol, 1.00 equiv.) was suspended in DCM (7.70 mL) and but-3-enol (7.70 mL) was added. The obtained clear solution was cooled down to 0 °C followed by the addition of DMAP (13.0 mg, 0.11 mmol, 0.10 equiv.) and DIC (190 µL, 152 mg, 1.21 mmol, 1.10 equiv.). The reaction mixture was allowed to warm up to room temperature and was stirred for further 16 h. After dilution with 1 M HCl solution, the reaction mixture was

extracted with EtOAc (3x). The combined organic layers were washed with brine, dried, filtered and concentrated in vacuo. The obtained residue was purified by column chromatography (*n*-pentane/EtOAc = 20:1) to give the desired product **23** as a colorless solid (142 mg, 0.61 mmol, 55%).

TLC: R_f (*n*-pentane/EtOAc = 20:1) = 0.11. **^{1}H-NMR** (300 MHz, $CDCl_3$): δ = 12.09 (bs, 1 H, O*H*), 6.20 (s, 1 H, Ar*H*), 5.85 (ddt, *J* = 17.0, 10.3, 6.7 Hz, 1 H, $OCH_2CH_2CH{=}CH_2$), 5.21 (bs, 1 H, O*H*), 5.17 (dd, *J* = 17.1, 1.6 Hz, 1 H, $OCH_2CH_2CH{=}CH_2$), 5.12 (dd, *J* = 10.2, 1.5 Hz, 1 H, $OCH_2CH_2CH{=}CH_2$), 4.40 (t, *J* = 6.6 Hz, 2 H, $OCH_2CH_2CH{=}CH_2$), 2.53 (qt, *J* = 6.6, 1.4 Hz, 2 H, $OCH_2CH_2CH{=}CH_2$), 2.46 (s, 3 H, CH_3), 2.10 (s, 3 H, CH_3). **^{13}C-NMR** (75 MHz, $CDCl_3$): δ = 172.3, 163.4, 158.2, 140.5, 134.2, 117.7, 110.7, 108.7, 105.4, 64.6, 33.2, 24.5, 7.8. **HRMS** (ESI+) *m/z* calcd for $C_{13}H_{16}O_4Na$ $[M+Na]^+$: 259.0941; found: 259.0937.

But-3-in-1-yl 2,4-dihydroy-3,6-dimethylbenzoate (24)

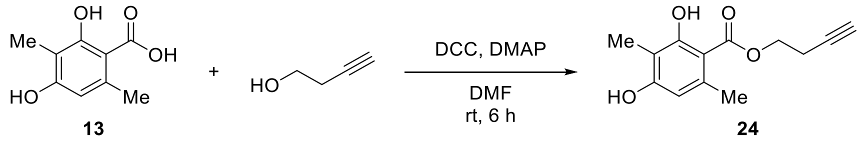

The reaction was performed under dry conditions. 2,4-Dihydroxy-3,6-dimethylbenzoeic acid (**13**, 200 mg, 1.10 mmol, 1.00 equiv.) was suspended in DMF (7.70 mL) and but-3-inol (1.00 mL, 927 mg, 13.2 mmol, 12.0 equiv.) was added. The obtained clear solution was cooled down to 0 °C followed by the addition of DMAP (13.0 mg, 0.11 mmol, 0.10 equiv.) and DCC (249 mg, 1.21 mmol, 1.10 equiv.). The reaction mixture was allowed to warm up to room temperature and was stirred for further 16 h. After dilution with 1 M HCl solution, the reaction mixture was extracted with EtOAc (3x). The combined organic layers were washed with brine, dried, filtered and concentrated in vacuo. The obtained residue was purified by column chromatography (*n*-pentane/EtOAc = 10:1) to give the desired product **24** as a colorless solid (91.0 mg, 0.39 mmol, 36%).

TLC: R_f (*n*-pentane/EtOAc = 10:1) = 0.21. **^{1}H-NMR** (300 MHz, $CDCl_3$): δ = 11.97 (s, 1 H, O*H*), 6.22 (s, 1 H, Ar*H*), 5.13 (bs, 1 H, O*H*), 4.44 (t, *J* = 6.5 Hz, 2 H, OCH_2CH_2CCH), 2.67 (td, *J* = 6.5, 2.7 Hz, 2 H, OCH_2CH_2CCH), 2.51 (s, 3 H, CH_3), 2.10 (s, 3 H, CH_3), 2.03 (t, *J* = 2.6 Hz, 1 H, OCH_2CH_2CCH). **^{13}C-NMR** (151 MHz, MeOD-d_4): δ = 173.3, 164.4, 161.7, 141.3, 111.6, 109.9, 104.8, 81.4, 71.2, 64.3, 24.7, 19.6, 7.9. **HRMS** (ESI+) *m/z* calcd for $C_{13}H_{14}O_4Na$ $[M+Na]^+$: 257.0784; found: 257.0784.

Pent-4-en-1-yl 2,4-dihydroy-3,6-dimethylbenzoate (25)

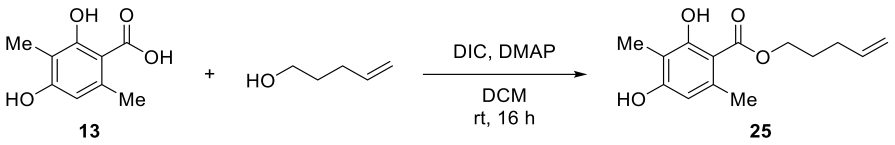

The reaction was performed under dry conditions. 2,4-Dihydroxy-3,6-dimethylbenzoeic acid (**13**, 200 mg, 1.10 mmol, 1.00 equiv.) was suspended in DCM (7.70 mL) and pent-4-enol (7.70 mL) was added. The obtained clear solution was cooled down to 0 °C followed by the addition of DMAP (13.0 mg, 0.11 mmol, 0.10 equiv.) and DIC (190 µL, 152 mg, 1.21 mmol, 1.10 equiv.). The reaction mixture was allowed to warm up to room temperature and was stirred for further 16 h. After dilution with 1 M HCl solution, the reaction mixture was extracted with EtOAc (3x). The combined organic layers were washed with brine, dried, filtered and concentrated in vacuo. The obtained residue was purified by column chromatography (*n*-pentane/EtOAc = 20:1) to give the desired product **25** as a colorless solid (176 mg, 0.70 mmol, 64%).

TLC: R_f (*n*-pentane/EtOAc = 20:1) = 0.13. **^{1}H-NMR** (300 MHz, $CDCl_3$): δ = 12.13 (bs, 1 H, O*H*), 6.21 (s, 1 H, Ar*H*), 5.83 (ddt, *J* = 17.0, 10.2, 6.7 Hz, 1 H, $OCH_2CH_2CH_2CH{=}CH_2$), 5.31 (bs, 1 H, O*H*), 5.07 (dq, *J* = 17.1, 1.6 Hz, 1 H, $OCH_2CH_2CH_2CH{=}CH_2$), 5.02 (dq, *J* = 10.2, 1.4 Hz, 1 H, $OCH_2CH_2CH_2CH{=}CH_2$), 4.35 (t, *J* = 6.6 Hz, 2 H, $OCH_2CH_2CH_2CH{=}CH_2$), 2.48 (s, 3 H, CH_3), 2.25–2.18 (m, 2 H, $OCH_2CH_2CH_2CH{=}CH_2$), 2.10 (s, 3 H, CH_3), 1.91–1.84 (m, 2 H, $OCH_2CH_2CH_2CH{=}CH_2$). **^{13}C-NMR** (75 MHz, $CDCl_3$): δ = 172.4, 163.4, 158.2, 140.2, 137.3, 115.7, 110.7, 108.8, 105.4, 64.8, 30.4, 27.9, 24.4, 7.8. **HRMS** (ESI+) *m/z* calcd for $C_{14}H_{18}O_4Na$ $[M+Na]^+$: 273.1097; found: 273.1093.

Pent-3-in-1-yl 2,4-dihydroy-3,6-dimethylbenzoate (26)

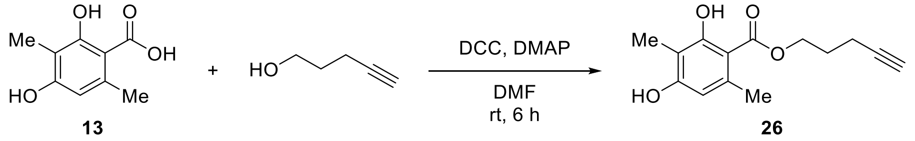

The reaction was performed under dry conditions. 2,4-Dihydroxy-3,6-dimethylbenzoeic acid (**13**, 200 mg, 1.10 mmol, 1.00 equiv.) was suspended in DMF (7.70 mL) and pent-4-inol (1.00 mL, 904 mg, 10.8 mmol, 9.80 equiv.) was added. The obtained clear solution was cooled down to 0 °C followed by the addition of DMAP (13.0 mg, 0.11 mmol, 0.10 equiv.) and DCC (249 mg, 1.21 mmol, 1.10 equiv.). The reaction mixture was allowed to warm up to room temperature and was stirred for further 16 h. After dilution with 1 M HCl solution, the reaction mixture was extracted with EtOAc (3x). The combined organic layers were washed with brine, dried, filtered and concentrated in vacuo. The obtained residue was purified by column chromatography (*n*-pentane/EtOAc = 10:1) to give the desired product **26** as a colorless solid (91.0 mg, 0.36 mmol, 33%).

TLC: R_f (*n*-pentane/EtOAc = 10:1) = 0.28. **^{1}H-NMR** (300 MHz, $CDCl_3$): δ = 12.09 (s, 1 H, O*H*), 6.21 (s, 1 H, Ar*H*), 5.07 (bs, 1 H, O*H*), 4.46 (t, *J* = 6.5 Hz, 2 H, $OCH_2CH_2CH_2CCH$), 2.47 (s, 3 H, CH_3), 2.38 (td, *J* = 7.0, 2.7 Hz, 2 H, $OCH_2CH_2CH_2CCH$), 2.10 (s, 3 H, CH_3), 2.06–1.95 (m, 3 H, $OCH_2CH_2CH_2CCH$). **^{13}C-NMR** (75 MHz, $CDCl_3$): δ = 172.3, 163.5, 158.2, 140.2, 110.7, 108.7, 105.3, 82.8, 69.5, 64.0, 27.6, 24.4, 15.7, 7.8. **HRMS** (ESI+) *m/z* calcd for $C_{14}H_{16}O_4Na$ [M+Na]$^+$: 271.0941; found: 271.0943.

Benzyl 2,4-dihydroy-3,6-dimethylbenzoate (27)

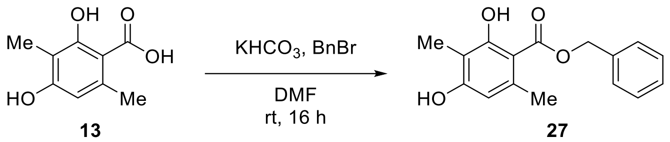

The reaction was performed under dry conditions. 2,4-Dihydroxy-3,6-dimethylbenzoeic acid (**13**, 100 mg, 0.55 mmol, 1.00 equiv.) was suspended in DMF (7.70 mL) and potassium hydrogen carbonate (66.0 mg, 0.66 mmol, 1.20 equiv.) was added. The obtained suspension was stirred at room temperature for 5 min followed by the addition of benzyl bromide (113 mg, 0.66 mmol, 1.20 equiv.). The reaction mixture was stirred for further 16 h at room temperature. After dilution with saturated NH_4Cl solution, the reaction mixture was extracted with EtOAc (3x). The combined organic layers were washed with brine, dried, filtered and concentrated in vacuo. The obtained residue was purified by column chromatography (*n*-pentane/EtOAc = 6:1) to give the desired product **27** as a colorless solid (130 mg, 0.48 mmol, 87%).

TLC: R_f (*n*-pentane/EtOAc = 6:1) = 0.32. **^{1}H-NMR** (300 MHz, $CDCl_3$): δ = 12.04 (s, 1 H, O*H*), 7.47-7.31 (m, 5 H, Ar*H*), 6.19 (s, 1 H, Ar*H*), 5.38 (s, 2 H, OCH_2), 5.20 (bs, 1 H, O*H*), 2.44 (s, 3 H, CH_3), 2.10 (s, 3 H, CH_3). **^{13}C-NMR** (75 MHz, $CDCl_3$): δ = 172.0, 163.5, 158.2, 140.4, 135.6, 128.8, 128.6, 110.7, 108.7, 105.3, 67.2, 24.6, 7.8. **HRMS** (ESI+) *m/z* calcd for $C_{16}H_{16}O_4Na$ [M+Na]$^+$: 295.0941; found: 295.0935.

2.3 Optimization of Oxidation

The optimization of the oxidation of esters into the corresponding was performed with butyl 2,4-dihydroy-3,6-dimethylbenzoate (**17**) serving as model substrate. Each optimization reaction was carried out on 0.20 mmol scale (50.0 m).

Supplementary Table

Tab. S1 Optimization of the oxidation using butyl 2,4-dihydroy-3,6-dimethylbenzoate (**17**).

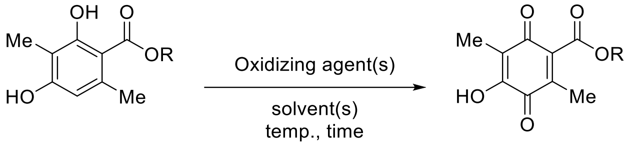

#	Oxidizing agent(s)	Solvent(s)	Temp.	Time	Yield / Observation
1[1]	PIFA (1.2 equiv.)	MeCN/H_2O = 10:1	rt	1 h	10%
2	PIFA (1.2 equiv.)	MeCN/H_2O = 10:1	100 °C	1 h	decomposition
3	PIFA (2.4 equiv.)	MeCN/H_2O = 10:1	rt	1 h	decomposition
4[5]	IBX (3.0 equiv.)	EtOAc	80 °C	4 h	decomposition
5[2]	Frémy's salt (3.6 equiv.)	acetone/H_2O = 1:1	rt	2 h	no reaction
6[6]	Salcomine (10 mol%), O_2 (atm)	MeCN	rt	16 h	no reaction
7	MnO_2 (4.0 equiv.), TBHP (6.0 equiv.)	acetone	60 °C	16 h	no reaction

PIFA: (bis(trifluoroacetoxy)iodo)benzene, IBX: 2-iodoxybenzoic acid, TBHP: *tert*-butyl hydroperoxide.

Each reaction was followed carefully by TLC (starting material: R_f (*n*-pentane/EtOAc = 4:1) = 0.68; product: R_f (cyclohexane/EtOAc = 20:1 + 1% AcOH) = 0.13). For entry #1, TLC indicated the formation of sorrentanone analog **31** after 1 h. Upscaling of reaction #1 allowed isolation of butyl sorrentanone **31** (see Chapter 2.4). Monitoring of the product spot (**31**) in reaction #2 indicated the generation of the desired product after 10 min. However, longer reaction time led to the disappearance of the product signal, which indicates a decomposition of the product under these reaction conditions. Similar results were obtained for entry #3, in which PIFA was added in two portions (2x 1.2 equiv.). After the addition of the first portion, the product signal was clearly observed. Addition of the second portion after 30 min, again led to decomposition of the product. Switching to other oxidation protocols known from the literature (entry #4-7) showed no improvement compared to PIFA oxidation 1.

2.4 Chemical Synthesis of Sorrentanone Analogs (28-41)

General Protocol for Chemical Oxidation

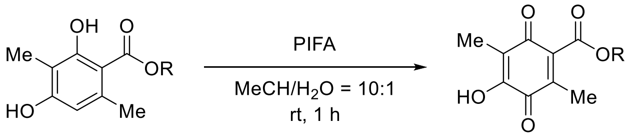

The corresponding benzoate (1.00 equiv.) was dissolved in MeCN (20 mL/mmol) and water (2 mL/mmol). The obtained reaction solution was cooled down to 0 °C and PIFA (1.05-1.20 equiv.) was added portion wise. The reaction mixture was allowed to warm up to room temperature and stirred for another hour. After dilution with EtOAc and water, the reaction mixture was extracted with EtOAc (3x). The combined organic layers were washed with brine, dried, filtered and concentrated in vacuo. The obtained residue was purified by preparative HPLC.

Methyl 4-hydroxy-2,5-dimethyl-3,6-dioxocyclohexa-1,4-diene-1-carboxylate (28)

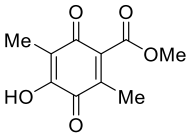

Following the general protocol on 0.41 mmol scale (**14**), benzochinone **28** was obtained as an orange solid (10.0 mg, 50.0 µmol, 12%).

TLC: R_f (cyclohexane/EtOAc = 3:1) = 0.19. **^{1}H-NMR** (300 MHz, $CDCl_3$): δ = 6.92 (bs, 1 H, O*H*), 3.91 (s, 3 H, OC*H*$_3$), 2.05 (s, 3 H, C*H*$_3$), 1.95 (s, 3 H, C*H*$_3$). **^{13}C-NMR** (75 MHz, $CDCl_3$): δ = 184.3, 183.4, 164.7, 151.2, 138.6, 137.7, 117.4, 52.9, 12.7, 8.1. **HRMS** (ESI+) *m/z* calcd for $C_{10}H_{10}O_5Na$ $[M+Na]^+$: 233.0420; found: 233.0424.

Ethyl 4-hydroxy-2,5-dimethyl-3,6-dioxocyclohexa-1,4-diene-1-carboxylate (29)

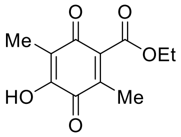

Following the general protocol on 0.38 mmol scale (**15**), benzochinone **29** was obtained as an orange solid (10.0 mg, 50.0 µmol, 12%).

TLC: R_f (cyclohexane/EtOAc = 7:1 + 5% MeOH) = 0.14. **^{1}H-NMR** (300 MHz, $CDCl_3$): δ = 6.91 (bs, 1 H, O*H*), 4.39 (q, *J* = 7.1 Hz, 2 H, OC*H*$_2$CH$_3$), 2.06 (s, 3 H, C*H*$_3$), 1.95 (s, 3 H, C*H*$_3$), 1.37 (t, *J* = 7.1 Hz, 3 H, OCH$_2$C*H*$_3$). **^{13}C-NMR** (75 MHz, $CDCl_3$): δ = 184.4, 183.5, 164.2, 151.2, 138.8, 137.3, 117.4, 62.3, 14.3, 12.6, 8.1. **HRMS** (ESI+) *m/z* calcd for $C_{11}H_{12}O_5Na$ $[M+Na]^+$: 247.0582; found: 247.0586.

Propyl 4-hydroxy-2,5-dimethyl-3,6-dioxocyclohexa-1,4-diene-1-carboxylate (30)

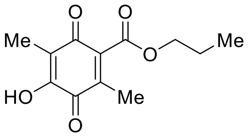

Following the general protocol on 0.36 mmol scale (**16**), benzochinone **30** was obtained as an orange solid (5.00 mg, 20.0 µmol, 6%).

TLC: R_f (cyclohexane/EtOAc = 7:1 + 5% MeOH) = 0.19. **^{1}H-NMR** (300 MHz, $CDCl_3$): δ = 6.90 (bs, 1 H, O*H*), 4.30 (t, *J* = 6.7 Hz, 2 H, $OCH_2CH_2CH_3$), 2.06 (s, 3 H, CH_3), 1.95 (s, 3 H, CH_3), 1.76 (sex, *J* = 7.2 Hz, 2 H, $OCH_2CH_2CH_3$), 0.99 (t, *J* = 7.4 Hz, 3 H, $OCH_2CH_2CH_3$). **^{13}C-NMR** (75 MHz, $CDCl_3$): δ = 184.4, 183.5, 164.4, 151.2, 138.9, 137.3, 117.4, 67.9, 22.1, 12.6, 10.5, 8.1. **HRMS** (ESI+) *m/z* calcd for $C_{12}H_{14}O_5Na$ $[M+Na]^+$: 261.0733; found: 261.0738.

Butyl 4-hydroxy-2,5-dimethyl-3,6-dioxocyclohexa-1,4-diene-1-carboxylate (31)

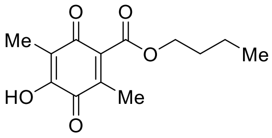

Following the general protocol on 0.34 mmol scale (**17**), benzochinone **31** was obtained as an orange oil (7.00 mg, 30.0 µmol, 10%).

TLC: R_f (cyclohexane/EtOAc = 20:1 + 1% AcOH) = 0.13. **^{1}H-NMR** (300 MHz, $CDCl_3$): δ = 6.90 (bs, 1 H, O*H*), 4.33 (t, *J* = 6.7 Hz, 2 H, $OCH_2CH_2CH_2CH_3$), 2.06 (s, 3 H, CH_3), 1.95 (s, 3 H, CH_3), 1.71 (p, *J* = 6.9 Hz, 2 H, $OCH_2CH_2CH_2CH_3$), 1.43 (sex, *J* = 7.0 Hz, 2 H, $OCH_2CH_2CH_2CH_3$), 0.96 (t, *J* = 7.4 Hz, 3 H, $OCH_2CH_2CH_2CH_3$). **^{13}C-NMR** (75 MHz, $CDCl_3$): δ = 184.4, 183.5, 164.4, 151.2, 138.9, 137.3, 117.4, 66.1, 30.7, 19.2, 13.8, 12.6, 8.1. **HRMS** (ESI+) *m/z* calcd for $C_{13}H_{16}O_5Na$ $[M+Na]^+$: 275.0890; found: 275.0893.

Isobutyl 4-hydroxy-2,5-dimethyl-3,6-dioxocyclohexa-1,4-diene-1-carboxylate (32)

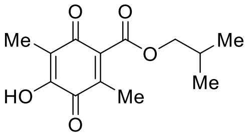

Following the general protocol on 0.34 mmol scale (**18**), benzochinone **32** was obtained as an orange oil (13.0 mg, 50.0 µmol, 15%).

TLC: R_f (cyclohexane/EtOAc = 20:1 + 1% AcOH) = 0.15. **^{1}H-NMR** (600 MHz, $CDCl_3$): δ = 6.93 (bs, 1 H, O*H*), 4.12 (d, *J* = 6.7 Hz, 2 H, $OCH_2CH(CH_3)_2$), 2.09–1.99 (m, 4 H, $OCH_2CH(CH_3)_2$, CH_3), 1.95 (s, 3 H, CH_3), 0.98 (d, *J* = 6.7 Hz, 6 H, $OCH_2CH(CH_3)_2$). **^{13}C-NMR** (151 MHz, $CDCl_3$): δ = 184.4, 183.5, 164.4, 151.2, 138.9, 137.3, 117.4, 27.9, 19.2, 12.6, 8.1. **HRMS** (ESI+) *m/z* calcd for $C_{13}H_{16}O_5Na$ $[M+Na]^+$: 275.0890; found: 275.0893.

Pentyl 4-hydroxy-2,5-dimethyl-3,6-dioxocyclohexa-1,4-diene-1-carboxylate (33)

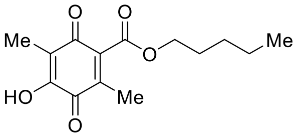

Following the general protocol on 0.32 mmol scale (**19**), benzochinone **33** was obtained as an orange oil (12.0 mg, 40.0 µmol, 14%).

TLC: R_f (cyclohexane/EtOAc = 10:1 + 1% AcOH) = 0.17. **^{1}H-NMR** (600 MHz, $CDCl_3$): δ = 6.92 (bs, 1 H, O*H*), 4.32 (t, *J* = 6.8 Hz, 2 H, $OCH_2CH_2CH_2CH_2CH_3$), 2.05 (s, 3 H, CH_3), 1.95 (s, 3 H, CH_3), 1.73 (p, *J* = 6.8 Hz, 2 H, $OCH_2CH_2CH_2CH_2CH_3$), 1.42–1.31 (m, 4 H, $OCH_2CH_2CH_2CH_2CH_3$), 0.91 (t, *J* = 7.1 Hz, 3 H, $OCH_2CH_2CH_2CH_2CH_3$). **^{13}C-NMR** (151 MHz, $CDCl_3$): δ = 184.4, 183.5, 164.4, 151.2, 138.9, 137.3, 117.4, 66.4, 28.3, 28.0, 22.4, 14.1, 12.6, 8.1. **HRMS** (ESI+) *m/z* calcd for $C_{14}H_{18}O_5Na$ $[M+Na]^+$: 289.1046; found: 289.1053.

Isopentyl 4-hydroxy-2,5-dimethyl-3,6-dioxocyclohexa-1,4-diene-1-carboxylate (34)

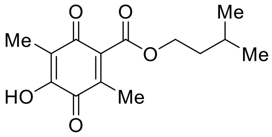

Following the general protocol on 0.32 mmol scale (**20**), benzochinone **34** was obtained as an orange solid (13.0 mg, 50.0 µmol, 15%).

TLC: R_f (cyclohexane/EtOAc = 10:1 + 1% AcOH) = 0.19. **^{1}H-NMR** (300 MHz, $CDCl_3$): δ = 6.93 (bs, 1 H, O*H*), 4.36 (t, *J* = 6.8 Hz, 2 H, $OCH_2CH_2CH(CH_3)_2$), 2.05 (s, 3 H, CH_3), 1.95 (s, 3 H, CH_3), 1.80–1.65 (m, 1 H, $OCH_2CH_2CH(CH_3)_2$), 1.61 (q , *J* = 6.8 Hz, 2 H, $OCH_2CH_2CH(CH_3)_2$), 0.94 (d, *J* = 6.5 Hz, 6 H, $OCH_2CH_2CH(CH_3)_2$). **^{13}C-NMR** (75 MHz, $CDCl_3$): δ = 184.4, 183.5, 164.4, 151.2, 138.9, 137.2, 117.3, 65.0, 37.3, 25.1, 22.5, 12.6, 8.1. **HRMS** (ESI+) *m/z* calcd for $C_{14}H_{18}O_5Na$ $[M+Na]^+$: 289.1046; found: 289.1051.

Hexyl 4-hydroxy-2,5-dimethyl-3,6-dioxocyclohexa-1,4-diene-1-carboxylate (35)

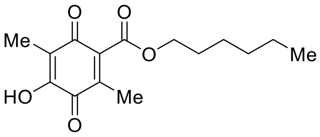

Following the general protocol on 0.30 mmol scale (**21**), benzochinone **35** was obtained as an orange oil (10.0 mg, 40.0 µmol, 12%).

TLC: R_f (cyclohexane/EtOAc = 10:1 + 1% AcOH) = 0.17. **^{1}H-NMR** (600 MHz, $CDCl_3$): δ = 6.91 (bs, 1 H, O*H*), 4.32 (t, *J* = 6.8 Hz, 2 H, $OCH_2CH_2CH_2CH_2CH_2CH_3$), 2.05 (s, 3 H, CH_3), 1.95 (s, 3 H, CH_3), 1.72 (p, *J* = 6.8 Hz, 2 H, $OCH_2CH_2CH_2CH_2CH_2CH_3$), 1.43–1.36 (m, 2 H, $OCH_2CH_2CH_2CH_2CH_2CH_3$), 1.35–1.28 (m, 4 H, $OCH_2CH_2CH_2CH_2CH_2CH_3$), 0.89 (t, *J* = 7.1 Hz, 3 H, $OCH_2CH_2CH_2CH_2CH_2CH_3$). **^{13}C-NMR** (151 MHz, $CDCl_3$): δ = 184.4, 183.5, 164.4, 151.2, 137.3, 117.4, 66.4, 31.5, 28.6, 25.6,

22.6, 14.1, 12.6, 8.1. **HRMS** (ESI+) *m/z* calcd for $C_{15}H_{20}O_5Na$ $[M+Na]^+$: 303.1203; found: 303.1206.

Isohexyl 4-hydroxy-2,5-dimethyl-3,6-dioxocyclohexa-1,4-diene-1-carboxylate (36)

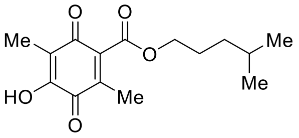

Following the general protocol on 0.30 mmol scale (**22**), benzochinone **36** was obtained as an orange oil (15.0 mg, 50.0 µmol, 18%).

TLC: R_f (cyclohexane/EtOAc = 10:1 + 1% AcOH) = 0.13. **^{1}H-NMR** (300 MHz, $CDCl_3$): δ = 6.91 (bs, 1 H, O*H*), 4.31 (t, *J* = 6.8 Hz, 2 H, OC$H_2$$CH_2CH_2CH(CH_3)_2$), 2.06 (s, 3 H, C$H_3$), 1.95 (s, 3 H, C$H_3$), 1.80–1.65 (m, 2 H, OCH_2C$H_2$$CH_2CH(CH_3)_2$), 1.58 (h, *J* = 6.7 Hz, 1 H, $OCH_2CH_2CH_2$C*H*$(CH_3)_2$), 1.33–1.16 (m, 2 H, OCH_2CH_2C$H_2$$CH(CH_3)_2$), 0.90 (d, *J* = 6.6 Hz, 6 H, $OCH_2CH_2CH_2CH$(CH_3)$_2$).
^{13}C-NMR (75 MHz, $CDCl_3$): δ = 184.4, 183.5, 164.4, 151.2, 138.9, 137.3, 117.4, 66.7, 35.0, 27.8, 26.6, 22.6, 12.6, 8.1. **HRMS** (ESI+) *m/z* calcd for $C_{15}H_{20}O_5Na$ $[M+Na]^+$: 303.1203; found: 303.1206.

But-3-en-1-yl 4-hydroxy-2,5-dimethyl-3,6-dioxocyclohexa-1,4-diene-1-carboxylate (37)

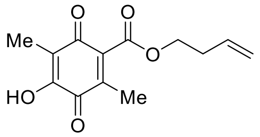

Following the general protocol on 0.42 mmol scale (**23**), benzochinone **37** was obtained as an orange oil (10.0 mg, 40.0 µmol, 10%).

TLC: R_f (*n*-pentane/EtOAc = 6:1) = 0.31. **^{1}H-NMR** (300 MHz, $CDCl_3$): δ = 6.91 (bs, 1 H, O*H*), 5.81 (ddt, *J* = 17.0, 10.2, 6.6 Hz, 1 H, OCH_2CH_2C*H*$=CH_2$), 5.26–5.04 (m, 2 H, $OCH_2CH_2CH=$CH_2), 4.39 (t, *J* = 6.7 Hz, 2 H, OC$H_2$$CH_2CH=CH_2$), 2.49 (qt, *J* = 6.7, 1.4 Hz, 2 H, OCH_2C$H_2$$CH=CH_2$), 2.05 (s, 3 H, C$H_3$), 1.95 (s, 3 H, C$H_3$). **^{13}C-NMR** (75 MHz, $CDCl_3$): δ = 184.3, 183.4, 164.2, 151.2, 138.7, 137.4, 133.6, 117.9, 117.4, 65.2, 33.0, 12.7, 8.1. **HRMS** (ESI+) *m/z* calcd for $C_{13}H_{14}O_5Na$ $[M+Na]^+$: 273.0733; found: 273.0729.

But-3-in-1-yl 4-hydroxy-2,5-dimethyl-3,6-dioxocyclohexa-1,4-diene-1-carboxylate (38)

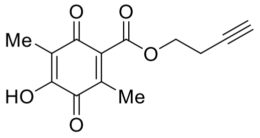

Following the general protocol on 0.30 mmol scale (**24**), benzochinone **38** was obtained as an orange solid (10.0 mg, 40.0 µmol, 14%).

TLC: R_f (*n*-pentane/EtOAc = 5:1 + 1% AcOH) = 0.34. **^{1}H-NMR** (300 MHz, $CDCl_3$): δ = 6.92 (bs, 1 H, O*H*), 4.44 (t, *J* = 6.7 Hz, 2 H, OC$H_2$$CH_2CCH$), 2.64 (td, *J* = 6.7, 2.7 Hz, 2 H, OCH_2CH_2CCH),

2.09 (s, 3 H, CH_3), 2.02 (t, *J* = 2.7 Hz, 1 H, OCH_2CH_2CCH), 1.95 (s, 3 H, CH_3). **^{13}C-NMR** (75 MHz, $CDCl_3$): δ = 184.2, 183.3, 163.9, 151.3, 138.4, 117.4, 79.7, 70.5, 63.7, 19.1, 12.7, 8.1. **HRMS** (ESI+) *m/z* calcd for $C_{13}H_{12}O_5Na$ $[M+Na]^+$: 271.0577; found: 271.0578.

Pent-4-en-1-yl 4-hydroxy-2,5-dimethyl-3,6-dioxocyclohexa-1,4-diene-1-carboxylate (39)

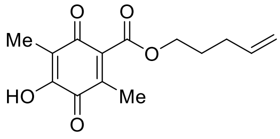

Following the general protocol on 0.32 mmol scale (**25**), benzochinone **39** was obtained as an orange solid (12.0 mg, 40.0 µmol, 14%).

TLC: R_f (cyclohexane/EtOAc = 10:1 + 1% AcOH) = 0.13. **^{1}H-NMR** (300 MHz, $CDCl_3$): δ = 6.92 (bs, 1 H, O*H*), 5.81 (ddt, *J* = 16.9, 10.2, 6.6 Hz, 1 H, OCH_2CH_2C*H*$=CH_2$), 5.13–4.95 (m, 2 H, $OCH_2CH_2CH=C$*H_2*), 4.34 (t, *J* = 6.6 Hz, 2 H, OC*H_2*$CH_2CH_2CH=CH_2$), 2.23–2.10 (m, 2 H, OCH_2CH_2C*H_2*$CH=CH_2$), 2.06 (s, 3 H, CH_3), 1.95 (s, 3 H, CH_3), 1.89–1.77 (m, 2 H, OCH_2C*H_2*$CH_2CH=CH_2$). **^{13}C-NMR** (75 MHz, $CDCl_3$): δ = 184.3, 183.5, 164.3, 151.2, 138.8, 137.3, 137.2, 117.4, 115.8, 65.6, 30.0, 27.8, 12.6, 8.1. **HRMS** (ESI+) *m/z* calcd for $C_{14}H_{16}O_5Na$ $[M+Na]^+$: 287.0890; found: 287.0892.

Pent-4-in-1-yl 4-hydroxy-2,5-dimethyl-3,6-dioxocyclohexa-1,4-diene-1-carboxylate (40)

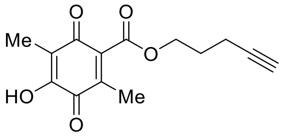

Following the general protocol on 0.25 mmol scale (**26**), benzochinone **40** was obtained as an orange solid (7.00 mg, 30.0 µmol, 11%).

TLC: R_f (*n*-pentane/EtOAc = 10:1 + 1% AcOH) = 0.26. **^{1}H-NMR** (300 MHz, $CDCl_3$): δ = 6.91 (bs, 1 H, O*H*), 4.45 (t, *J* = 6.3 Hz, 2 H, OC*H_2*CH_2CH_2CCH), 2.35 (td, *J* = 7.0, 2.6 Hz, 2 H, OCH_2CH_2C*H_2*CCH), 2.06 (s, 3 H, CH_3), 2.01–1.90 (m, 3 H, OCH_2C*H_2*CH_2CC*H*, CH_3). **^{13}C-NMR** (75 MHz, $CDCl_3$): δ = 184.3, 183.4, 164.2, 151.3, 138.7, 137.5, 117.4, 82.8, 69.5, 64.6, 27.5, 15.2, 12.6, 8.1. **HRMS** (ESI+) *m/z* calcd for $C_{14}H_{14}O_5Na$ $[M+Na]^+$: 285.0733; found: 285.0734.

Benzyl 4-hydroxy-2,5-dimethyl-3,6-dioxocyclohexa-1,4-diene-1-carboxylate (41)

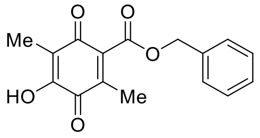

Following the general protocol on 0.29 mmol scale (**27**), benzochinone **41** was obtained as an orange solid (12.0 mg, 40.0 µmol, 14%).

TLC: R_f (cyclohexane/EtOAc = 10:1 + 1% AcOH) = 0.09. **^{1}H-NMR** (300 MHz, $CDCl_3$): δ = 7.44–7.34 (m, 5 H, Ar*H*), 6.90 (bs, 1 H, O*H*), 5.36 (s, 2 H, OC*H_2*), 2.00 (s, 3 H, CH_3), 1.95 (s, 3 H, CH_3).

^{13}C-NMR (75 MHz, $CDCl_3$): δ = 184.2, 183.4, 164.1, 151.2, 138.5, 137.7, 135.0, 128.9, 128.8, 128.7, 117.4, 68.0, 12.5, 8.1. **HRMS** (ESI+) *m/z* calcd for $C_{16}H_{14}O_5Na$ $[M+Na]^+$: 309.0733; found: 309.0734.

2.5 Enzymatic Synthesis of Sorrentanone Analogs (28-41)

The enzymatic evaluation of the sorrentanone substrates was performed as described previously.[7] Each analytical assay contained 706 µL of 50 mM phosphate buffer (pH 8.0), 150 µL acetone, 20 µL substrate stock solution (100 mM, 2.00 µmol, 1.00 equiv.), 80 µL NADH stock solution (50 mM, 4.00 µmol, 2.00 equiv.) and 44.0 µL SorbC solution (11.0 mg/mL, 10.0 nmol, 0.5 mol%) with a final volume of 1 mL. The reaction was incubated at 25 °C and gently shaken at 250 rpm using a Benchmark Multi-ThermTM Heat - Shake. HPLC analysis was performed by taking a 100 µL sample after 30, 60 and 90 min. This sample was diluted with 100 µL MeCN and precipitated enzyme was centrifuged off (14000 rpm, 2 min, Eppendorf Centrifuge 5415 D). The supernatant was transferred to an HPLC vial and 20 µL was injected into the HPLC. Substrate consumption was calculated by integration of the peak area and comparison with the corresponding calibration curve.

3. Bioactivity and Toxicity Assays

Antimicrobial assay: According to the standard broth micro-dilution assays, following the Standards of European Committee on Antimicrobial Susceptibility Testing (v 7.3.1: Method for the determination of broth dilution minimum inhibitory concentrations of antifungal agents for yeasts) for *Candida* spp., and according to the standard broth micro-dilution assays, recommended by the National Committee for Clinical Laboratory Standards (M07-A8) for bacteria MIC values of compounds were determinate. Compounds were dissolved in DMSO at a concentration of 50 mg/mL, and the highest tested concentration was 250 µg/mL. The inoculums were $1x10^5$ colony forming units (CFU/mL), for *Candida* species, and bacteria species, $5x10^5$ colony forming units (CFU/mL). The MIC value was recorded as the lowest concentration that inhibited the growth after 24 h at 37 °C. Additionally, 5 µL from each well of 96 well plates was transferred to Luria Agar (LA) plates for bacteria and Sabouraud agar (SAB) plates for *Candida* spp. and incubated for an additional 24 h at 37 °C, to determine MBC (Minimum bactericidal/fungicidal concentration) values. In Table 1. Are labeled compounds showing a difference in MBC and previously determined MIC values.

Cytotoxicity evaluation: Antiproliferative activity was tested by MTT assay (3-(4,5-dimethylthiazol-2-yl)-2,5-diphenyltetrazoliumbromide) on a human lung fibroblast cell line (MRC-5; ATCC collection). Pre-grown (24 h) cell monolayers ($1x10^4$ cells per well) in RPMI 1640 medium supplemented with 100 µg/mL streptomycin, 100 U/mL penicillin, and 10% (*v*/*v*) FBS (all from Sigma, Munich, Germany) containing the tested compounds at concentrations ranging from 5 to 20 µg/mL were incubated in a humidified atmosphere of 95% air and 5% CO_2 at 37 °C for 48 h. The cell viability (extent of MTT reduction) was measured spectrophotometrically at 540 nm using the Epoch 2000 multiplate reader, and the cell survival was expressed as a percentage of the control (untreated cells). Cytotoxicity was expressed as the concentration of the compound inhibiting cell growth by 50% (IC_{50}).

In vivo toxicity C. elegans assay: The *C. elegans* survival assay was carried out as described previously with some modifications.[8] In brief, synchronized worms (L4 stage) were suspended in a medium containing 95% M9 buffer (3.0 g of KH_2PO_4, 6.0 g of Na_2HPO_4, 5.0 g of NaCl, and 1 mL of 1 M $MgSO_4 \times 7H_2O$ in 1 L of water), 5% LB (Luria-Bertani) broth (Oxoid, Basingstoke, UK), and 10 µg of cholesterol (Sigma-Aldrich, Munich, Germany) per mL. The experiment was carried out in 96-well flat-bottomed microtiter plates (Sarstedt, Nümbrecht, Germany) in the final volume of 100 µL per well. 25 µL of this suspension of nematodes (25–35 nematodes) were transferred to the wells of a 96-well microtiter plate, where 50 µL of the medium was previously added. Next, 25 µL of a solvent control (DMSO) or 25 µL of a concentrated solution was added to the test wells. The final concentrations of the compounds were 50, 25, 10, and 5 µg/mL. Subsequently, the plates were incubated at 25 °C for 2 days. The fraction of dead worms was determined after 48 h by counting the number of dead worms and the total number of worms in each well, using a stereomicroscope (SMZ143-N2GG, Motic, Germany).

As a negative control experiment, nematodes were exposed to the medium containing 1% DMSO.

Filamentation test on Candida: Using solid composition, the effect of sorrentanone derivatives on *C. albicans* ATCC 10231 hyphae formation was assessed. Briefly, 1 mL of *C. albicans* cells from an overnight culture grown in Sabouraud dextrose broth medium at 37 °C with or without compounds were centrifuged for 10 minutes at maximum speed, and the pellet was washed several times with sterile PBS (phosphate-buffered saline). After it, the pellet was resuspended in 200 µL PBS, and 2 µL of cell suspension was poured over solid composition and incubated at 37 °C for 120 h. Morphological changes of *C. albicans* ATCC 10231 in the presence of subinhibitory concentration (0.8 and 0.5 × MIC value) compounds or without them were analyzed using bright field microscopy (ZEISS Optical microscope, 1.6x magnification). *C. albicans* ATCC 10231 treated with DMSO was used as a control.

Anti-biofilm activity assessment: The effect of compounds on biofilms formation was determined on *C. albicans* ATCC 10231 strain. Anti-biofilm assays were conducted using a previously reported methodology with small modifications.[9] The assay was carried out in 96-well round-bottom polystyrene microtiter plates. Cells were harvested from overnight grown cultures, washed twice with sterile phosphate-buffered saline (PBS; Sigma-Aldrich, Munich, Germany), and resuspended in RPMI 1640 medium (Sigma-Aldrich) containing 2% glucose (w/v) at a concentration of 1×10^5 CFU/mL. *C. albicans* suspension was incubated with 0.5 MIC, 1 MIC, and 2 MIC compounds concentration in 200.0 µL final volume per well for 48 h at 37 °C to allow biofilm formation. Biofilm growth was analyzed by crystal violet (CV) staining of adherent cells and the absorbance at 590.0 nm was read on a Tecan Infinite 200 Pro multiplate reader (Tecan Group Ltd., Männedorf, Switzerland).

Ergosterol biosynthesis assay: Ergosterol levels were measured according to the published procedure with some minor modifications.[10] Briefly, a single *C. albicans* colony from an overnight Sabouraud dextrose agar plate was used to inoculate 50 ml of Sabouraud dextrose broth containing 0.5 MIC concentrated compounds. Incubation was performed for 18 h at 37 °C on the rotary shaker at 180 rpm. Cells were harvested by centrifugation at 3000 rpm for 5 min and washed once with sterile distilled water. The net wet weight of the cell pellet was determined. Three milliliters of 25% alcoholic potassium hydroxide solution (25 g of KOH and 35 ml of sterile distilled water, brought to 100 ml with 100% ethanol), was added to each pellet and vortex mixed for 1 min and incubated in an 85 °C water bath for 1 h. Tubes were cooled to room temperature and extraction of sterols was done with the addition of a mixture of 1 ml of sterile distilled water and 3 ml of *n*-heptane followed by vigorous vortex mixing for 3 min. The heptane layer was used for spectrophotometrically measuring ergosterol concentrations by scanning absorbance between 240 nm and 300 nm using Ultrospec 3300pro 573 (Amersham Biosciences, Amersham, UK).

4. NMR Data

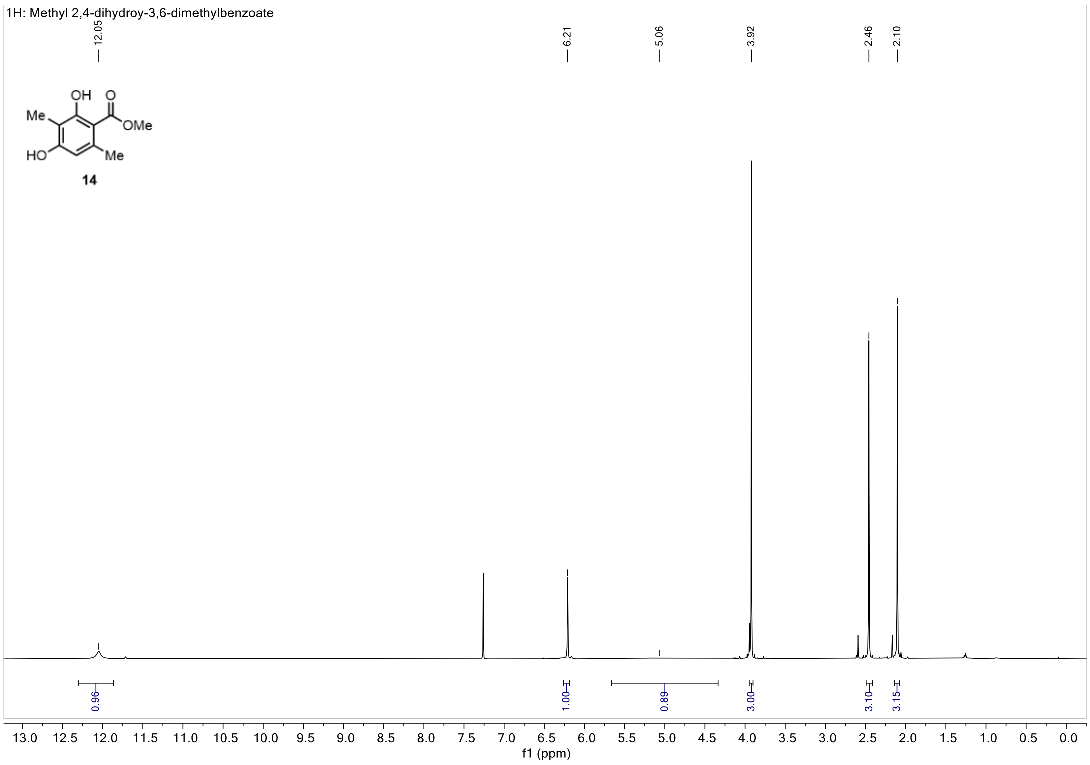

Fig. S1 ^{1}H-NMR spectrum of methyl 2,4-dihydroy-3,6-dimethylbenzoate (**14**), measured in $CDCl_3$ at 300 MHz.

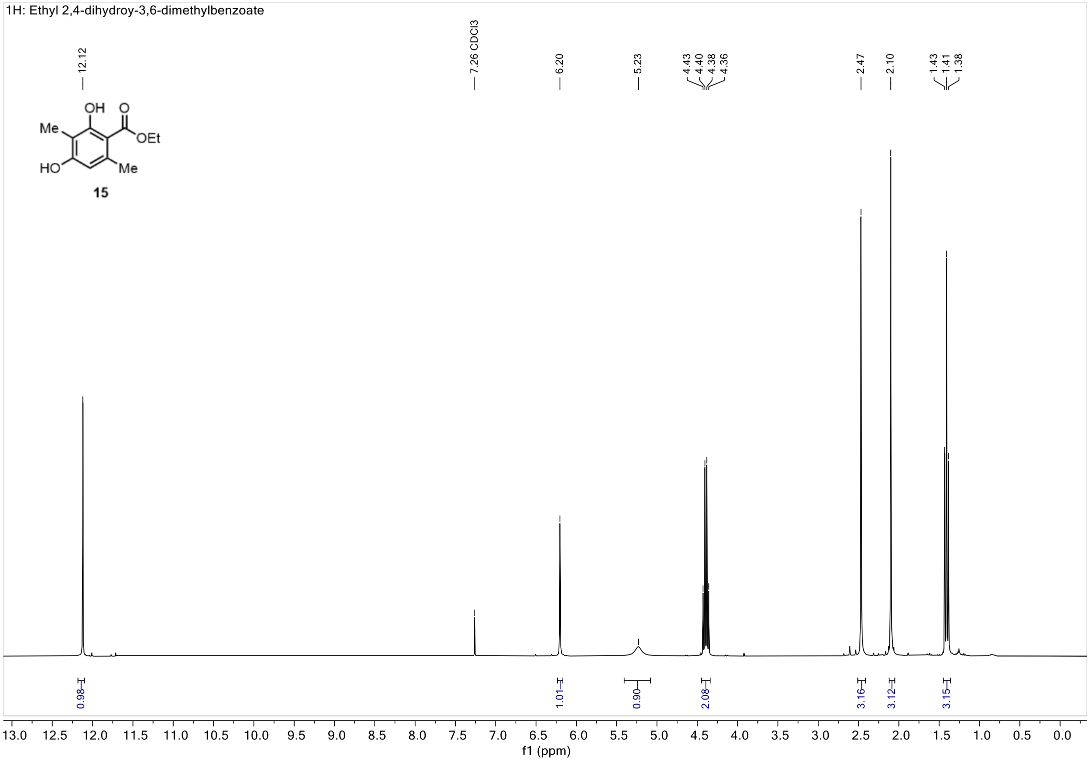

Fig. S2 ^{1}H-NMR spectrum of ethyl 2,4-dihydroy-3,6-dimethylbenzoate (**15**), measured in $CDCl_3$ at 300 MHz.

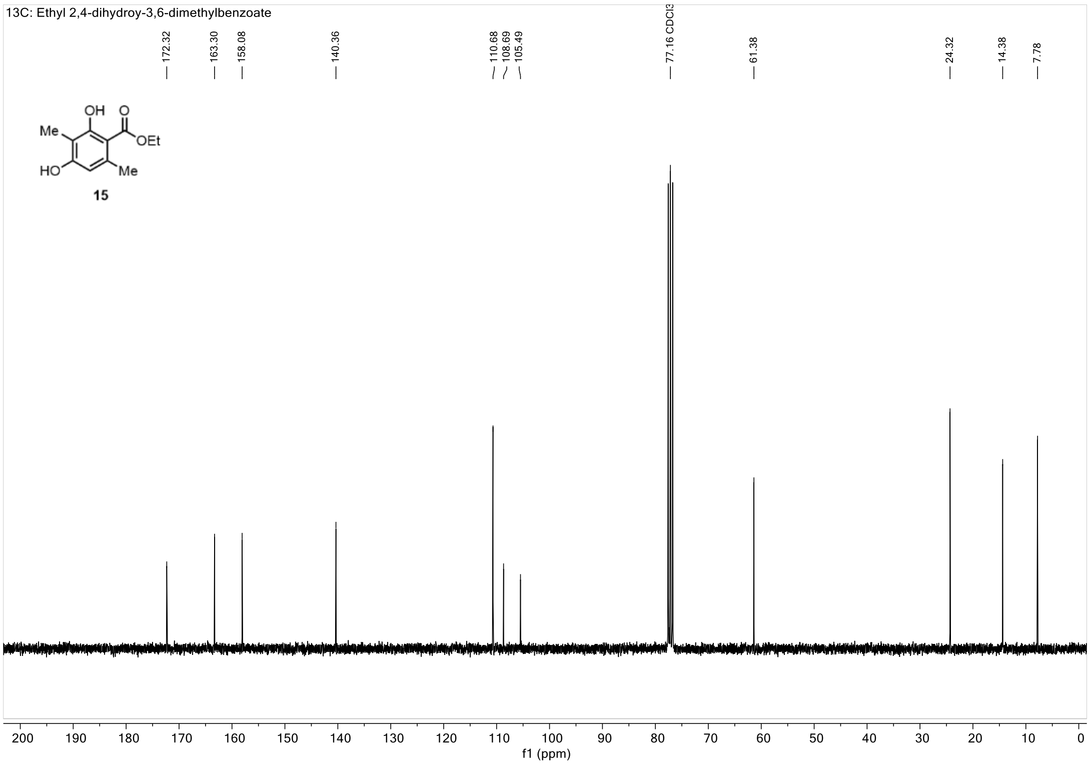

Fig. S3 ^{13}C-NMR spectrum of ethyl 2,4-dihydroy-3,6-dimethylbenzoate (**XX**15 measured in $CDCl_3$ at 75 MHz.

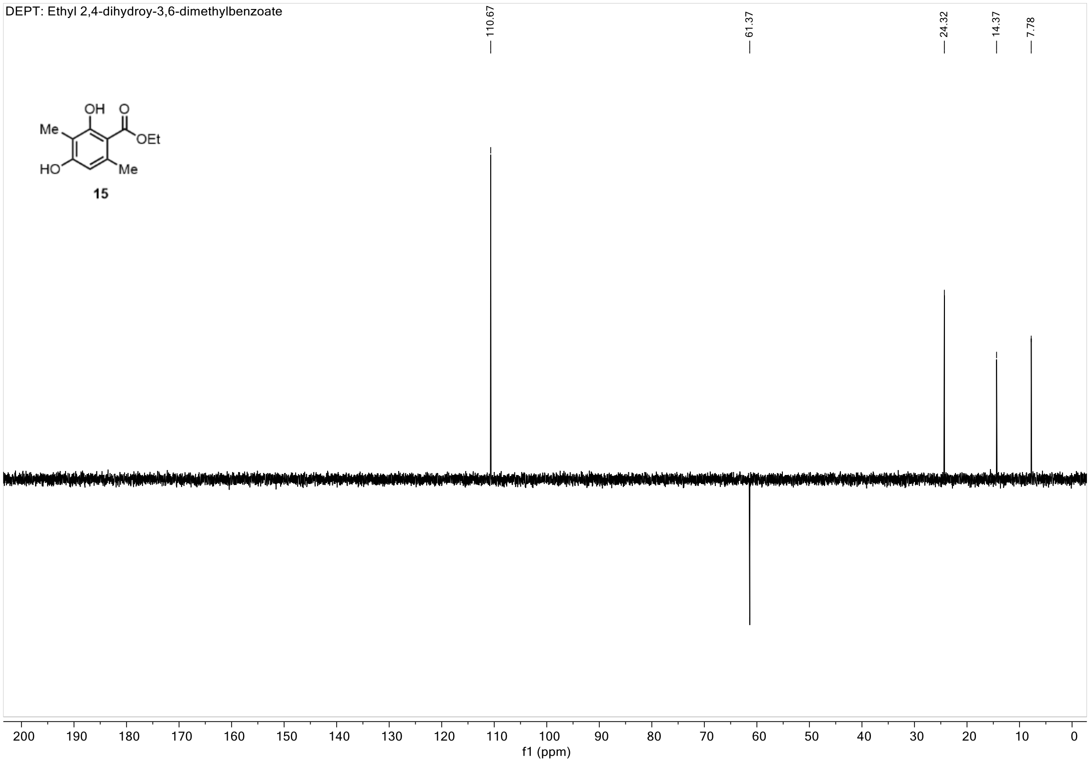

Fig. S4 DEPT spectrum of ethyl 2,4-dihydroy-3,6-dimethylbenzoate (**15**), measured in $CDCl_3$ at 75 MHz.

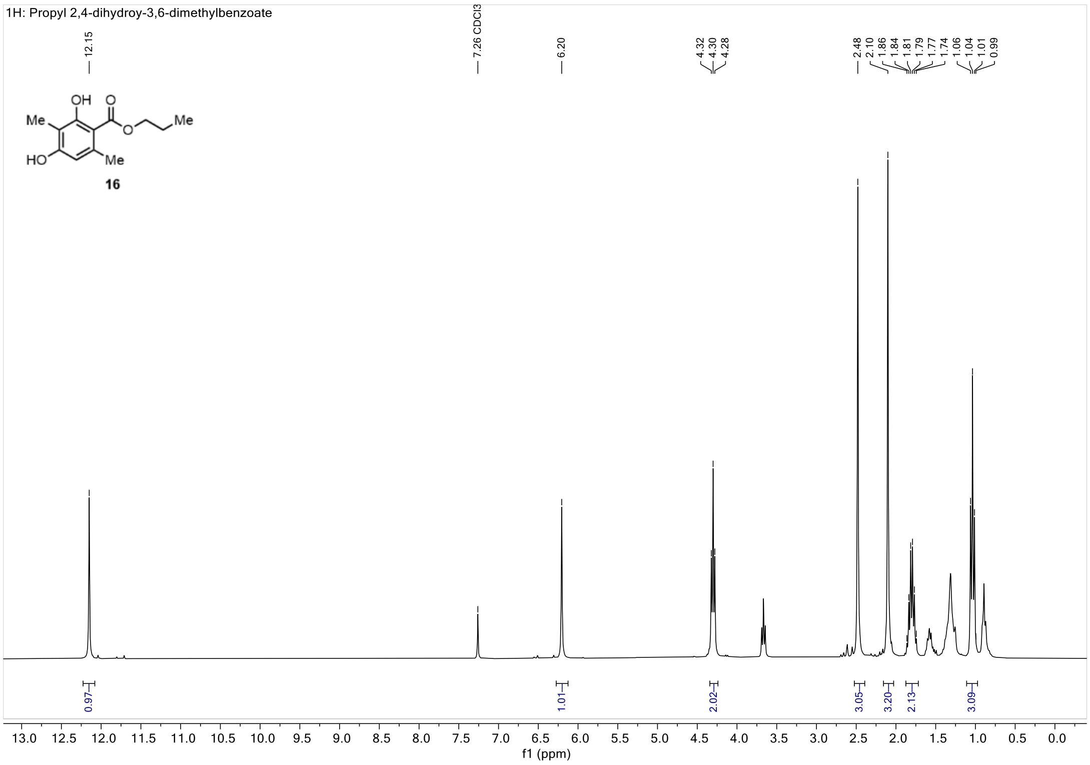

Fig. S5 ^{1}H-NMR spectrum of propyl 2,4-dihydroy-3,6-dimethylbenzoate (**16**), measured in $CDCl_3$ at 300 MHz.

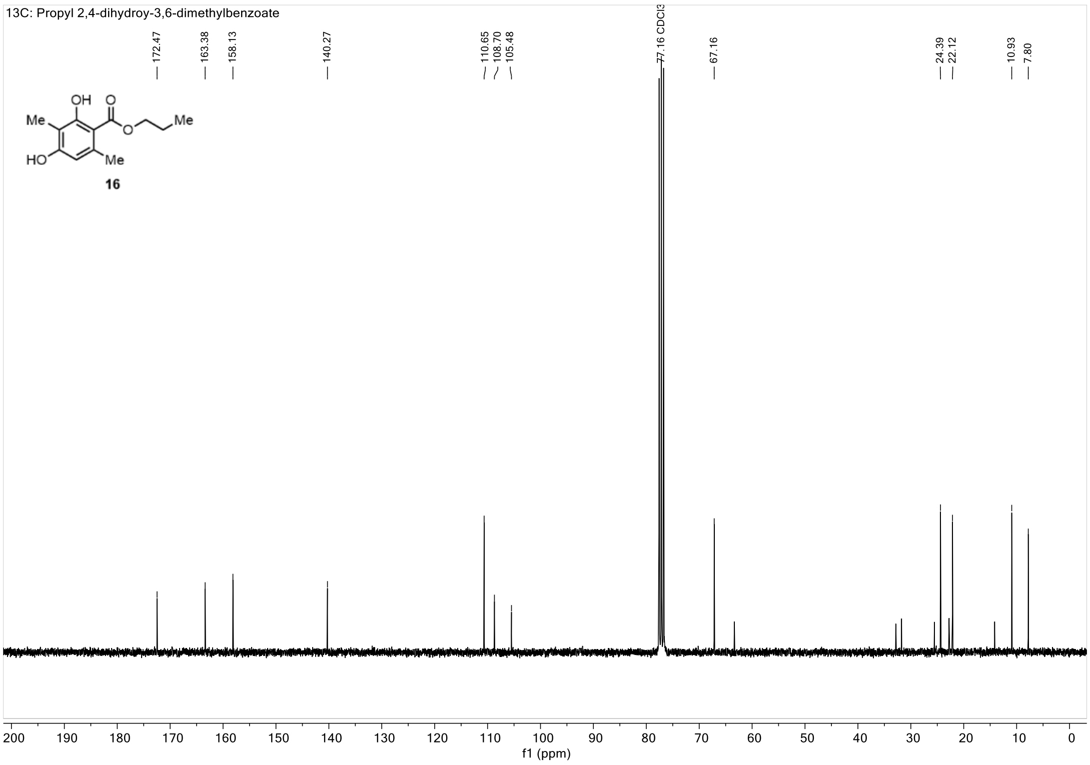

Fig. S6 ^{13}C-NMR spectrum of propyl 2,4-dihydroy-3,6-dimethylbenzoate (**16**), measured in $CDCl_3$ at 75 MHz.

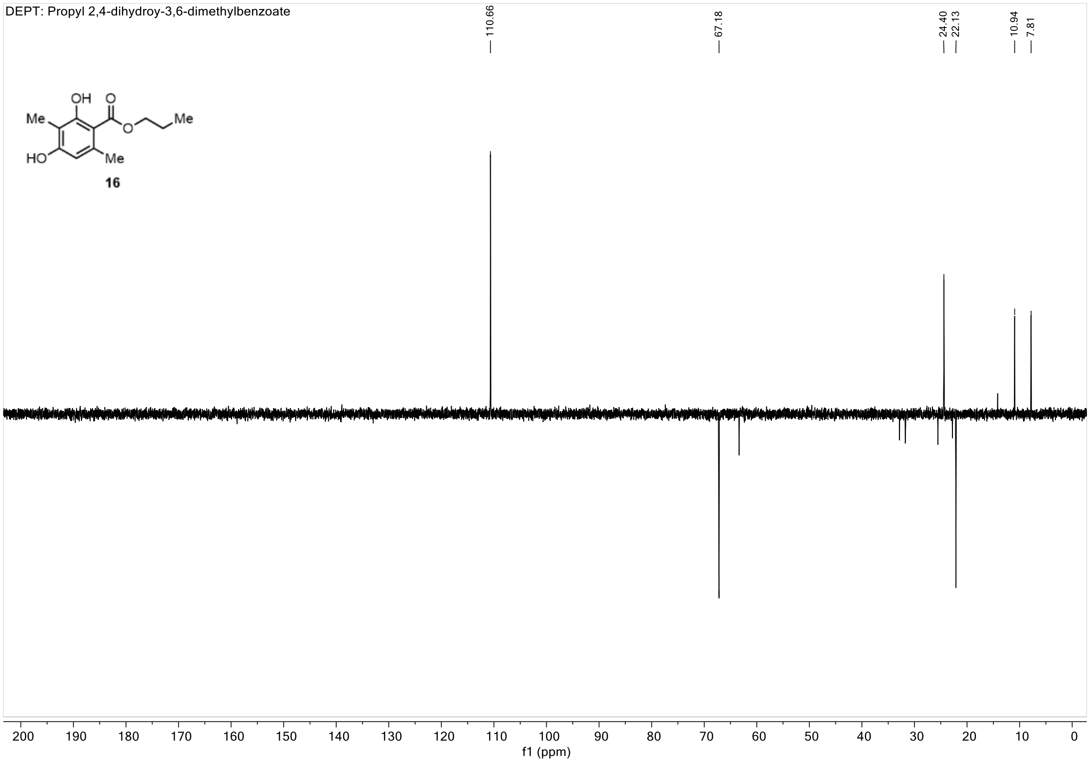

Fig. S7 DEPT spectrum of propyl 2,4-dihydroy-3,6-dimethylbenzoate (**16**), measured in $CDCl_3$ at 75 MHz.

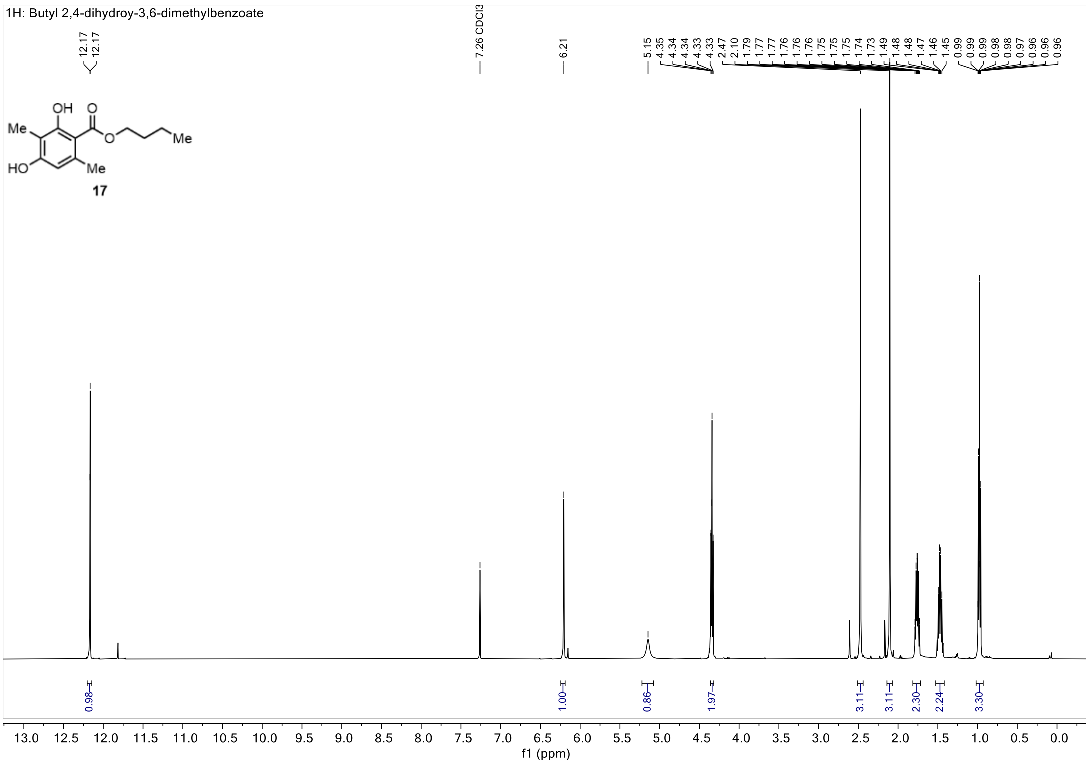

Fig. S8 ^{1}H-NMR spectrum of butyl 2,4-dihydroy-3,6-dimethylbenzoate (**17**), measured in $CDCl_3$ at 300 MHz.

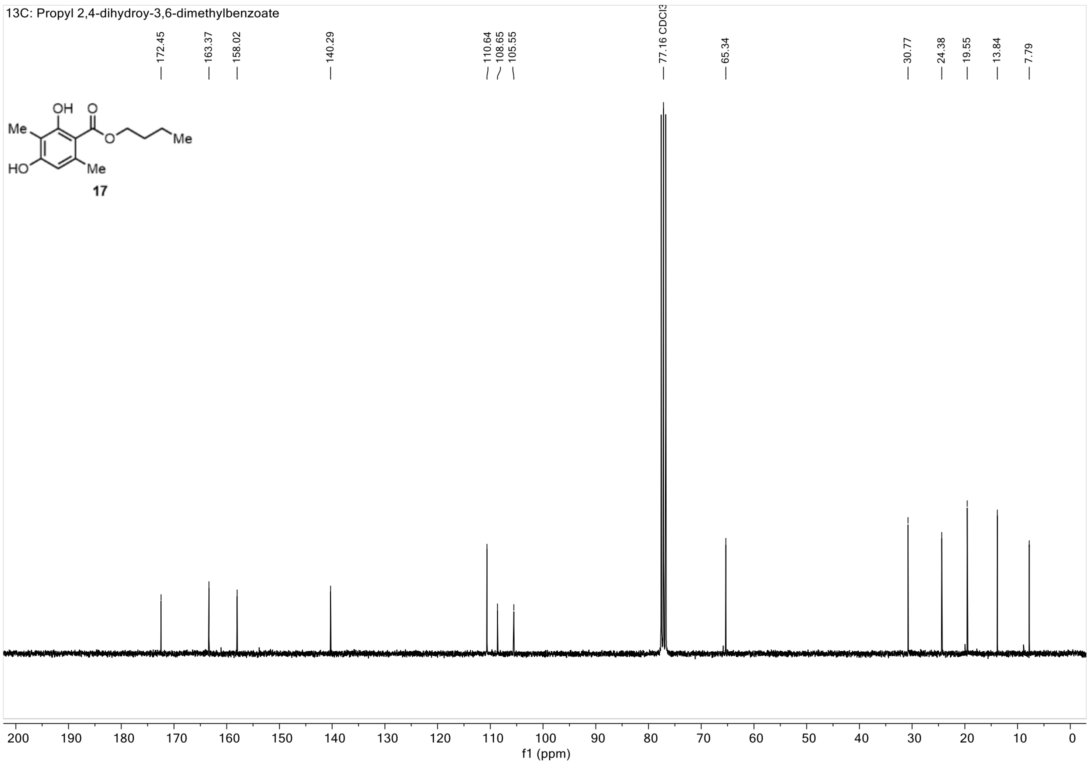

Fig. S9 ^{13}C-NMR spectrum of butyl 2,4-dihydroy-3,6-dimethylbenzoate (**17**), measured in $CDCl_3$ at 75 MHz.

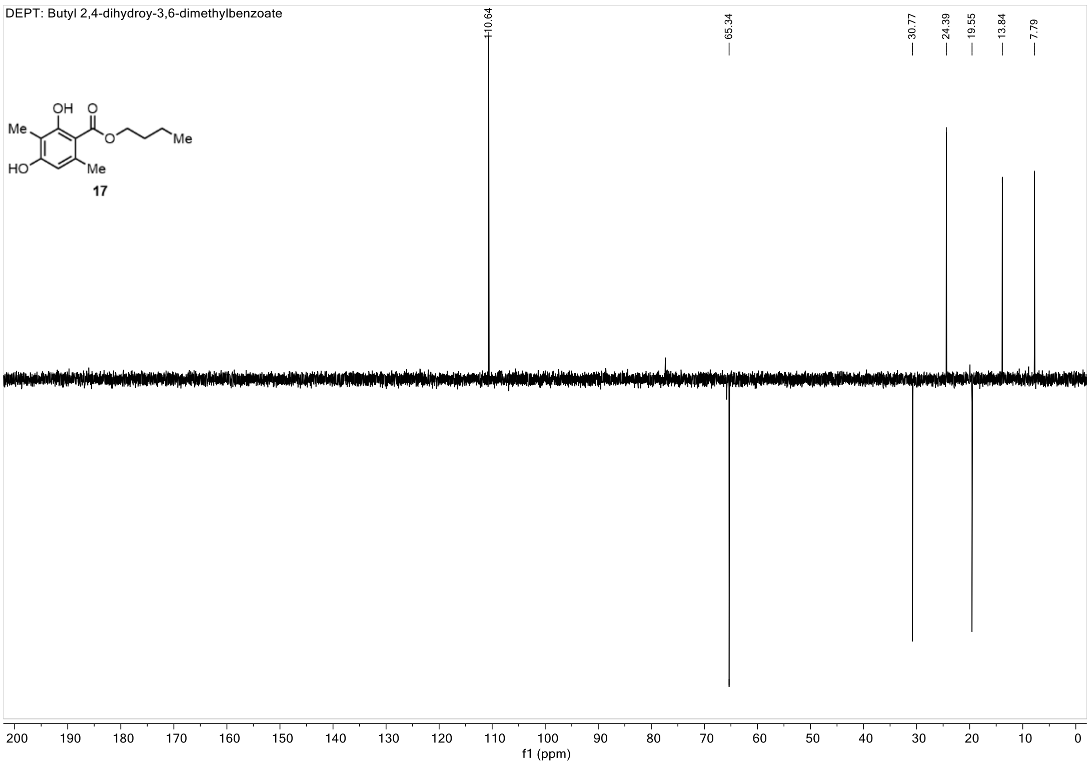

Fig. S10 DEPT spectrum of butyl 2,4-dihydroy-3,6-dimethylbenzoate (**17**), measured in $CDCl_3$ at 75 MHz.

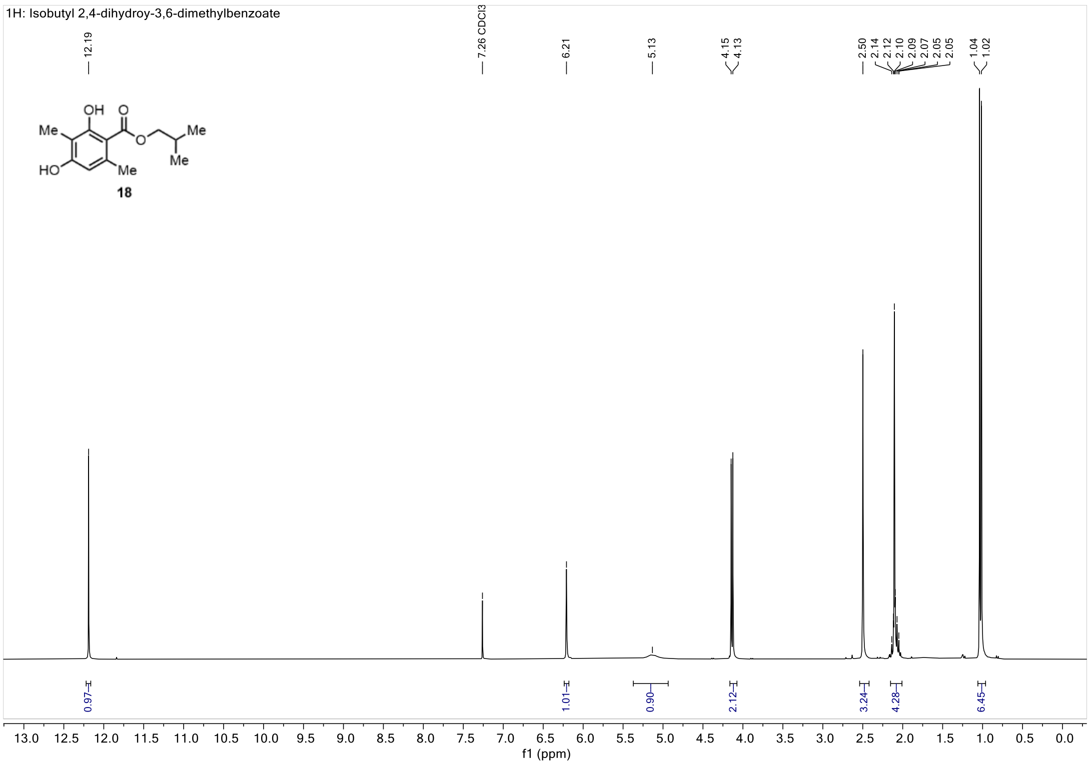

Fig. S11 ^{1}H-NMR spectrum of isobutyl 2,4-dihydroy-3,6-dimethylbenzoate (**18**), measured in $CDCl_3$ at 300 MHz.

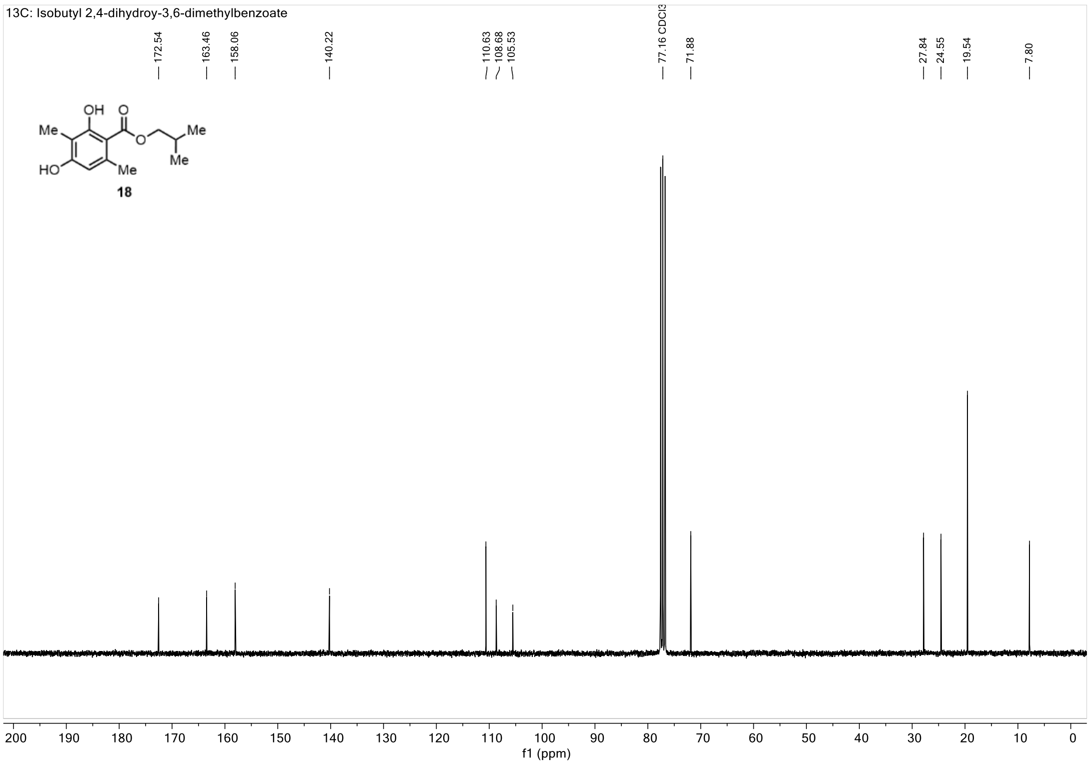

Fig. S12 ^{13}C-NMR spectrum of isobutyl 2,4-dihydroy-3,6-dimethylbenzoate (**18**), measured in $CDCl_3$ at 75 MHz.

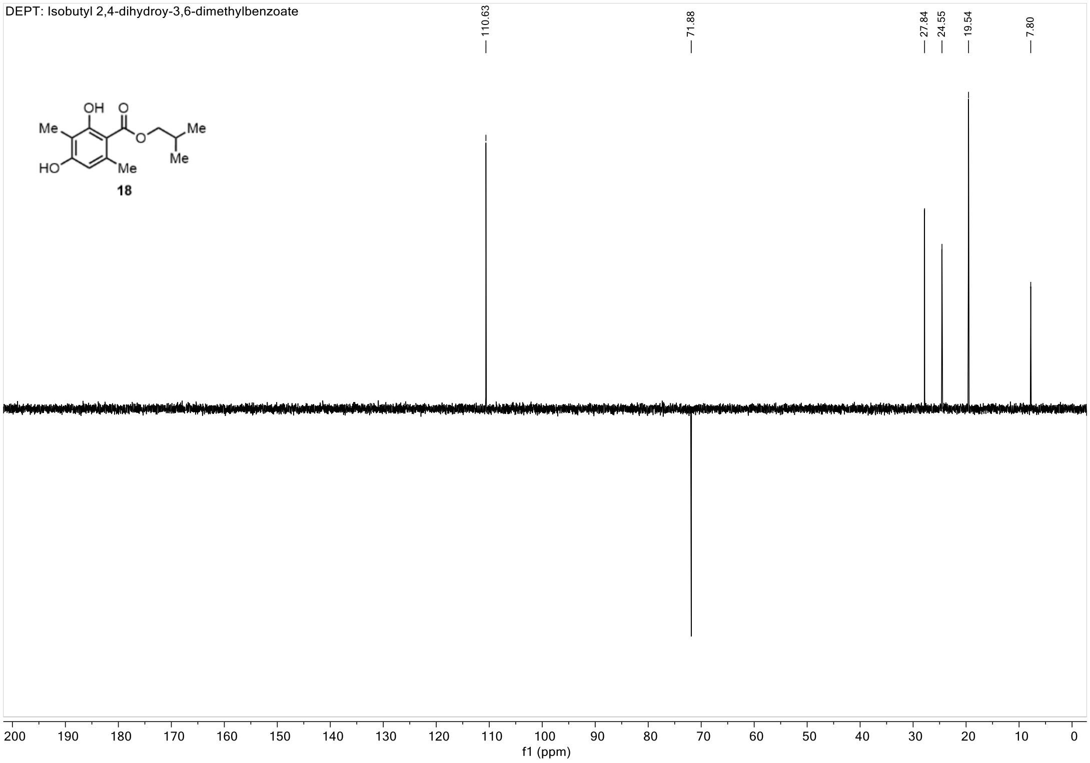

Fig. S13 DEPT spectrum of isobutyl 2,4-dihydroy-3,6-dimethylbenzoate (**18**), measured in $CDCl_3$ at 75 MHz.

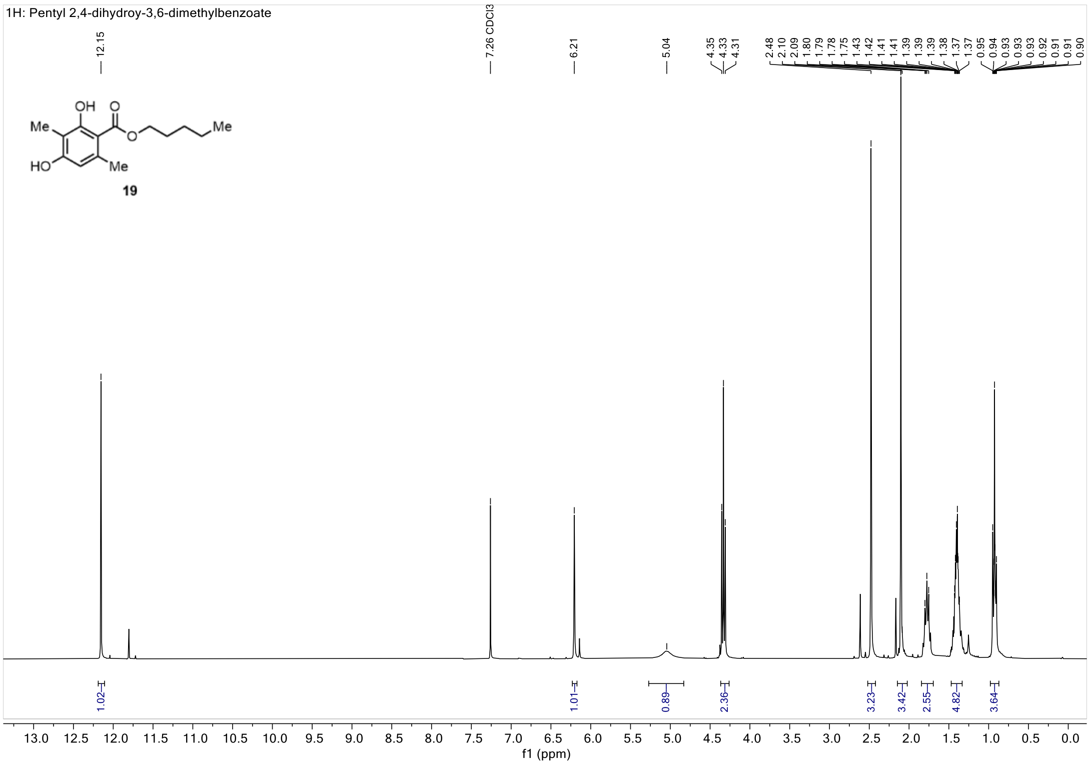

Fig. S14 ^{1}H-NMR spectrum of pentyl 2,4-dihydroy-3,6-dimethylbenzoate (**19**), measured in $CDCl_3$ at 300 MHz.

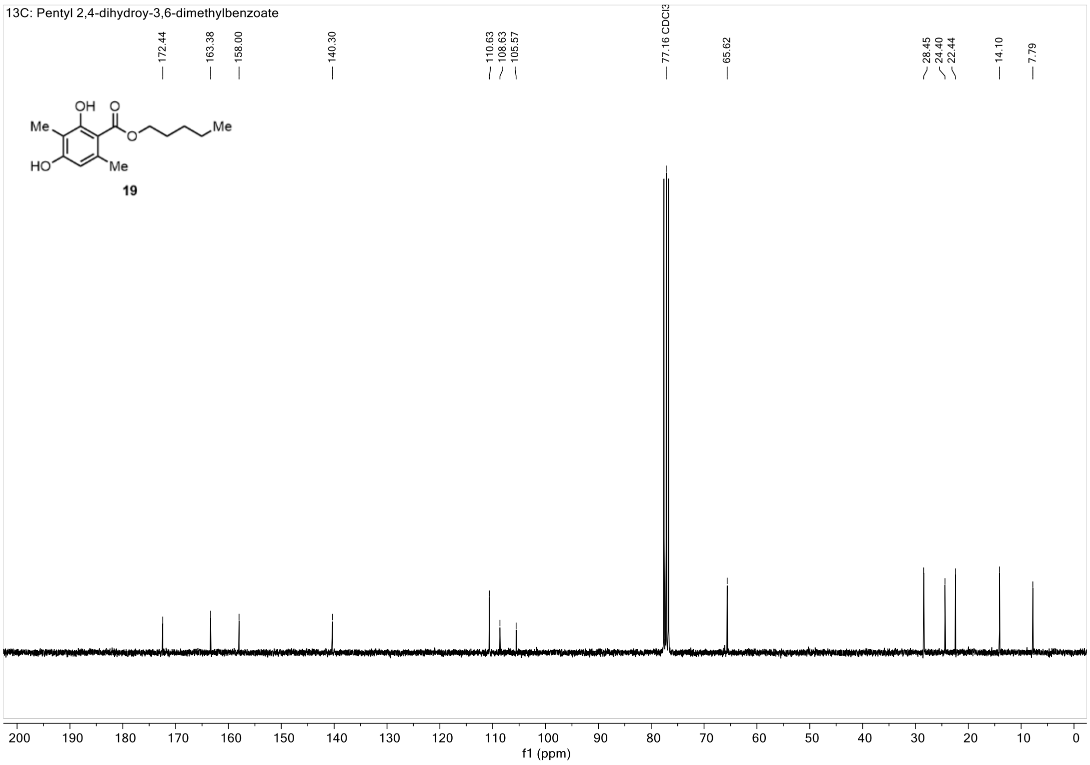

Fig. S15 ^{13}C-NMR spectrum of pentyl 2,4-dihydroy-3,6-dimethylbenzoate (**19**), measured in $CDCl_3$ at 75 MHz.

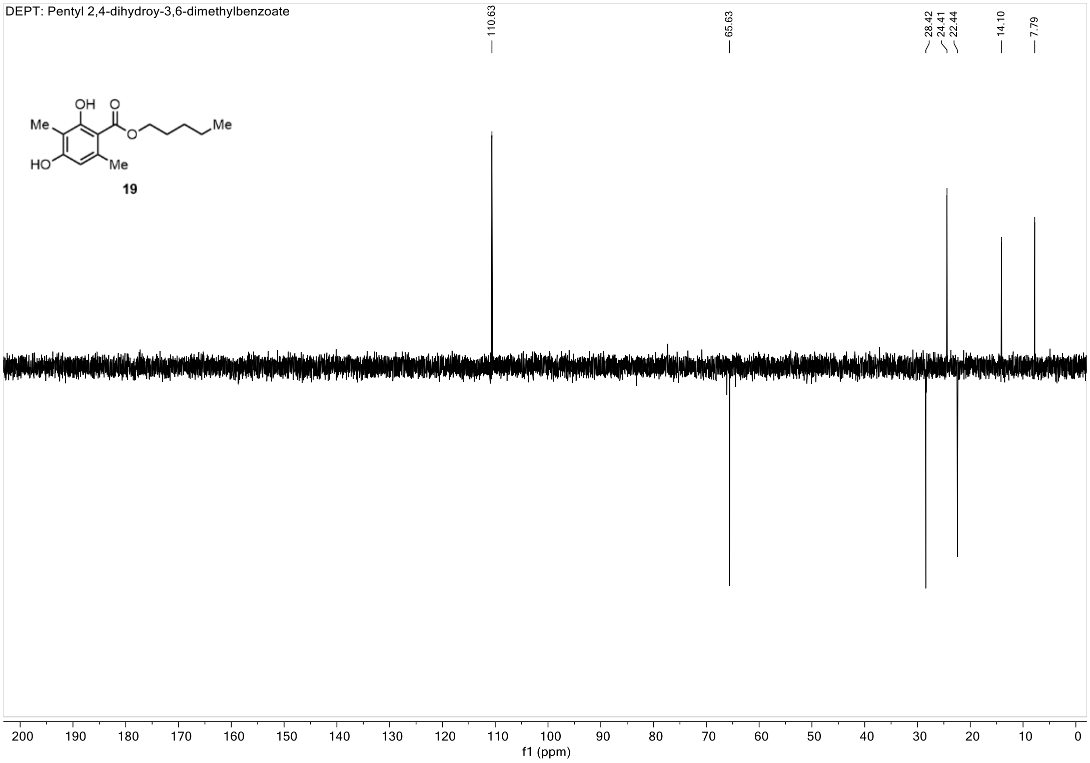

Fig. S16 DEPT spectrum of pentyl 2,4-dihydroy-3,6-dimethylbenzoate (**19**), measured in $CDCl_3$ at 75 MHz.

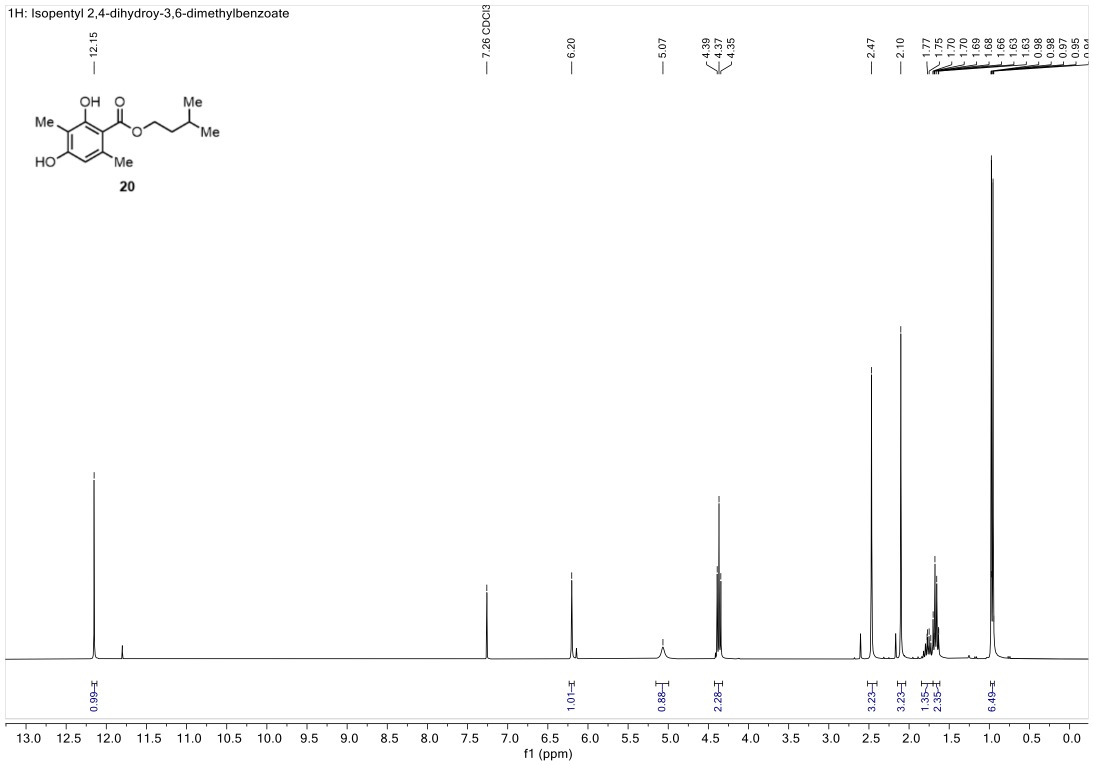

Fig. S17 ^{1}H-NMR spectrum of isopentyl 2,4-dihydroy-3,6-dimethylbenzoate (**20**), measured in $CDCl_3$ at 300 MHz.

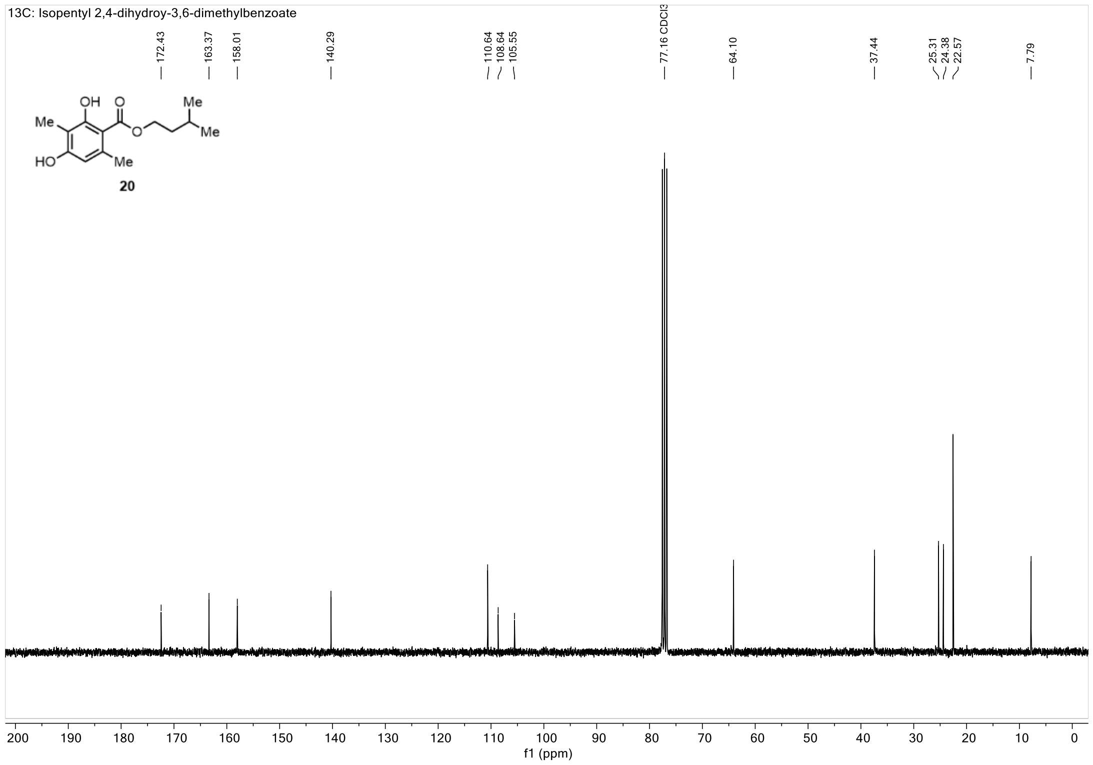

Fig. S18 ^{13}C-NMR spectrum of isopentyl 2,4-dihydroy-3,6-dimethylbenzoate (**20**), measured in $CDCl_3$ at 75 MHz.

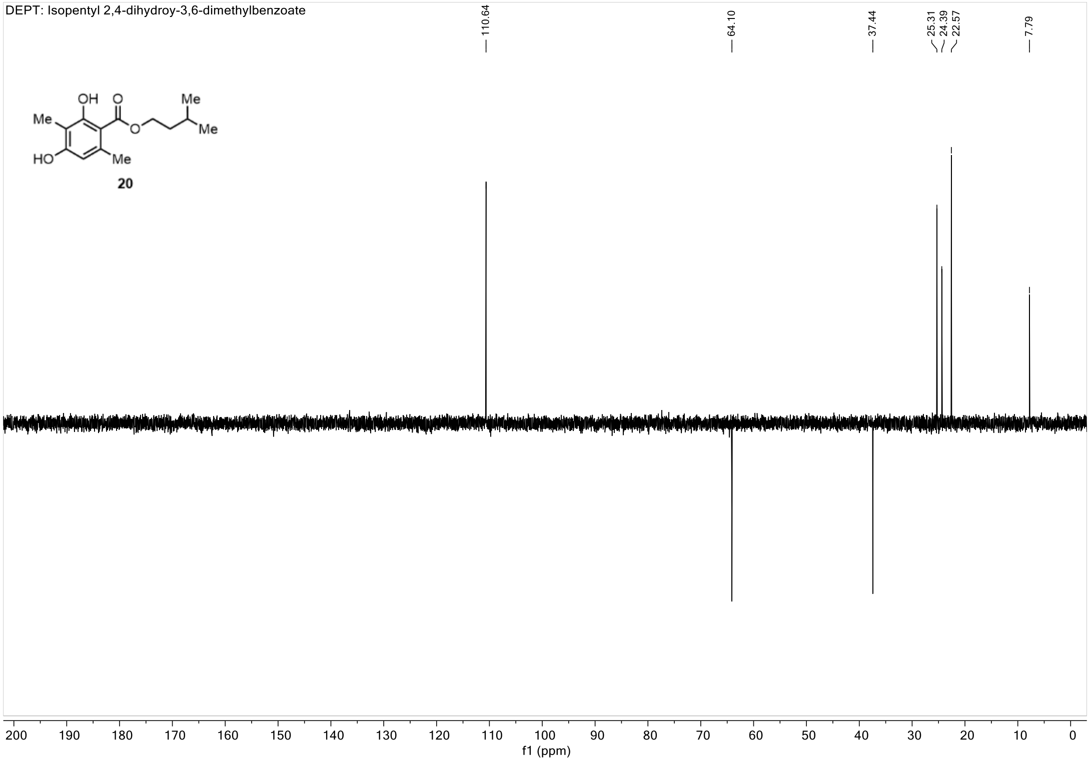

Fig. S19 DEPT spectrum of isopentyl 2,4-dihydroy-3,6-dimethylbenzoate (**20**), measured in $CDCl_3$ at 75 MHz.

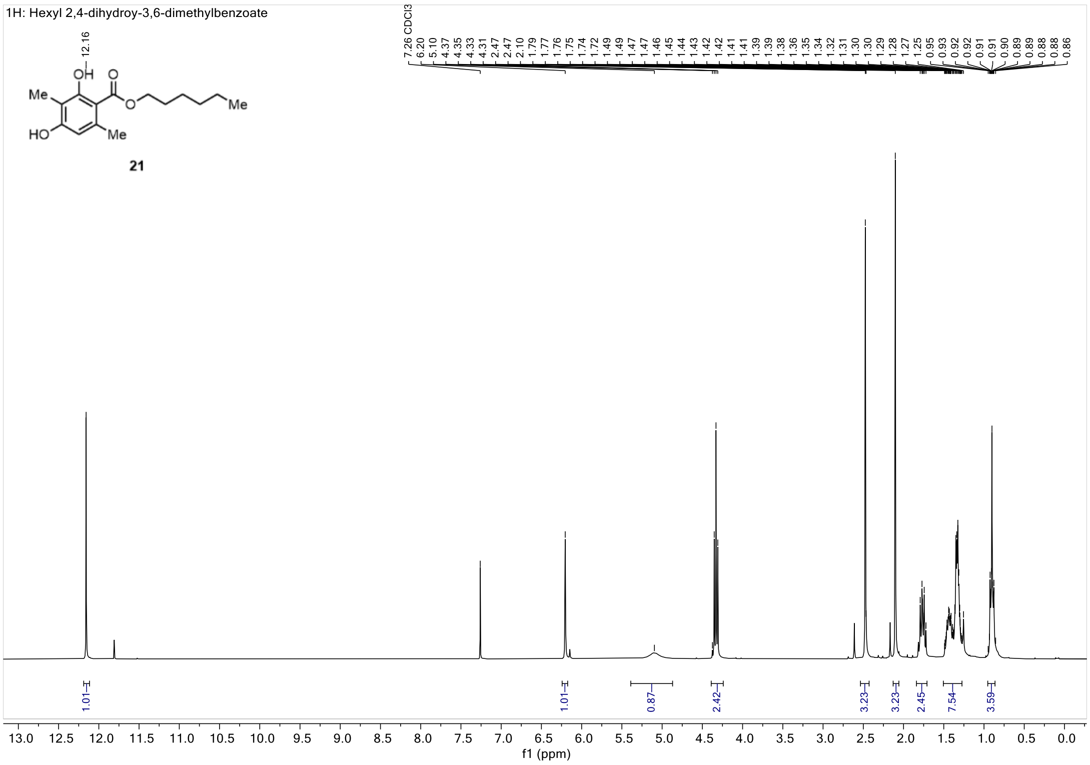

Fig. S20 ^{1}H-NMR spectrum of hexyl 2,4-dihydroy-3,6-dimethylbenzoate (**21**), measured in $CDCl_3$ at 300 MHz.

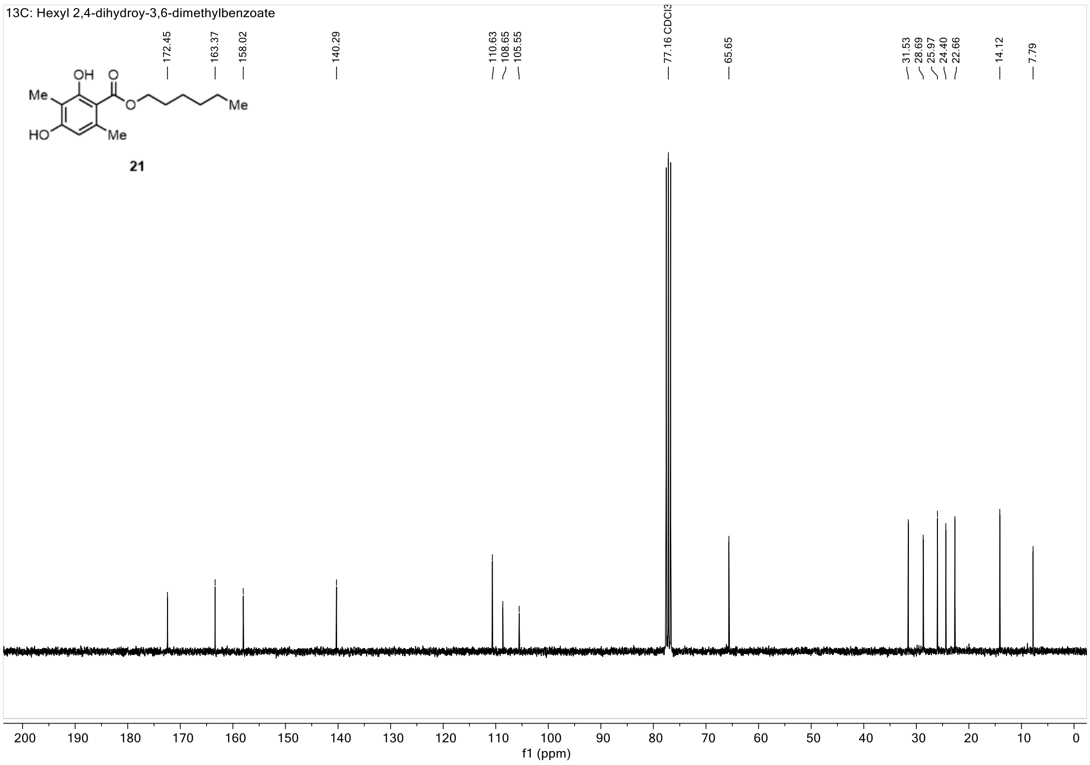

Fig. S21 ^{13}C-NMR spectrum of hexyl 2,4-dihydroy-3,6-dimethylbenzoate (**21**), measured in $CDCl_3$ at 75 MHz.

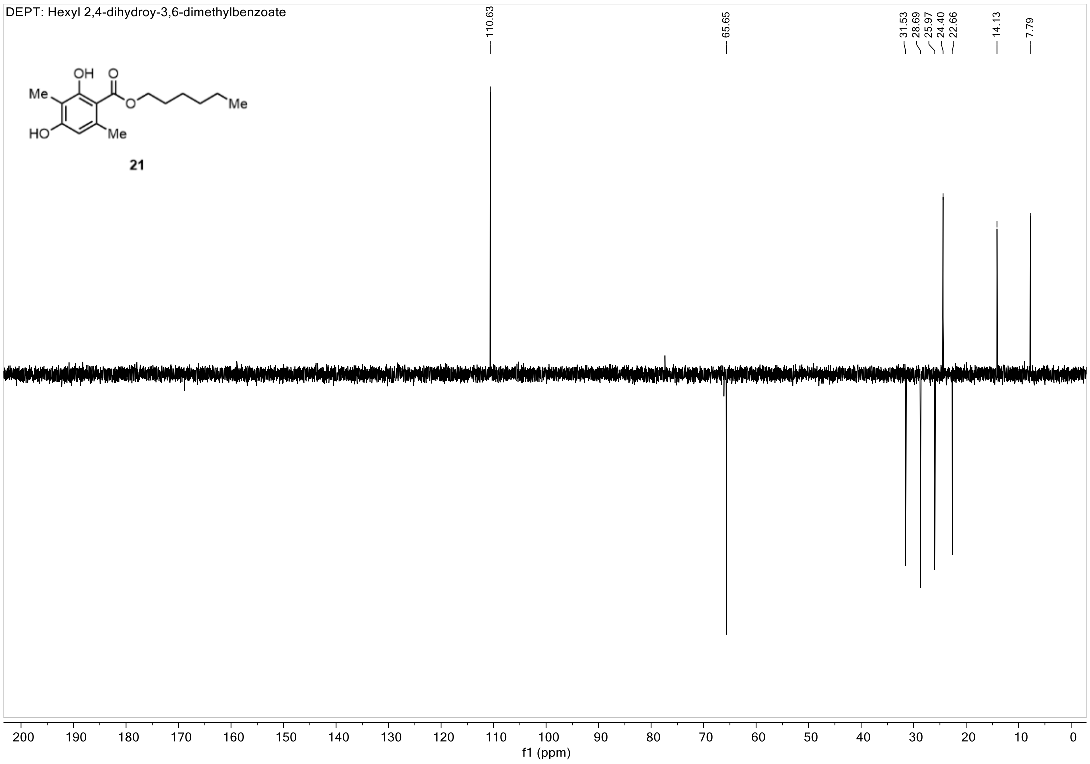

Fig. S22 DEPT spectrum of hexyl 2,4-dihydroy-3,6-dimethylbenzoate (**21**), measured in $CDCl_3$ at 75 MHz.

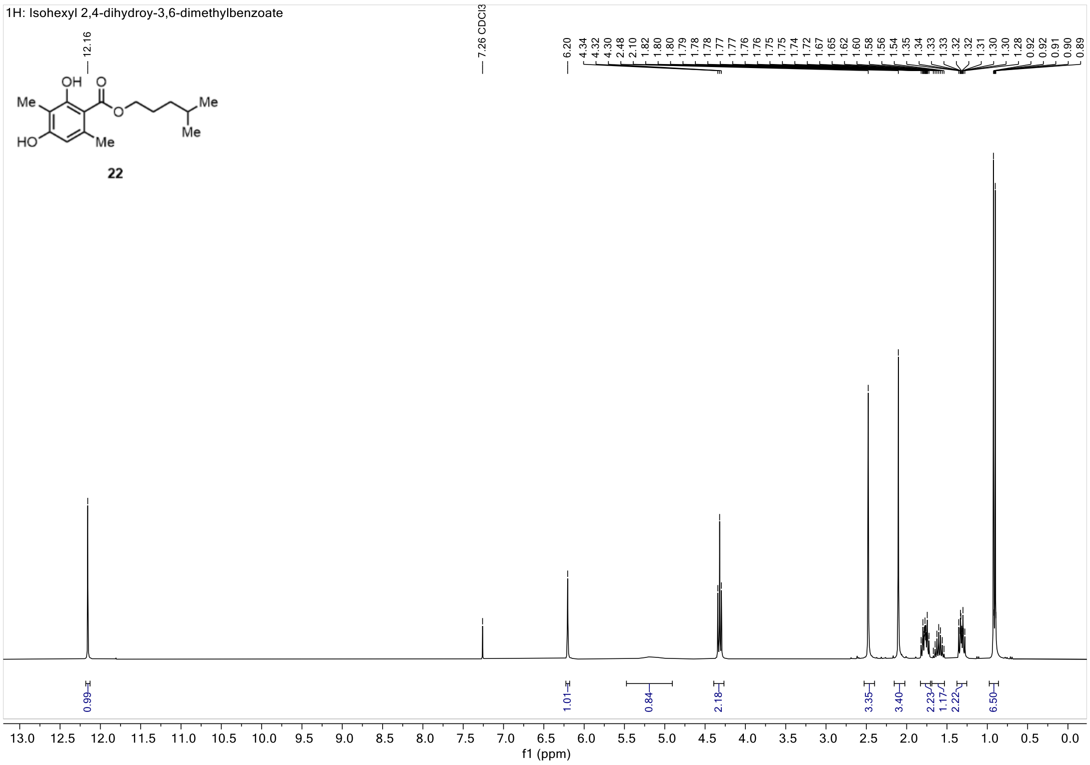

Fig. S23 ^{1}H-NMR spectrum of isohexyl 2,4-dihydroy-3,6-dimethylbenzoate (**22**), measured in $CDCl_3$ at 300 MHz.

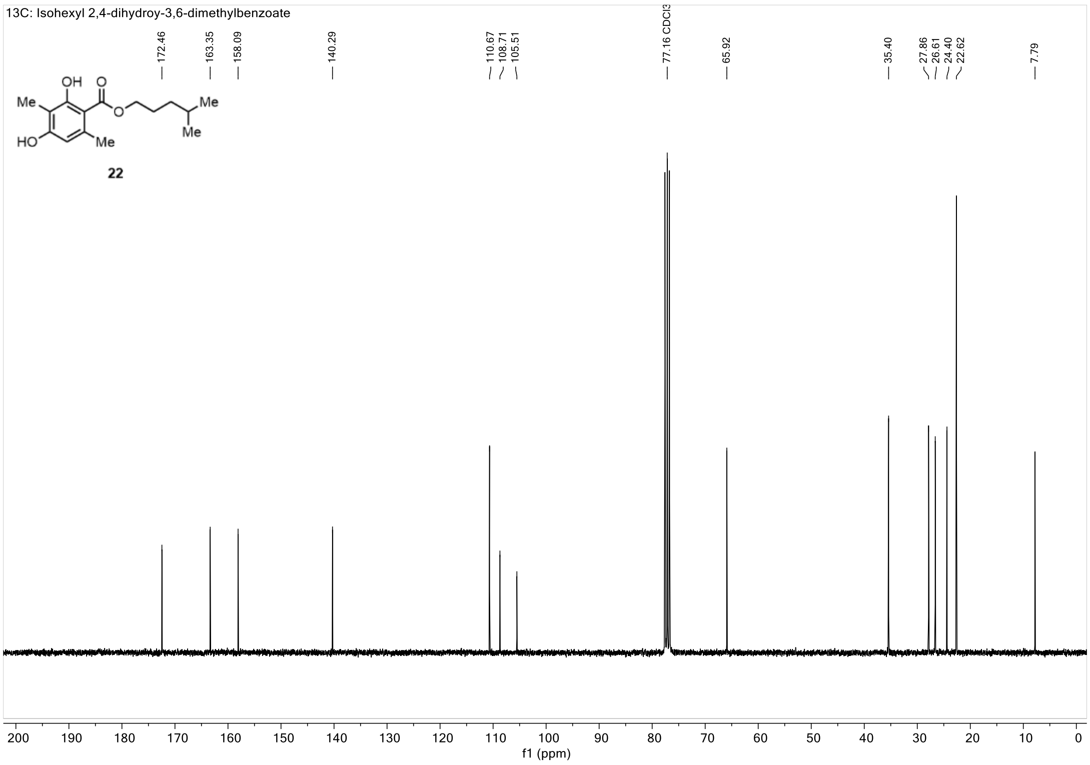

Fig. S24 ^{13}C-NMR spectrum of isohexyl 2,4-dihydroy-3,6-dimethylbenzoate (**22**), measured in $CDCl_3$ at 75 MHz.

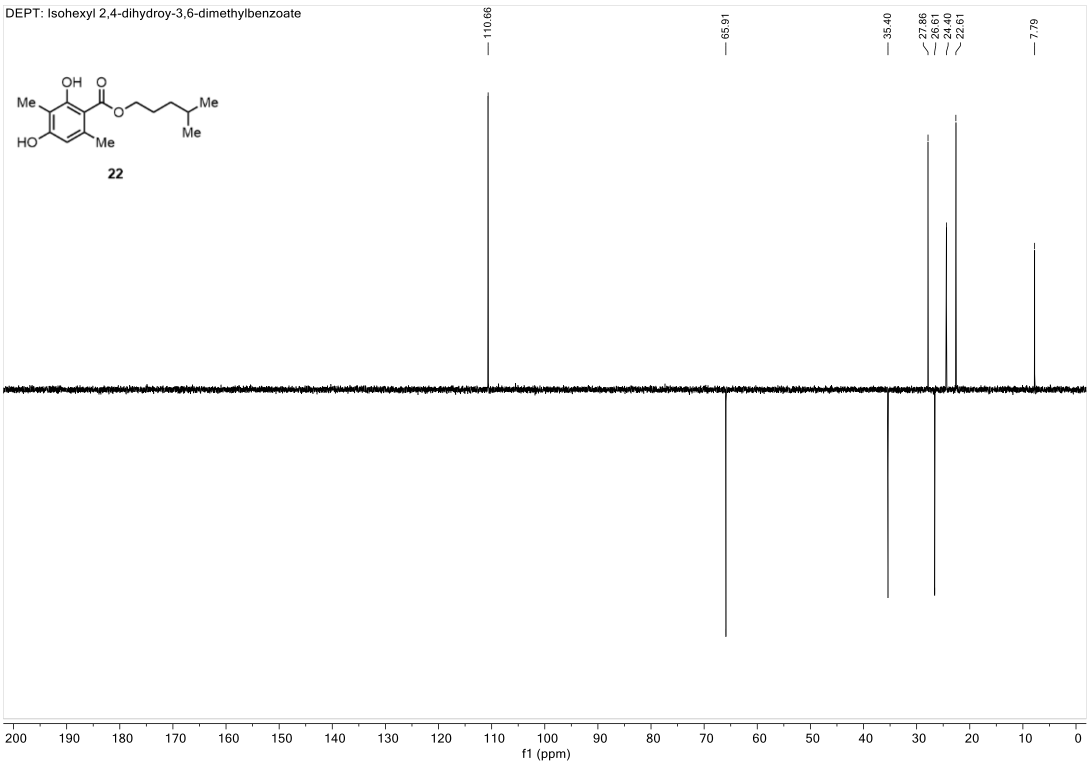

Fig. S25 DEPT spectrum of isohexyl 2,4-dihydroy-3,6-dimethylbenzoate (**22**), measured in $CDCl_3$ at 75 MHz.

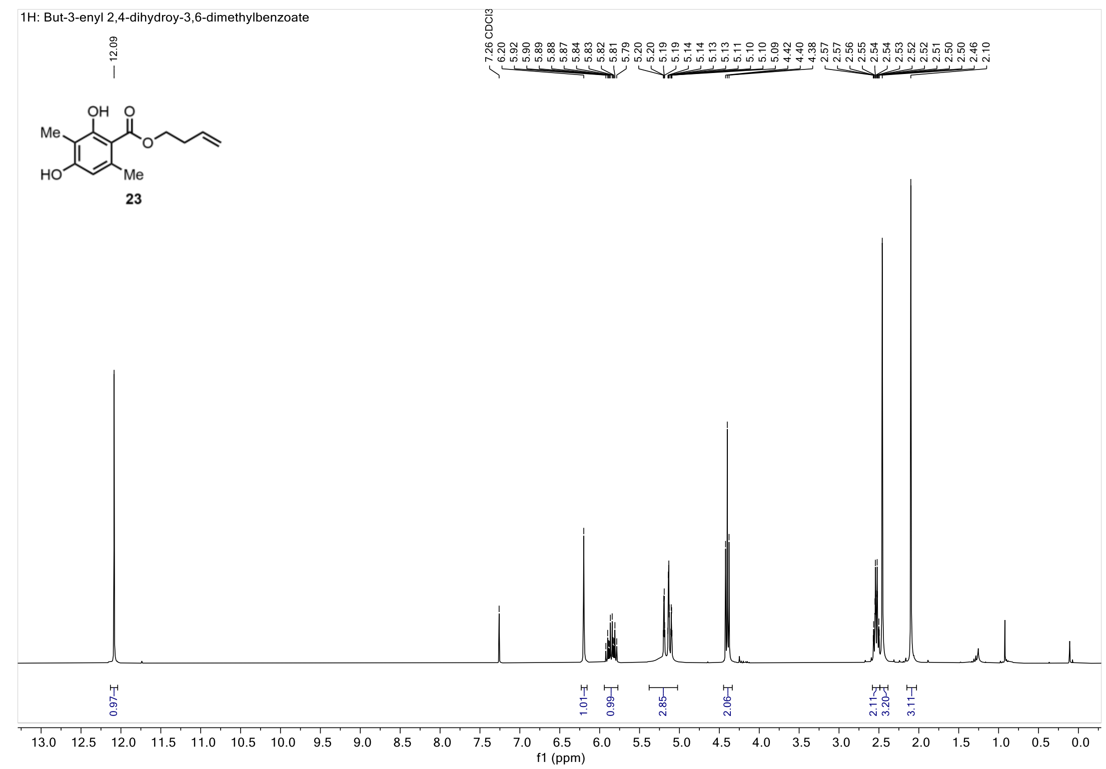

Fig. S26 ^{1}H-NMR spectrum of but-3-enyl 2,4-dihydroy-3,6-dimethylbenzoate (**23**), measured in $CDCl_3$ at 300 MHz.

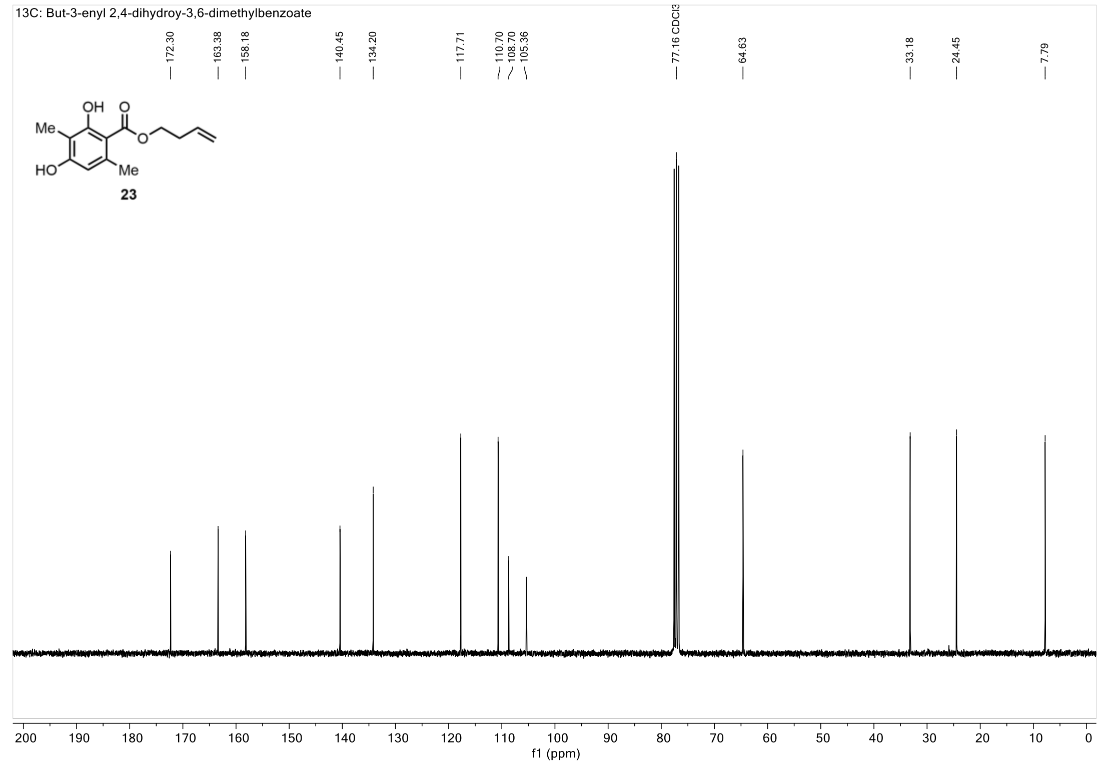

Fig. S27 ^{13}C-NMR spectrum of but-3-enyl 2,4-dihydroy-3,6-dimethylbenzoate (**23**), measured in $CDCl_3$ at 75 MHz.

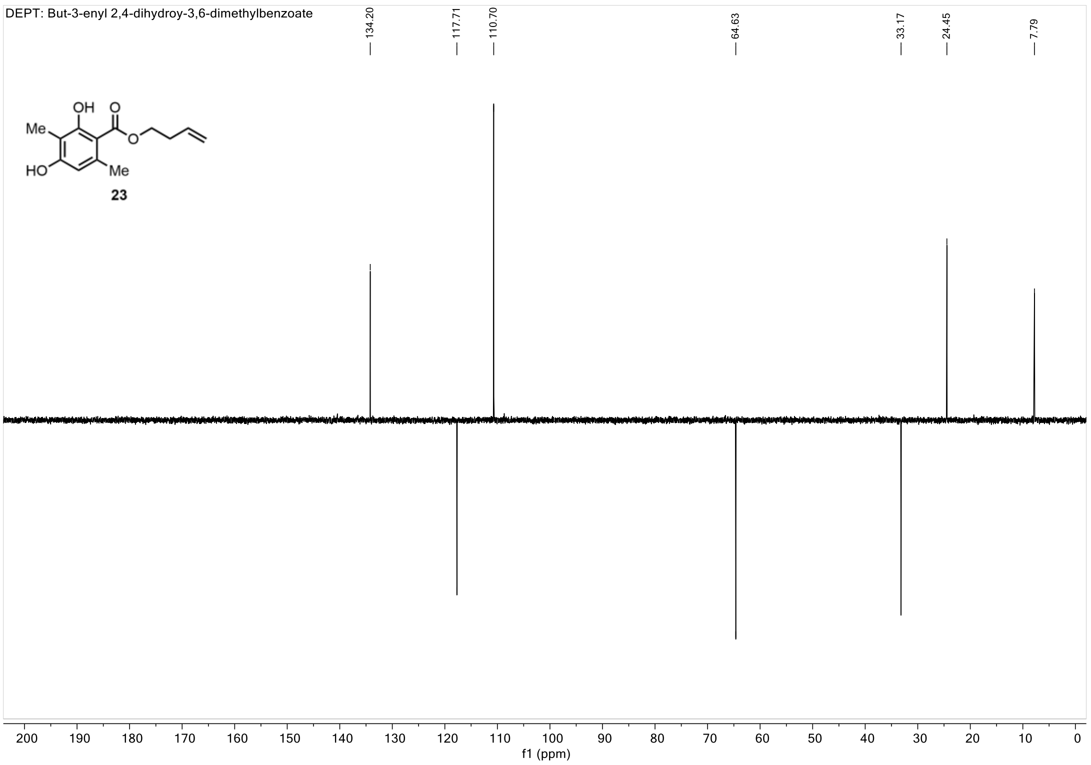

Fig. S28 DEPT spectrum of but-3-enyl 2,4-dihydroy-3,6-dimethylbenzoate (**23**), measured in $CDCl_3$ at 75 MHz.

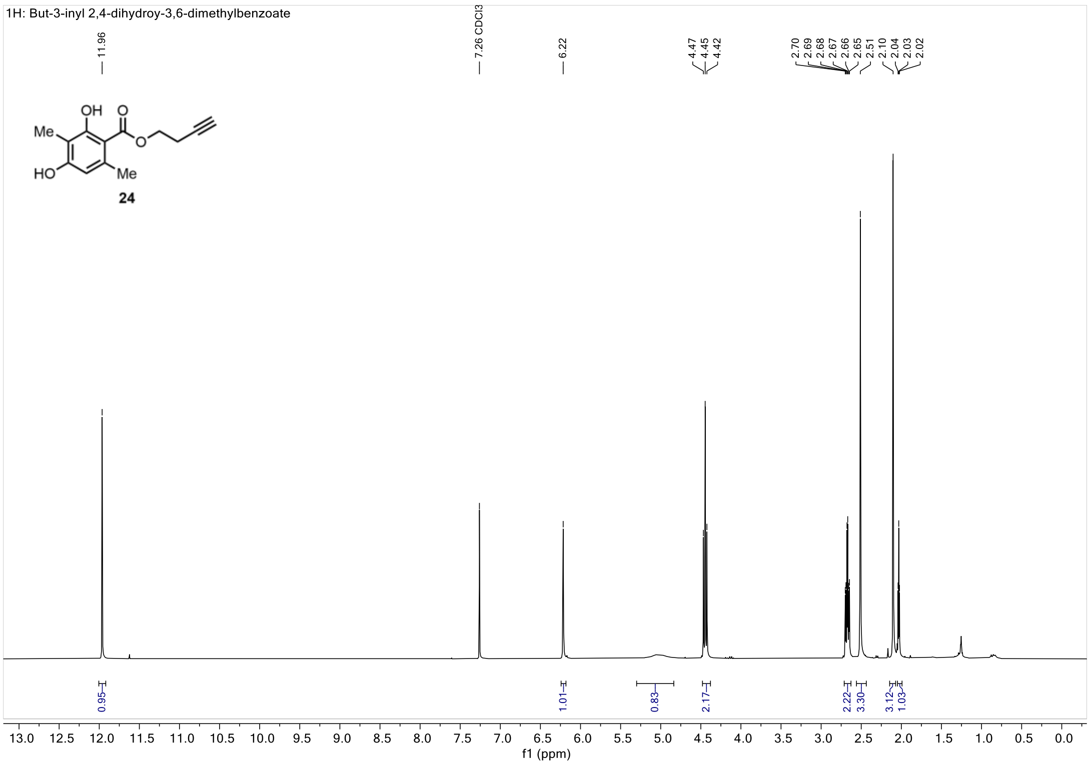

Fig. S29 ^{1}H-NMR spectrum of but-3-inyl 2,4-dihydroy-3,6-dimethylbenzoate (**24**), measured in $CDCl_3$ at 300 MHz.

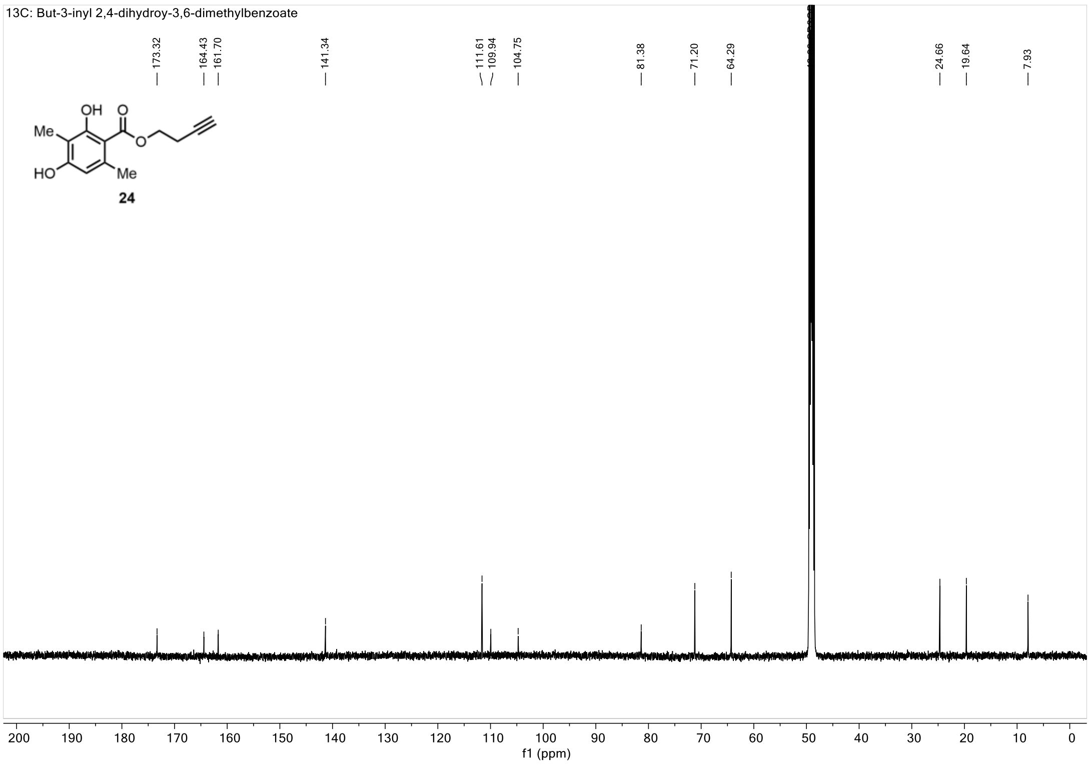

Fig. S30 ^{13}C-NMR spectrum of but-3-inyl 2,4-dihydroy-3,6-dimethylbenzoate (**24**), measured in $MeOD_4$ at 75 MHz.

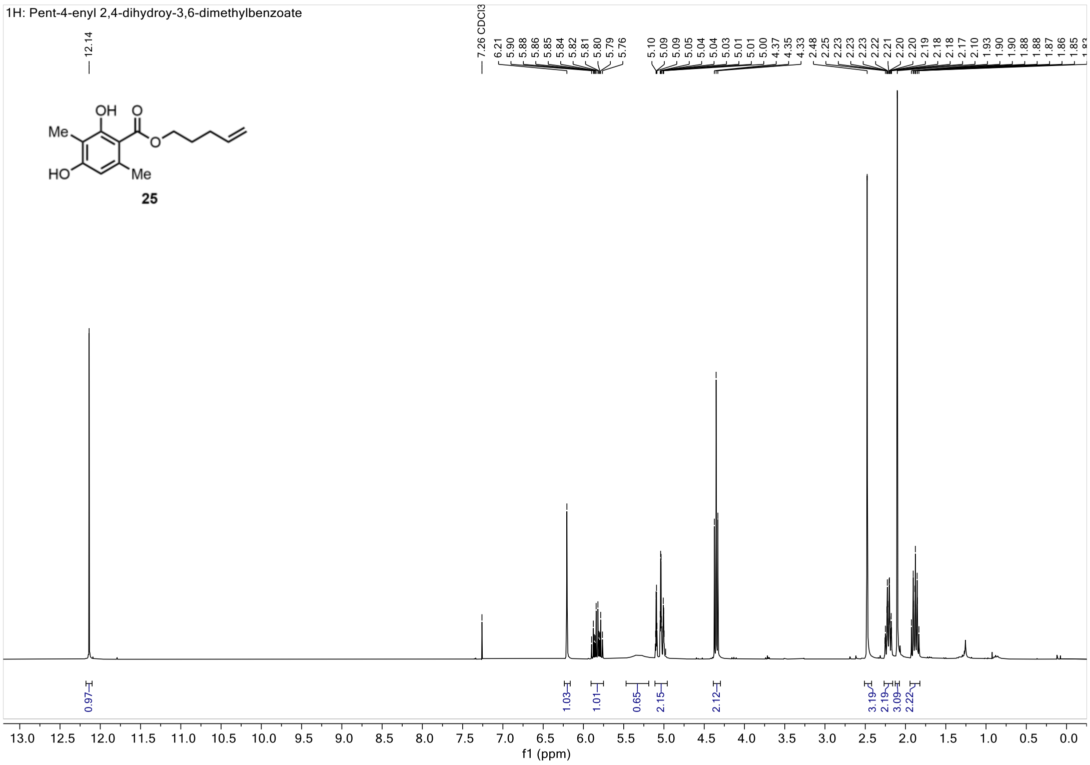

Fig. S31 ^{1}H-NMR spectrum of pent-4-enyl 2,4-dihydroy-3,6-dimethylbenzoate (**25**), measured in $CDCl_3$ at 300 MHz.

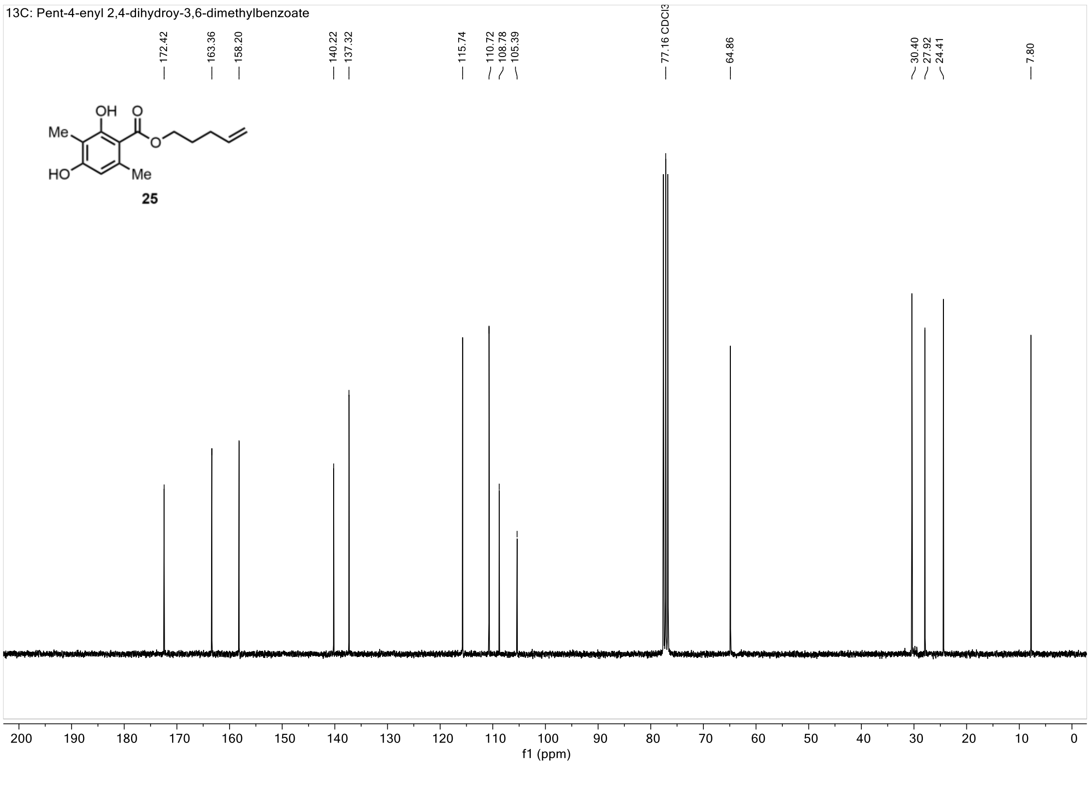

Fig. S32 ^{13}C-NMR spectrum of pent-4-enyl 2,4-dihydroy-3,6-dimethylbenzoate (**25**), measured in $CDCl_3$ at 75 MHz.

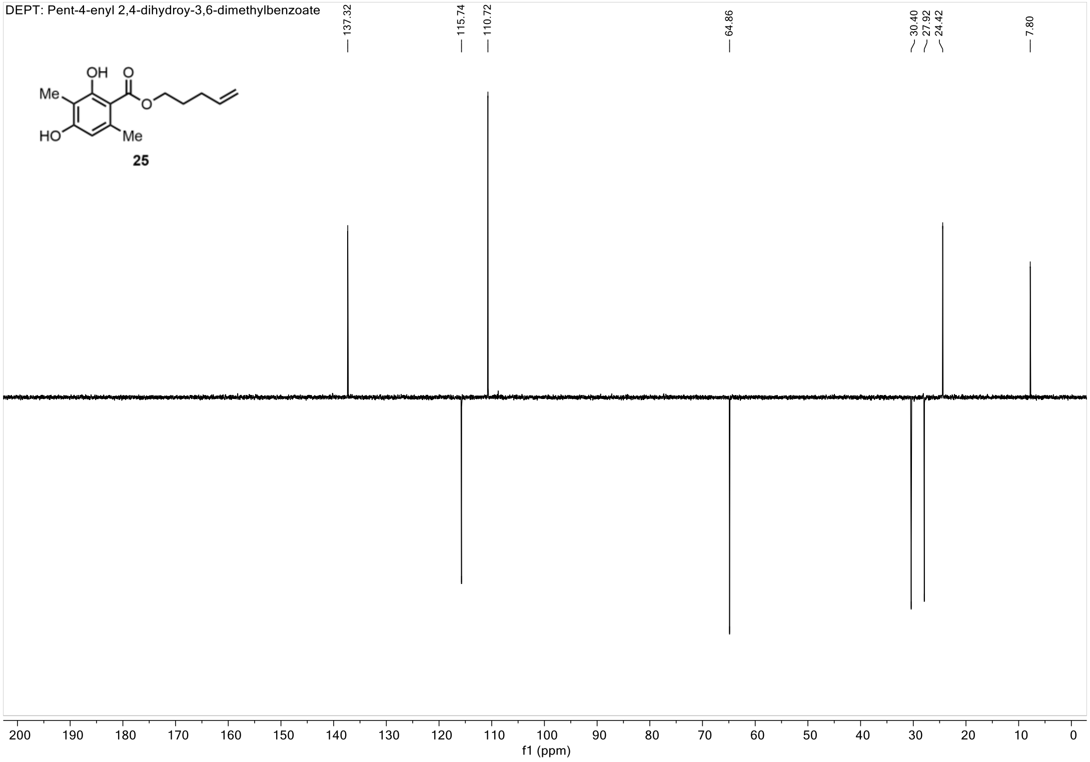

Fig. S33 DEPT spectrum of pent-4-enyl 2,4-dihydroy-3,6-dimethylbenzoate (**25**), measured in $CDCl_3$ at 75 MHz.

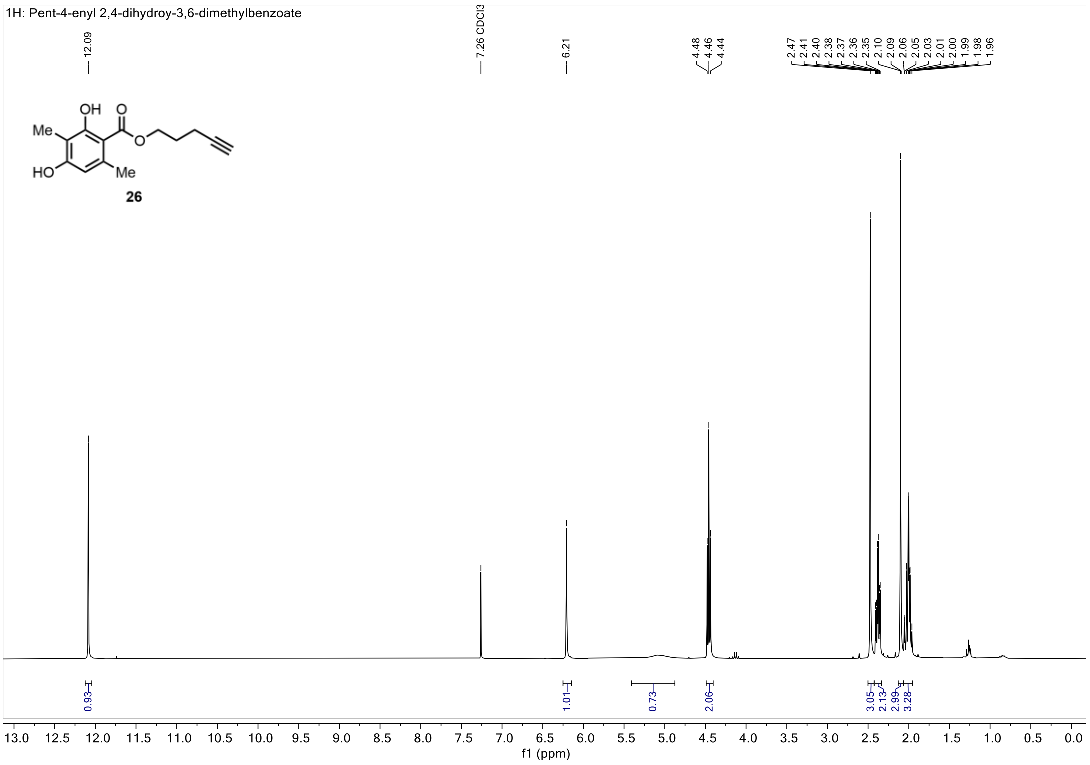

Fig. S34 ^{1}H-NMR spectrum of pent-4-inyl 2,4-dihydroy-3,6-dimethylbenzoate (**26**), measured in $CDCl_3$ at 300 MHz.

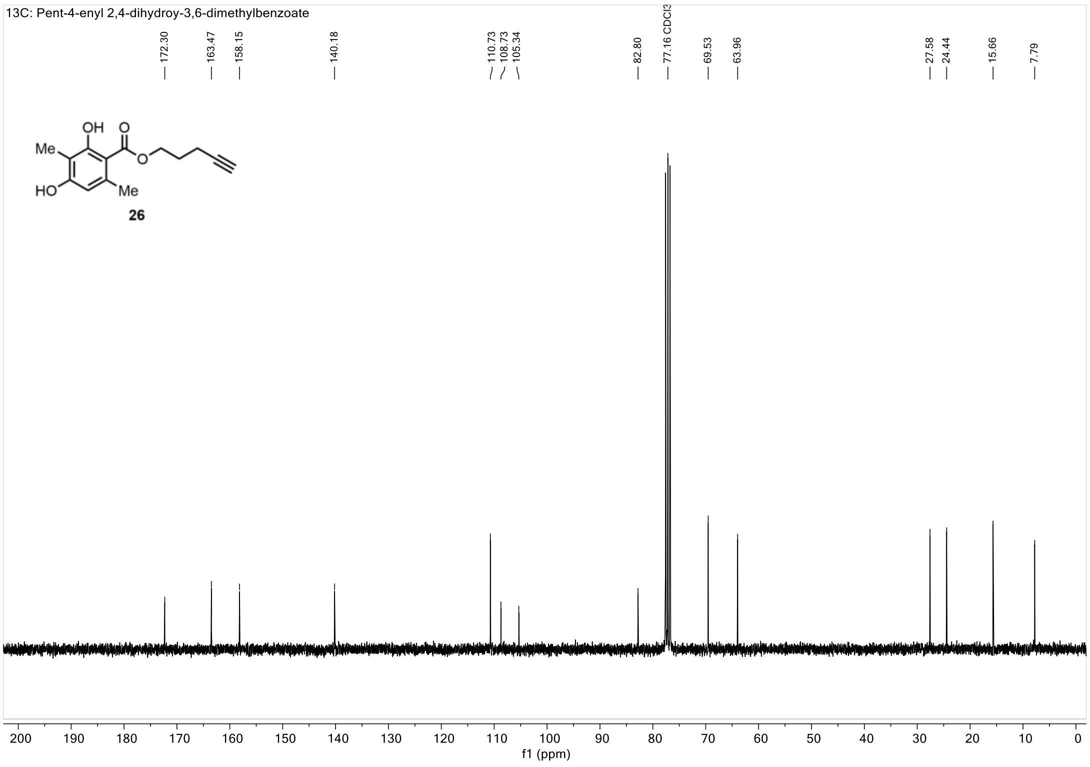

Fig. S35 ^{13}C-NMR spectrum of pent-4-inyl 2,4-dihydroy-3,6-dimethylbenzoate (**26**), measured in $CDCl_3$ at 75 MHz.

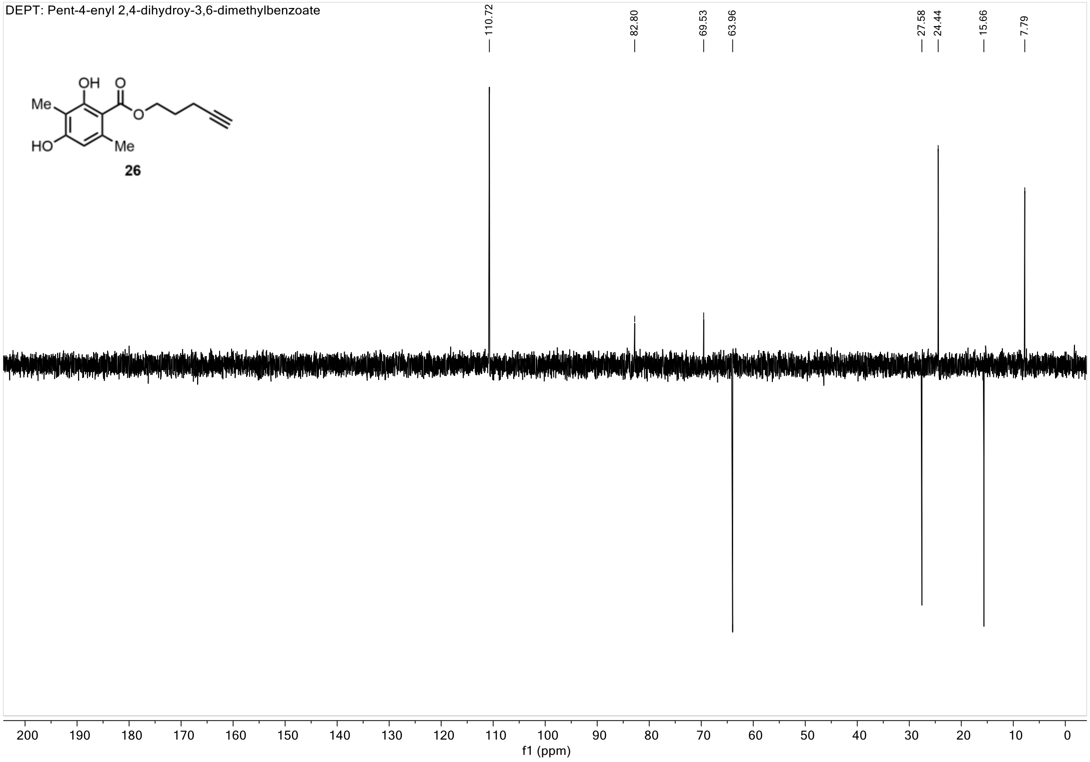

Fig. S36 DEPT spectrum of pent-4-inyl 2,4-dihydroy-3,6-dimethylbenzoate (**26**), measured in $CDCl_3$ at 75 MHz.

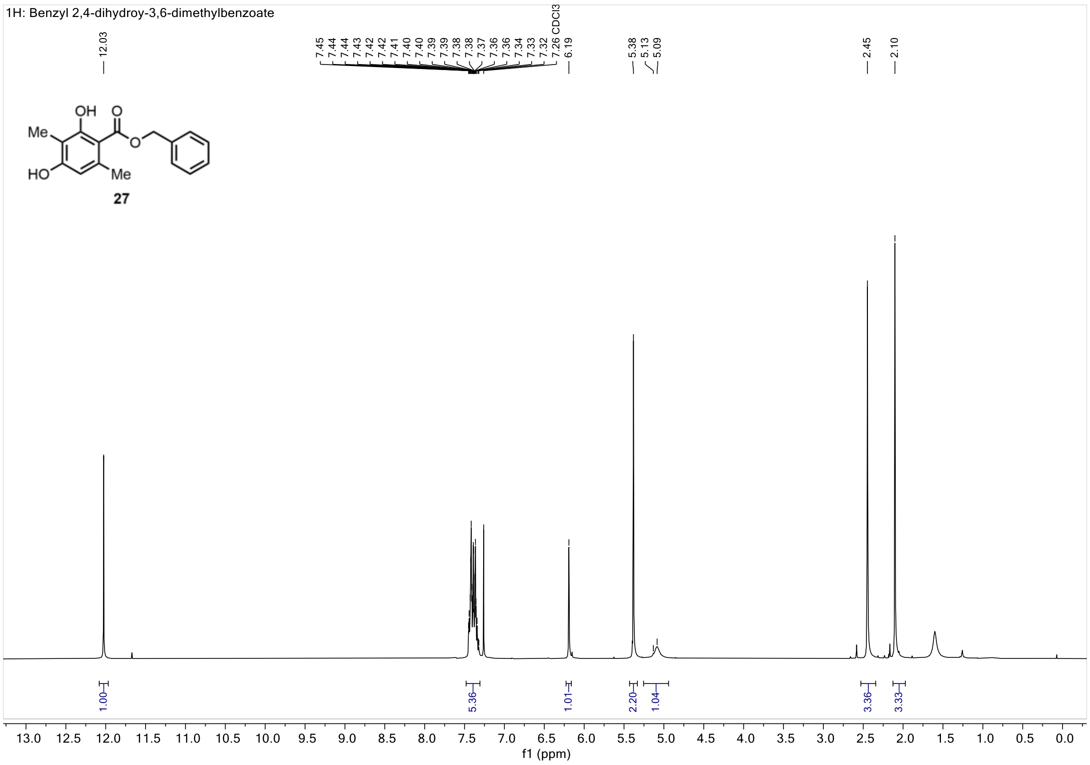

Fig. S37 ^{1}H-NMR spectrum of benzyl 2,4-dihydroy-3,6-dimethylbenzoate (**27**), measured in $CDCl_3$ at 300 MHz.

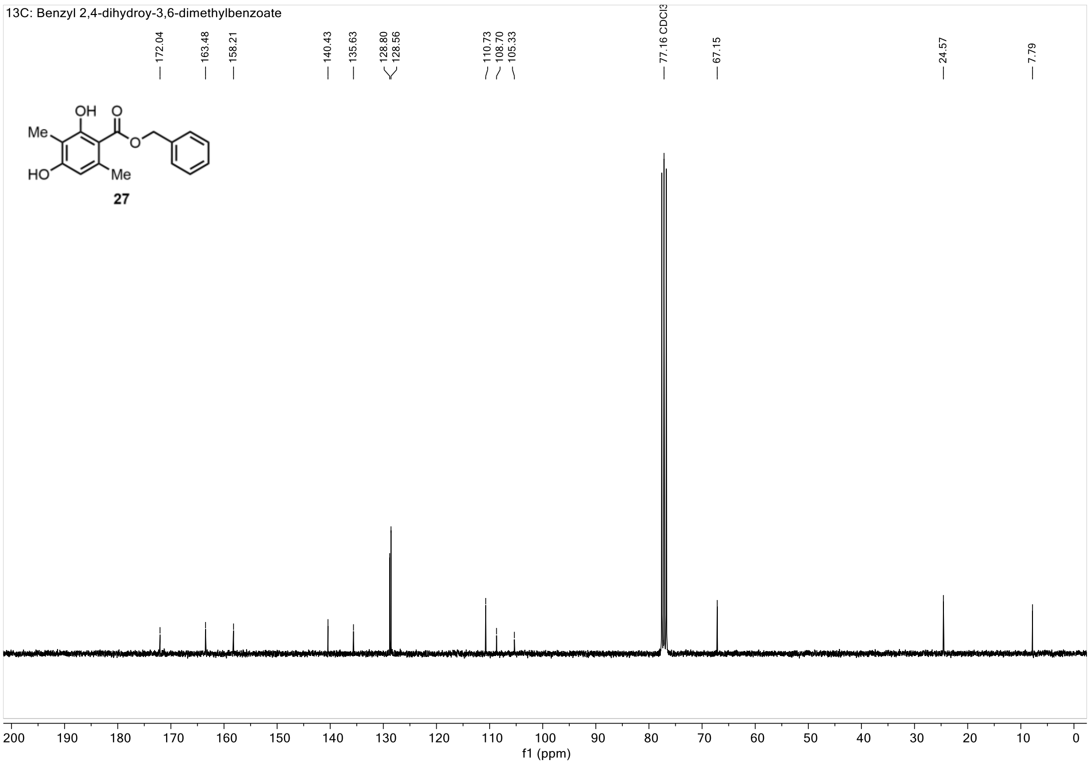

Fig. S38 ^{13}C-NMR spectrum of benzyl 2,4-dihydroy-3,6-dimethylbenzoate (**27**), measured in $CDCl_3$ at 75 MHz.

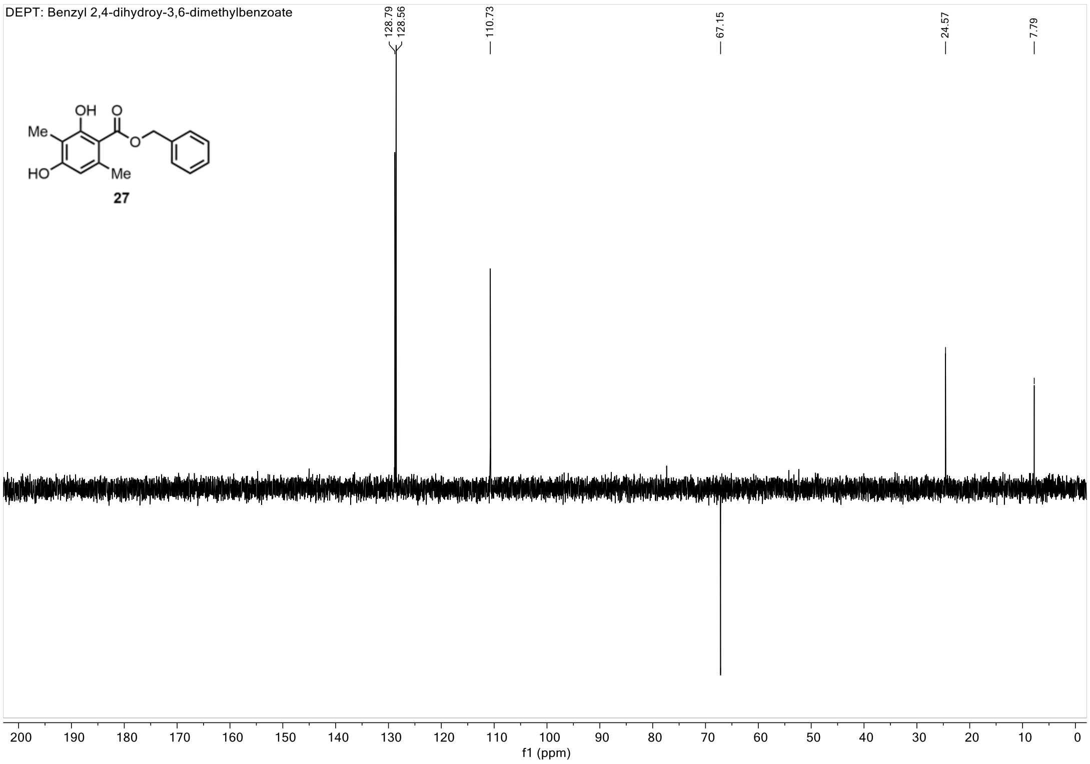

Fig. S39 DEPT spectrum of benzyl 2,4-dihydroy-3,6-dimethylbenzoate (**27**), measured in $CDCl_3$ at 75 MHz.

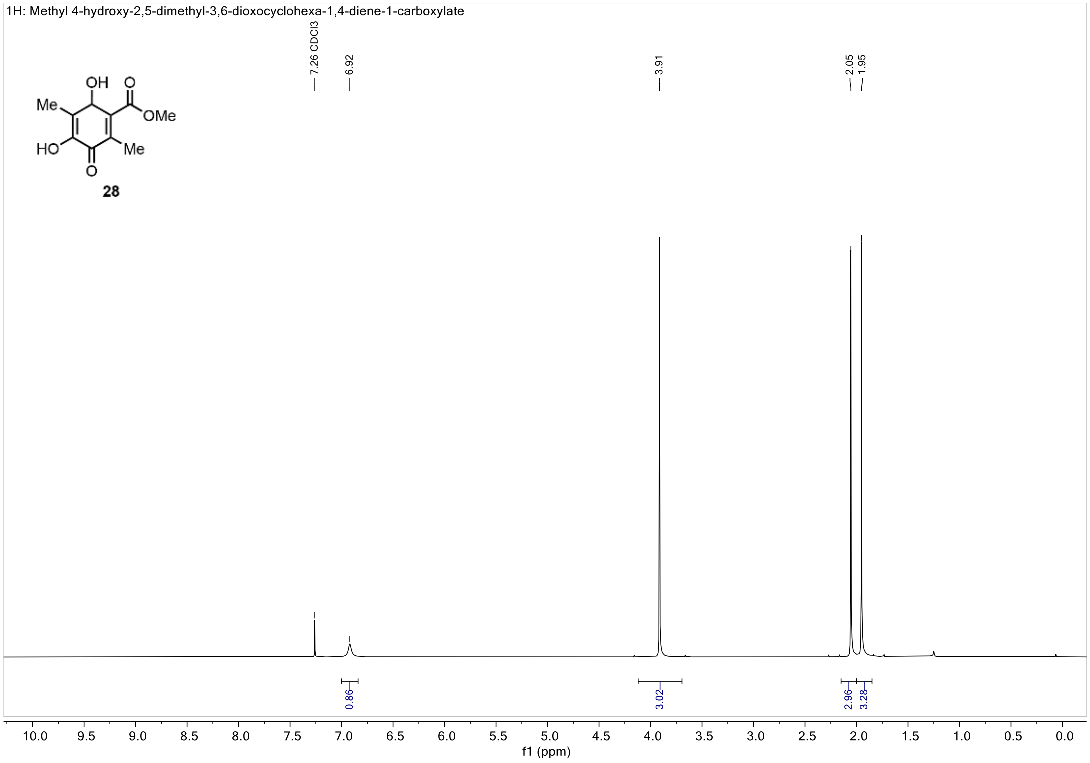

Fig. S40 ^{1}H-NMR spectrum of methyl 4-hydroxy-2,5-dimethyl-3,6-dioxocyclohexa-1,4-diene-1-carboxylate (**28**), measured in $CDCl_3$ at 300 MHz.

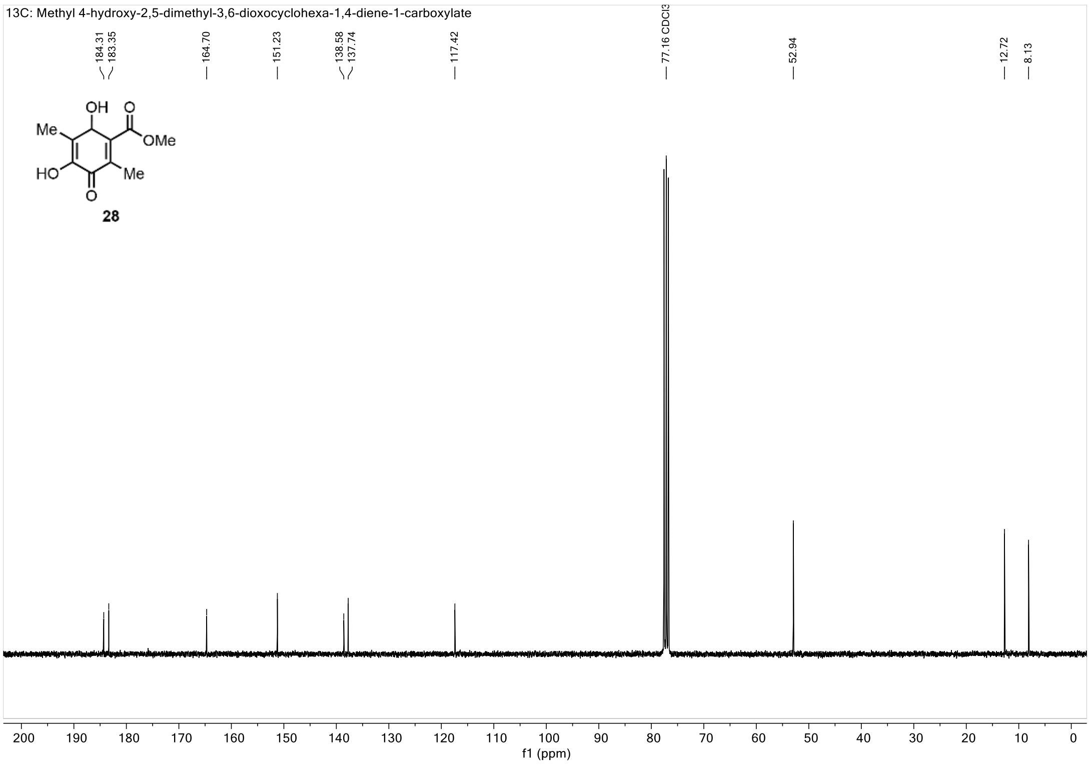

Fig. S41 ^{13}C-NMR spectrum of methyl 4-hydroxy-2,5-dimethyl-3,6-dioxocyclohexa-1,4-diene-1-carboxylate (**28**), measured in $CDCl_3$ at 75 MHz.

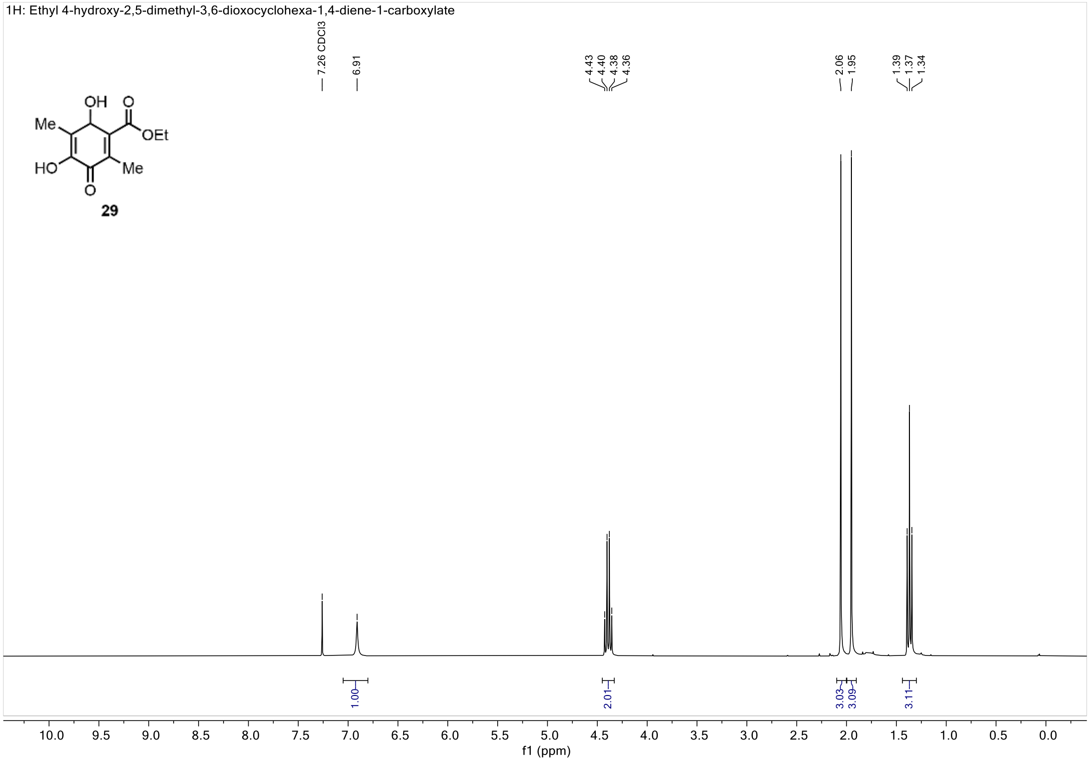

Fig. S42 ^{1}H-NMR spectrum of ethyl 4-hydroxy-2,5-dimethyl-3,6-dioxocyclohexa-1,4-diene-1-carboxylate (**29**), measured in $CDCl_3$ at 300 MHz.

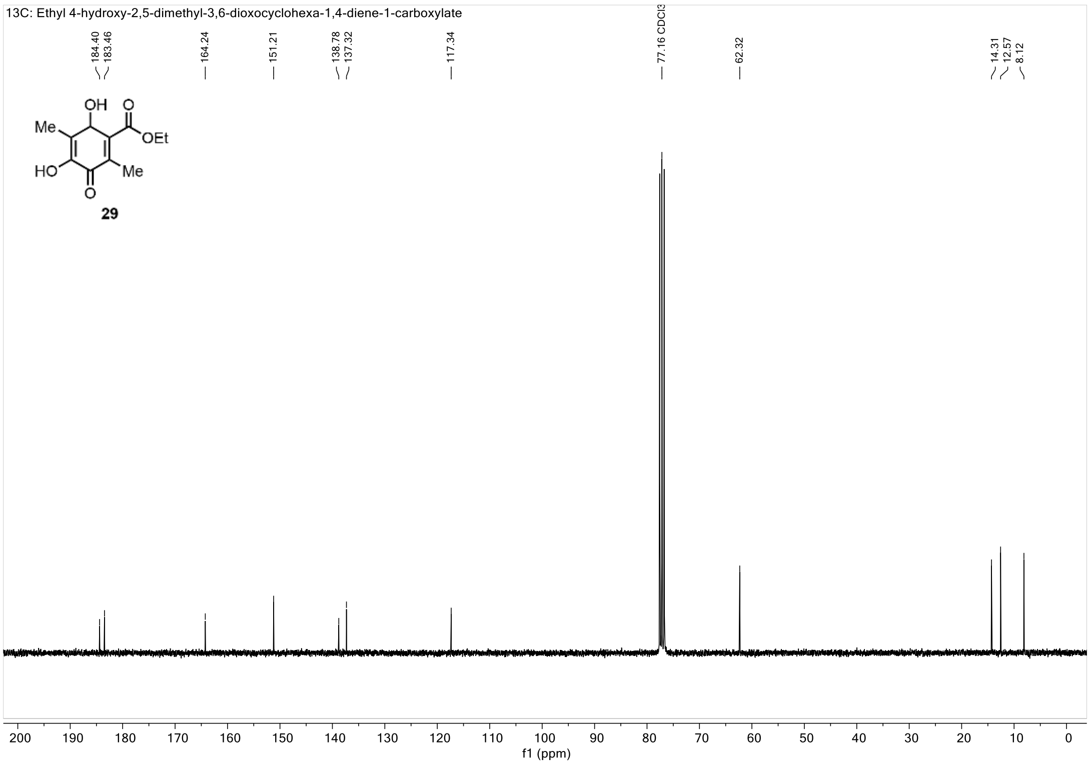

Fig. S43 ^{13}C-NMR spectrum of ethyl 4-hydroxy-2,5-dimethyl-3,6-dioxocyclohexa-1,4-diene-1-carboxylate (**29**), measured in $CDCl_3$ at 75 MHz.

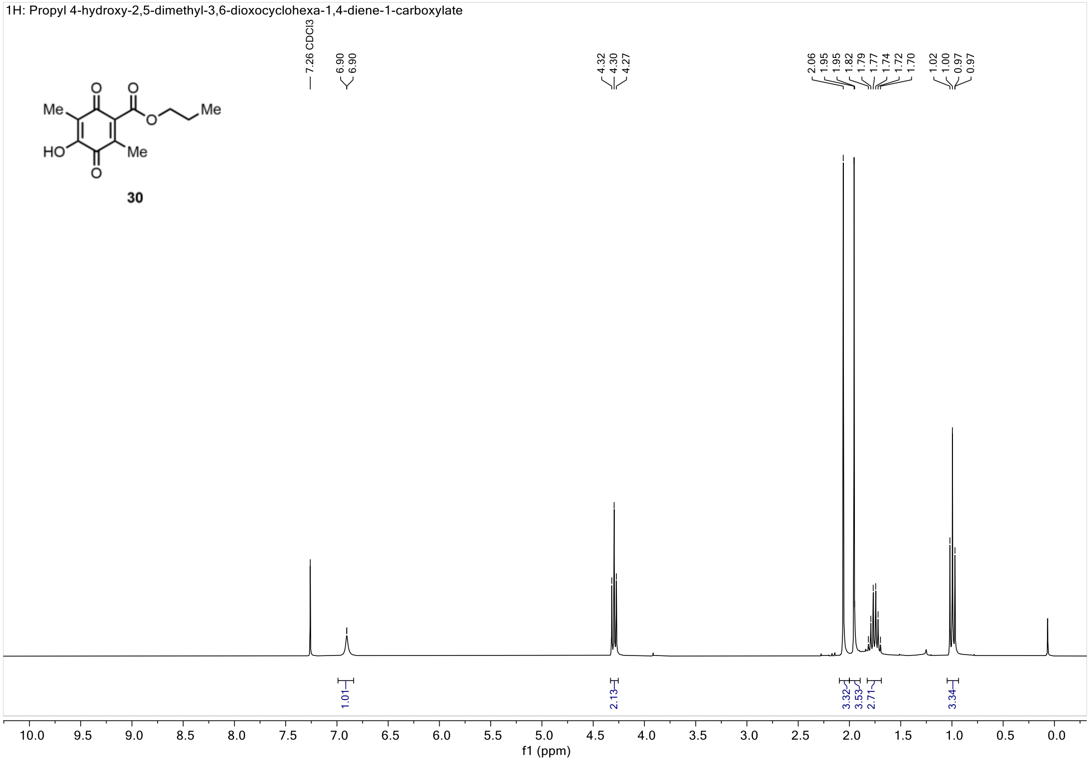

Fig. S44 ^{1}H-NMR spectrum of propyl 4-hydroxy-2,5-dimethyl-3,6-dioxocyclohexa-1,4-diene-1-carboxylate (**30**), measured in $CDCl_3$ at 300 MHz.

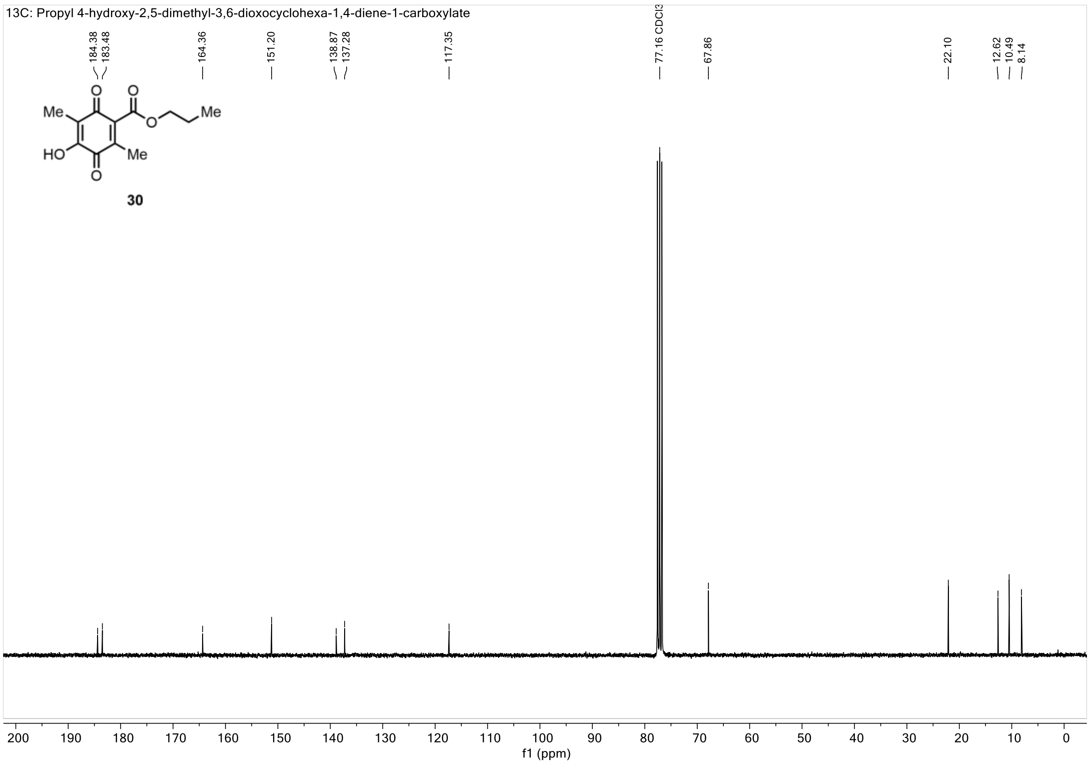

Fig. S45 ^{13}C-NMR spectrum of propyl 4-hydroxy-2,5-dimethyl-3,6-dioxocyclohexa-1,4-diene-1-carboxylate (**30**), measured in $CDCl_3$ at 75 MHz.

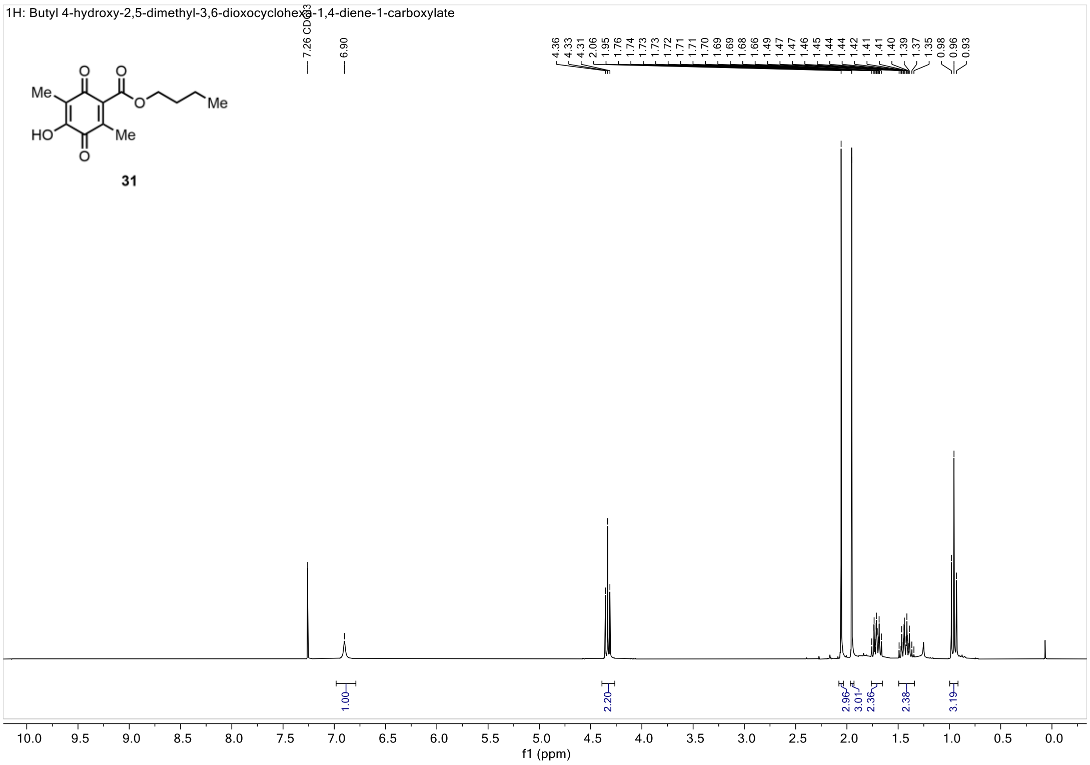

Fig. S46 ^{1}H-NMR spectrum of butyl 4-hydroxy-2,5-dimethyl-3,6-dioxocyclohexa-1,4-diene-1-carboxylate (**31**), measured in $CDCl_3$ at 300 MHz.

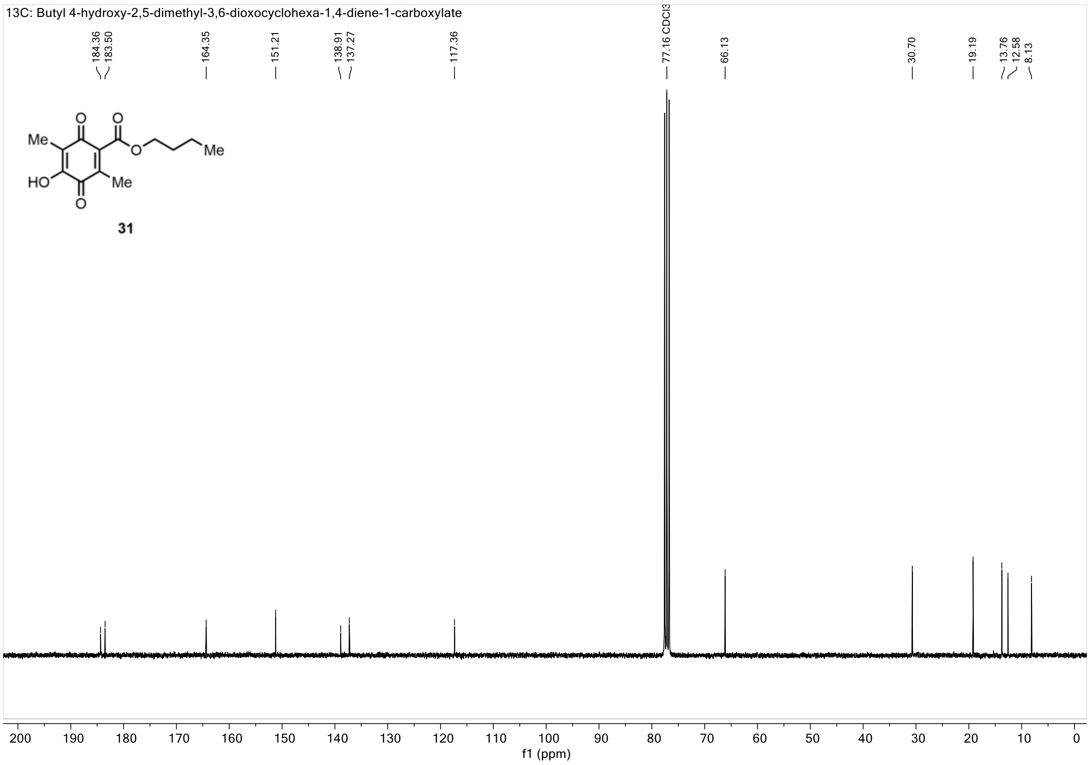

Fig. S47 ^{13}C-NMR spectrum of butyl 4-hydroxy-2,5-dimethyl-3,6-dioxocyclohexa-1,4-diene-1-carboxylate (**31**), measured in $CDCl_3$ at 75 MHz.

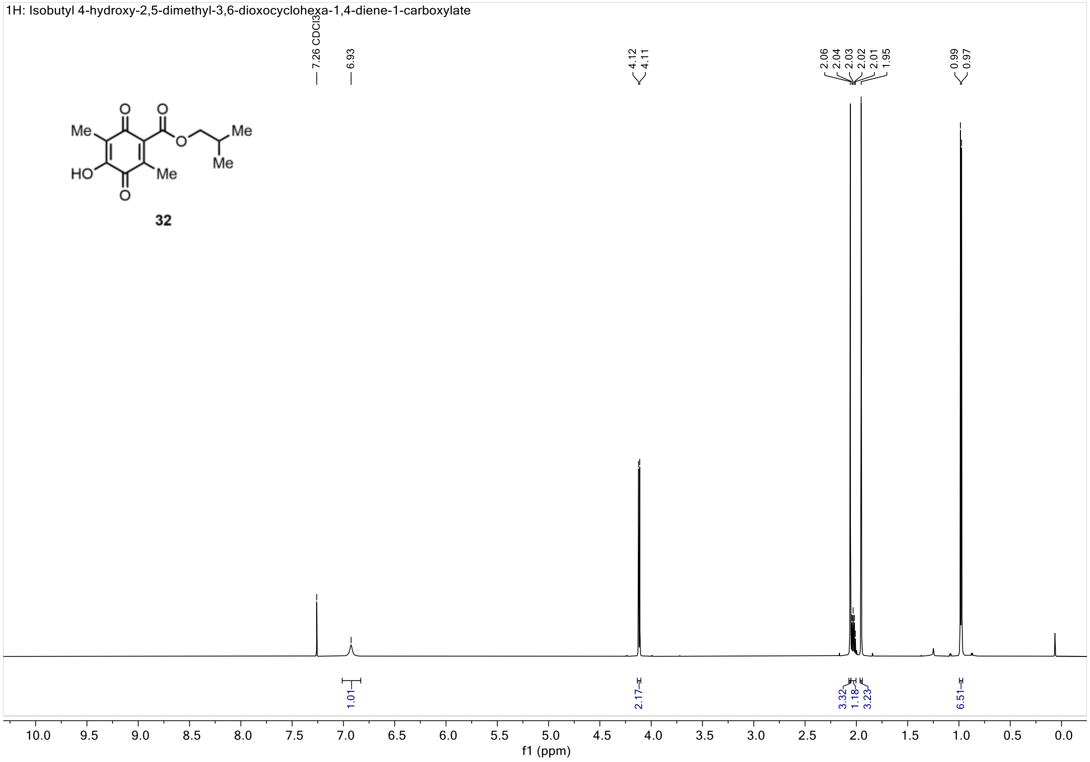

Fig. S48 ¹H-NMR spectrum of isobutyl 4-hydroxy-2,5-dimethyl-3,6-dioxocyclohexa-1,4-diene-1-carboxylate (**32**), measured in $CDCl_3$ at 600 MHz.

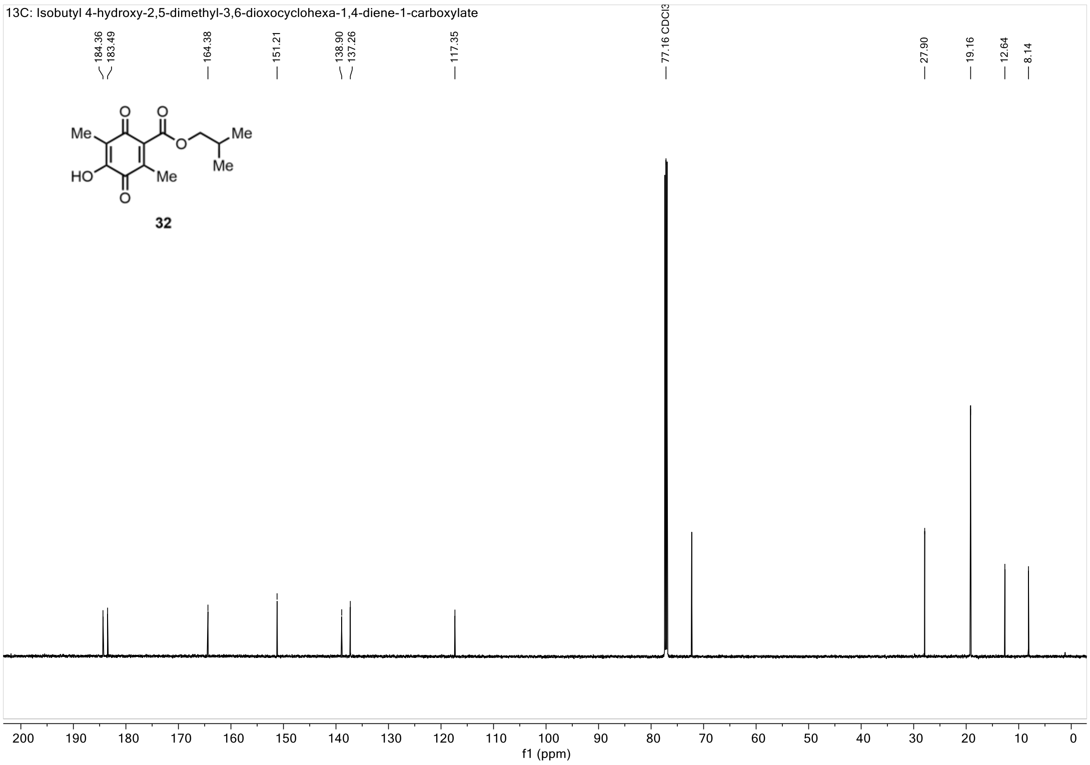

Fig. S49 ¹³C-NMR spectrum of isobutyl 4-hydroxy-2,5-dimethyl-3,6-dioxocyclohexa-1,4-diene-1-carboxylate (**32**), measured in $CDCl_3$ at 151 MHz.

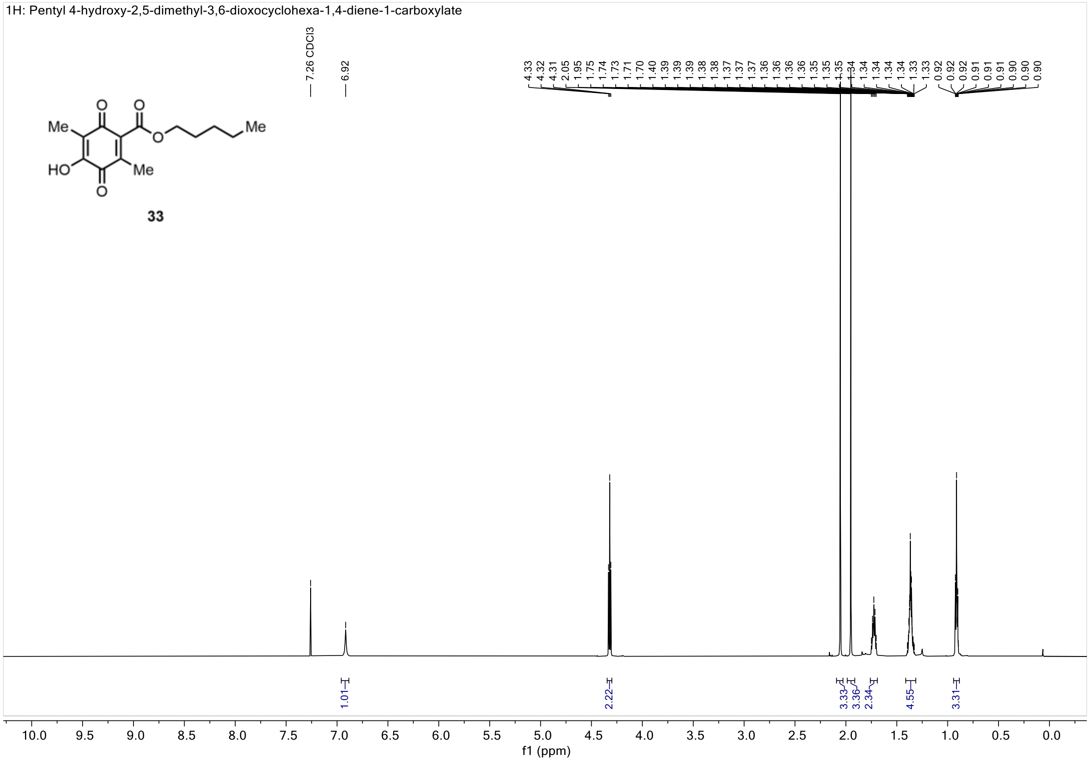

Fig. S50 ^{1}H-NMR spectrum of pentyl 4-hydroxy-2,5-dimethyl-3,6-dioxocyclohexa-1,4-diene-1-carboxylate (**33**), measured in $CDCl_3$ at 600 MHz.

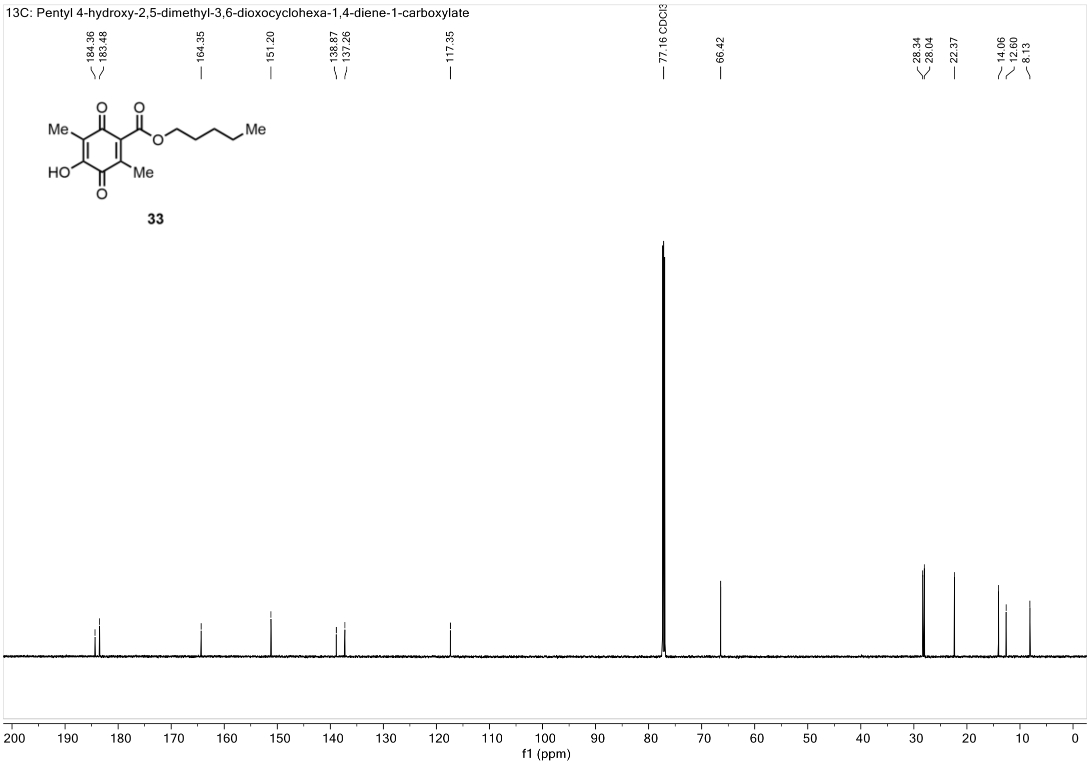

Fig. S51 ^{13}C-NMR spectrum of pentyl 4-hydroxy-2,5-dimethyl-3,6-dioxocyclohexa-1,4-diene-1-carboxylate (**33**), measured in $CDCl_3$ at 151 MHz.

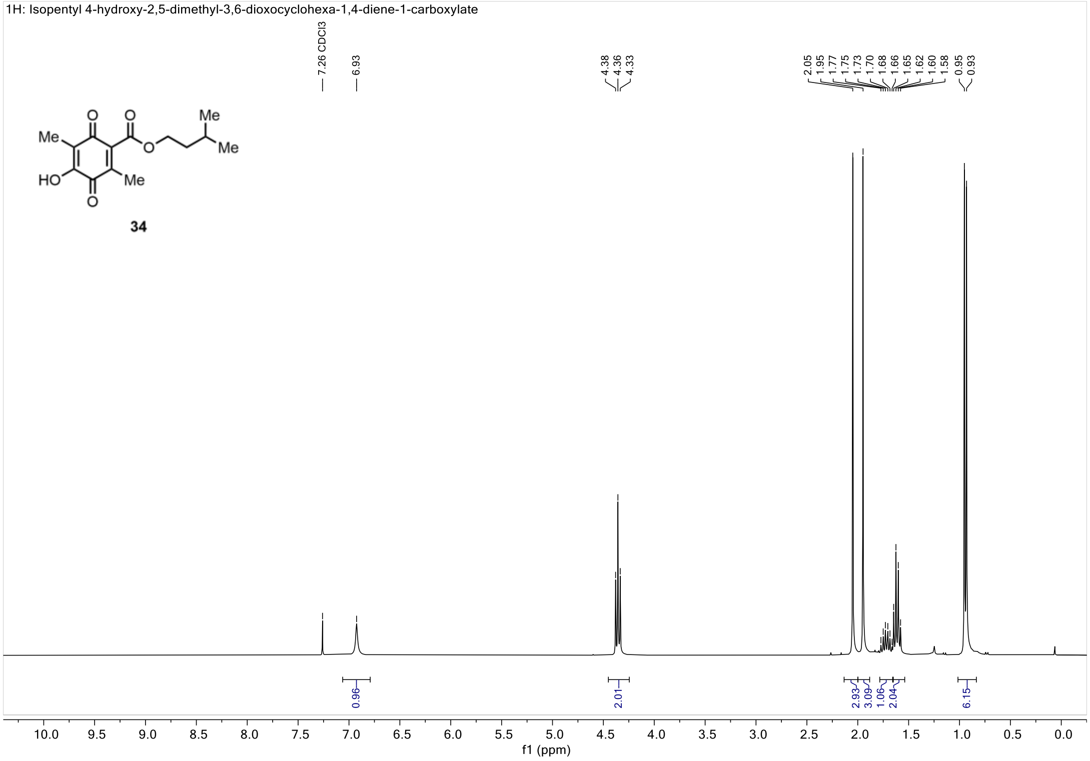

Fig. S52 ^{1}H-NMR spectrum of isopentyl 4-hydroxy-2,5-dimethyl-3,6-dioxocyclohexa-1,4-diene-1-carboxylate (**34**), measured in $CDCl_3$ at 300 MHz.

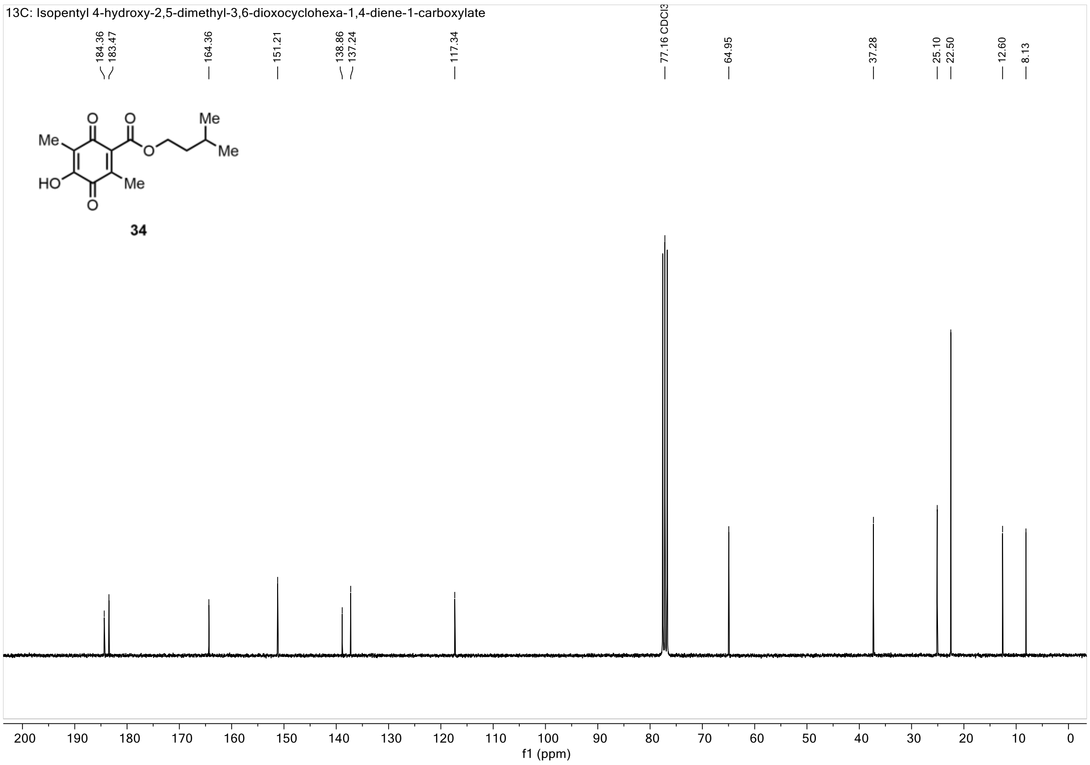

Fig. S53 ^{13}C-NMR spectrum of isopentyl 4-hydroxy-2,5-dimethyl-3,6-dioxocyclohexa-1,4-diene-1-carboxylate (**34**), measured in $CDCl_3$ at 75 MHz.

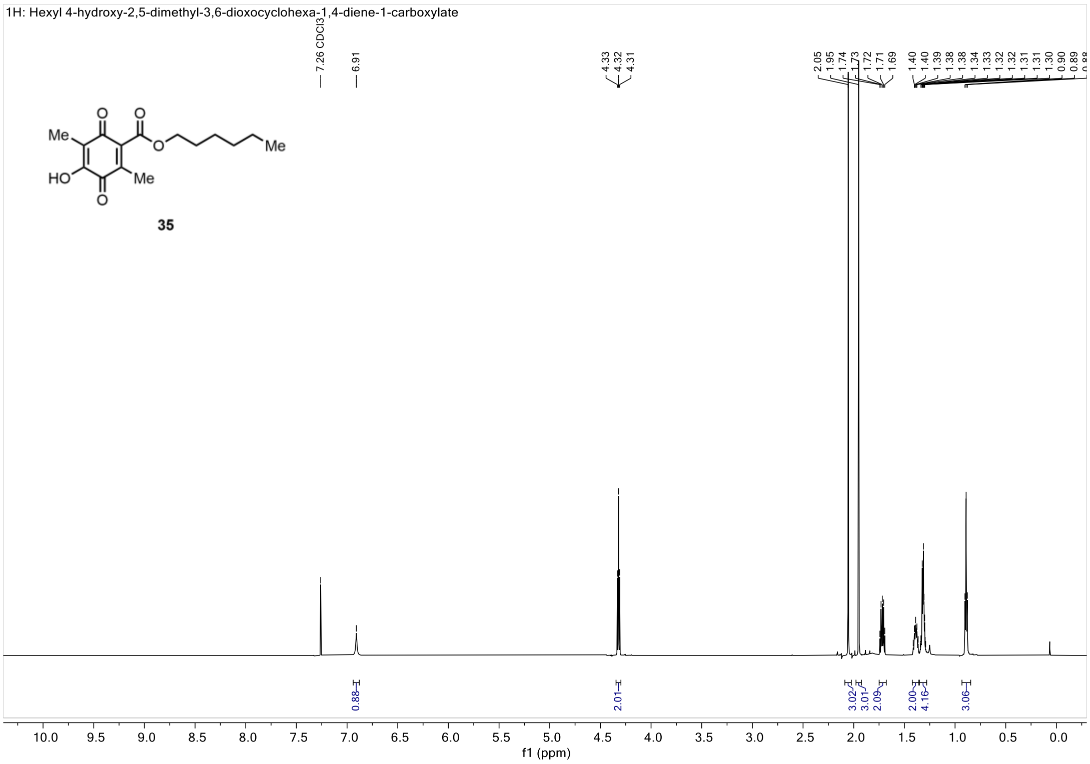

Fig. S54 ^{1}H-NMR spectrum of hexyl 4-hydroxy-2,5-dimethyl-3,6-dioxocyclohexa-1,4-diene-1-carboxylate (**35**), measured in $CDCl_3$ at 600 MHz.

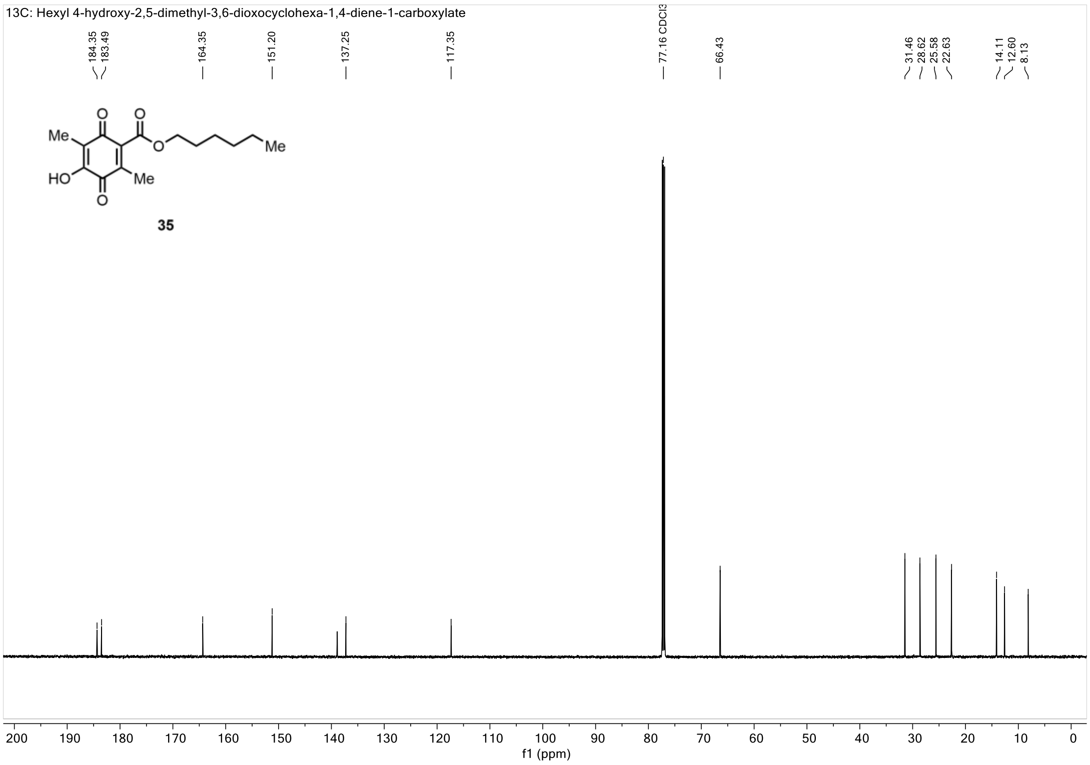

Fig. S55 ^{13}C-NMR spectrum of hexyl 4-hydroxy-2,5-dimethyl-3,6-dioxocyclohexa-1,4-diene-1-carboxylate (**35**), measured in $CDCl_3$ at 75 MHz.

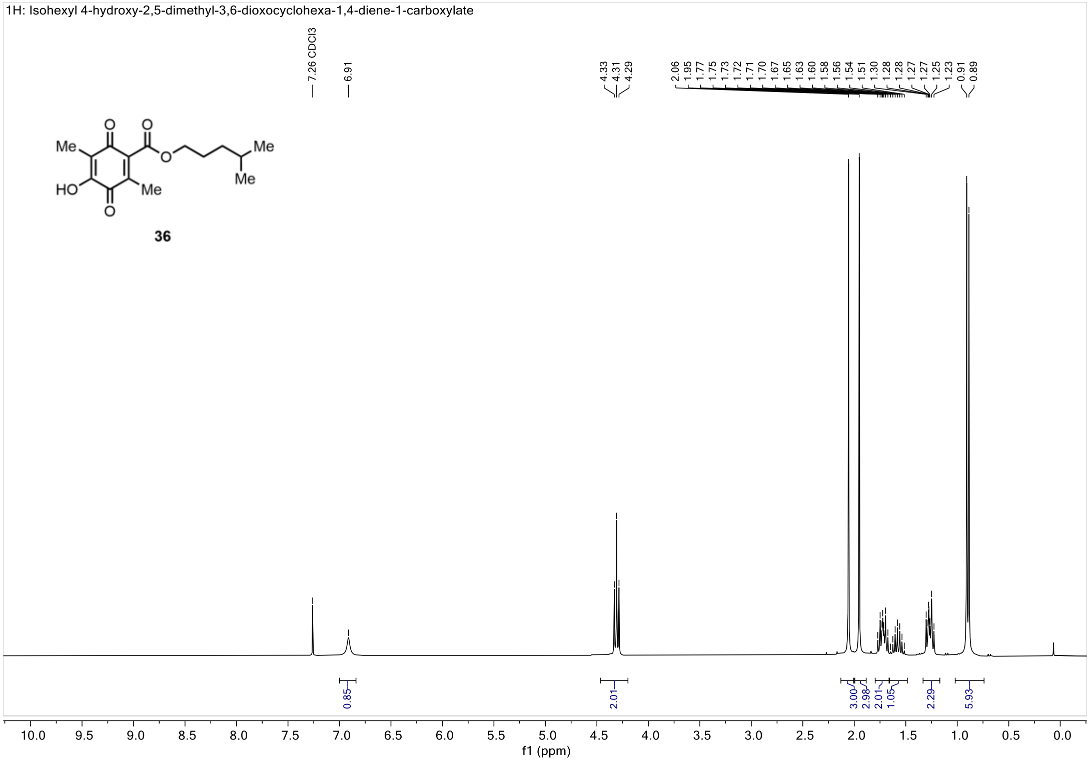

Fig. S56 ^{1}H-NMR spectrum of isohexyl 4-hydroxy-2,5-dimethyl-3,6-dioxocyclohexa-1,4-diene-1-carboxylate (**36**), measured in $CDCl_3$ at 300 MHz.

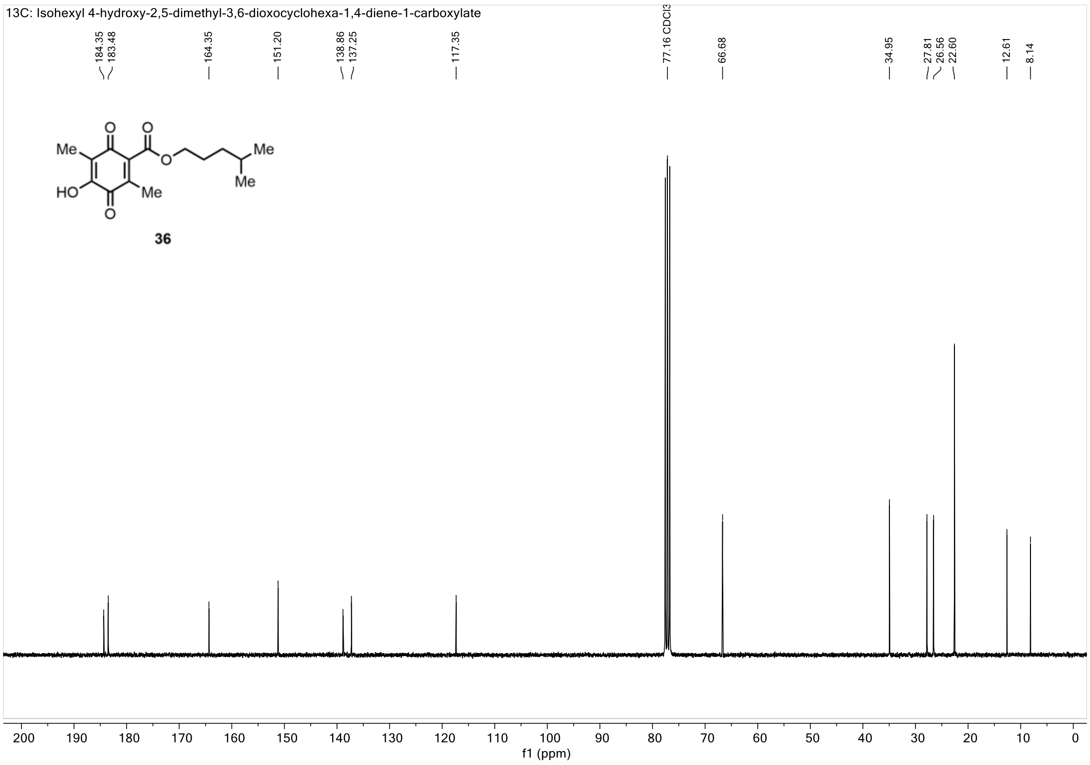

Fig. S57 ^{13}C-NMR spectrum of isohexyl 4-hydroxy-2,5-dimethyl-3,6-dioxocyclohexa-1,4-diene-1-carboxylate (**36**), measured in $CDCl_3$ at 75 MHz.

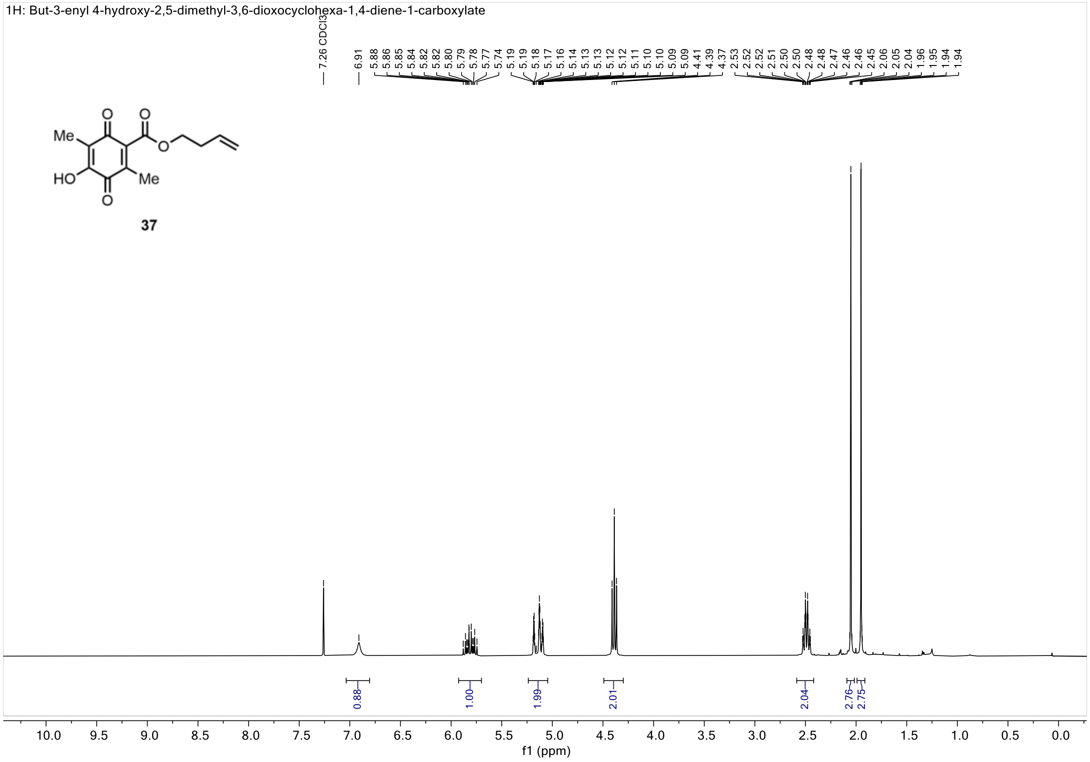

Fig. S58 ^{1}H-NMR spectrum of but-3-enyl 4-hydroxy-2,5-dimethyl-3,6-dioxocyclohexa-1,4-diene-1-carboxylate (**37**), measured in $CDCl_3$ at 300 MHz.

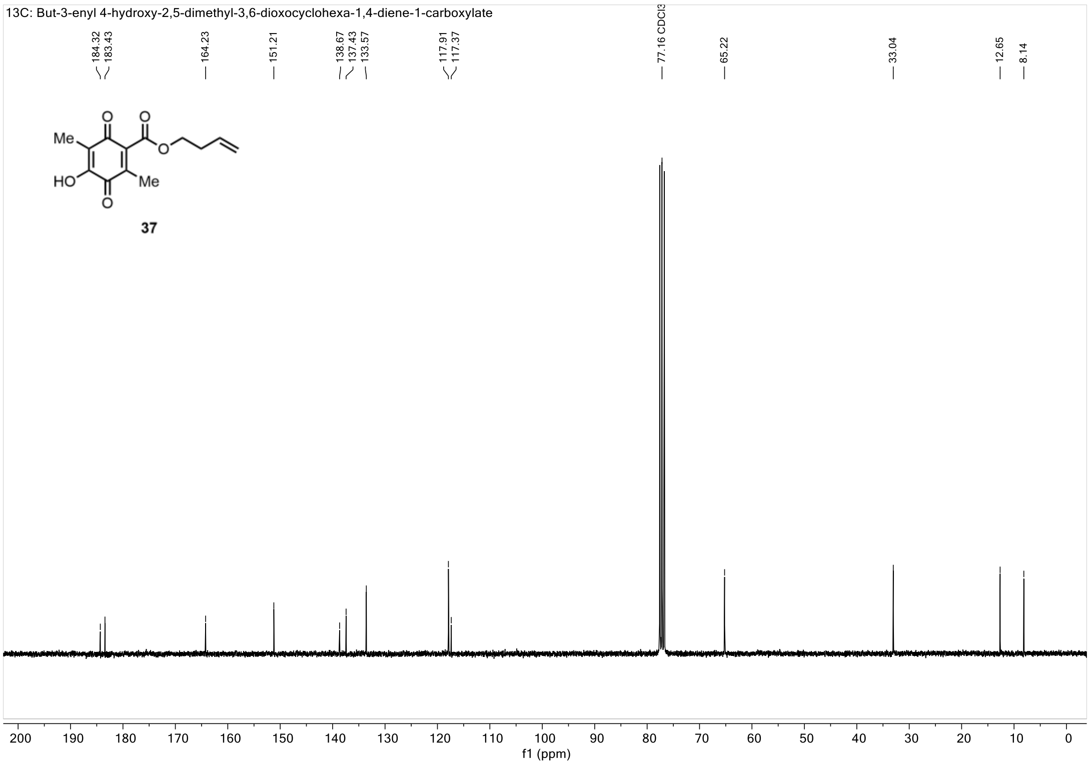

Fig. S59 ^{13}C-NMR spectrum of but-3-enyl 4-hydroxy-2,5-dimethyl-3,6-dioxocyclohexa-1,4-diene-1-carboxylate (**37**), measured in $CDCl_3$ at 75 MHz.

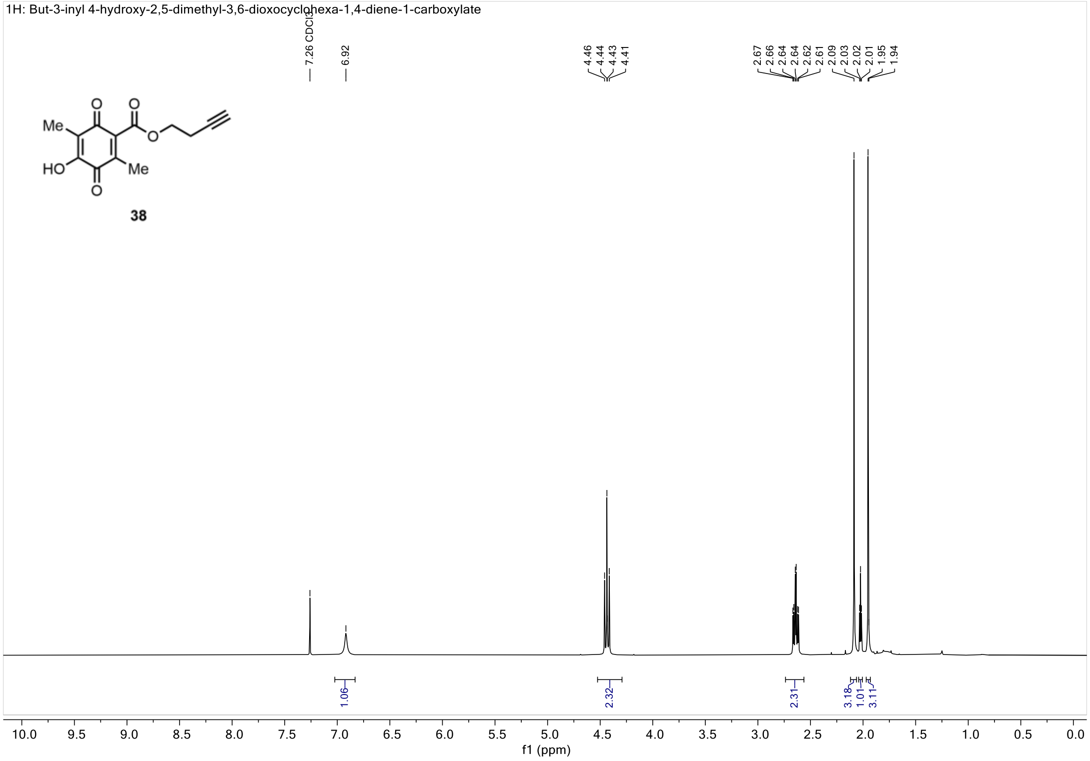

Fig. S60 ^{1}H-NMR spectrum of but-3-inyl 4-hydroxy-2,5-dimethyl-3,6-dioxocyclohexa-1,4-diene-1-carboxylate (**38**), measured in $CDCl_3$ at 300 MHz.

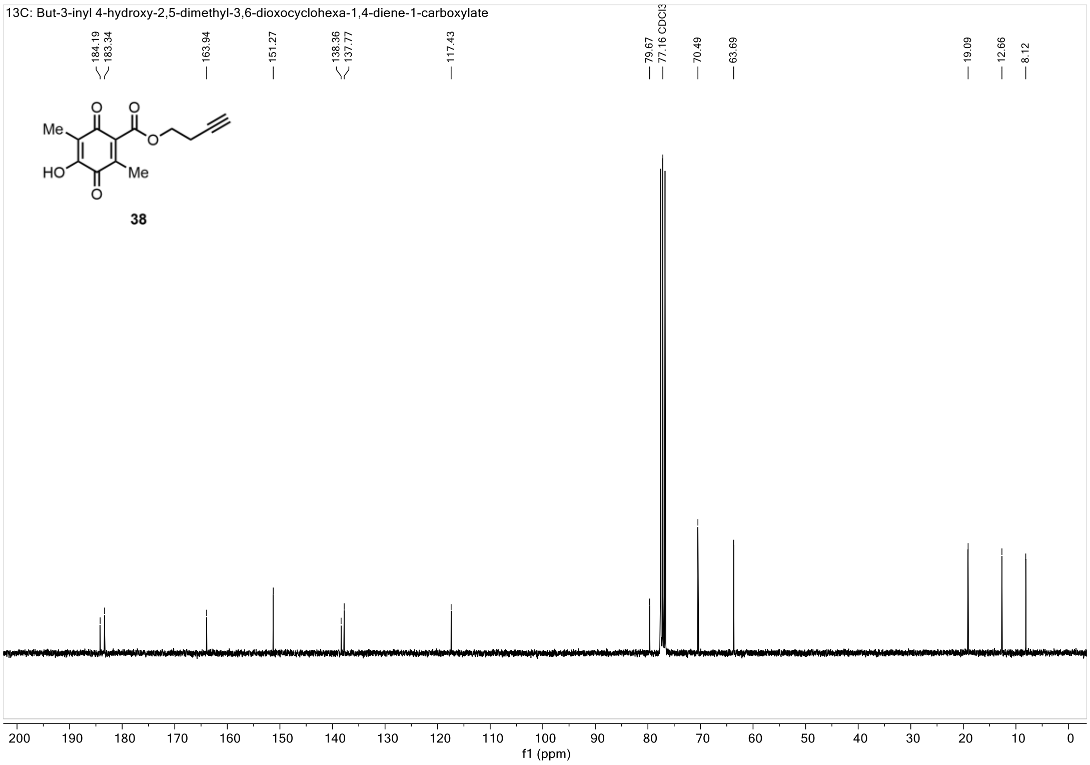

Fig. S61 ^{13}C-NMR spectrum of but-3-inyl 4-hydroxy-2,5-dimethyl-3,6-dioxocyclohexa-1,4-diene-1-carboxylate (**38**), measured in $CDCl_3$ at 75 MHz.

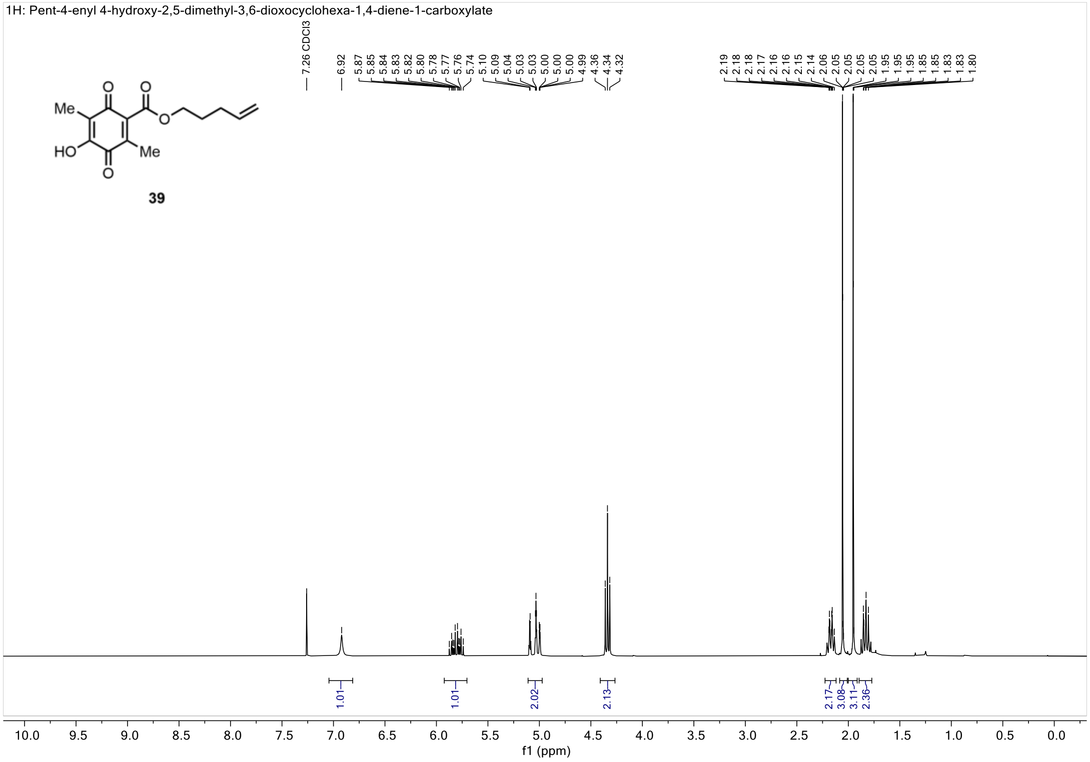

Fig. S62 ^{1}H-NMR spectrum of pent-4-enyl 4-hydroxy-2,5-dimethyl-3,6-dioxocyclohexa-1,4-diene-1-carboxylate (**39**), measured in $CDCl_3$ at 300 MHz.

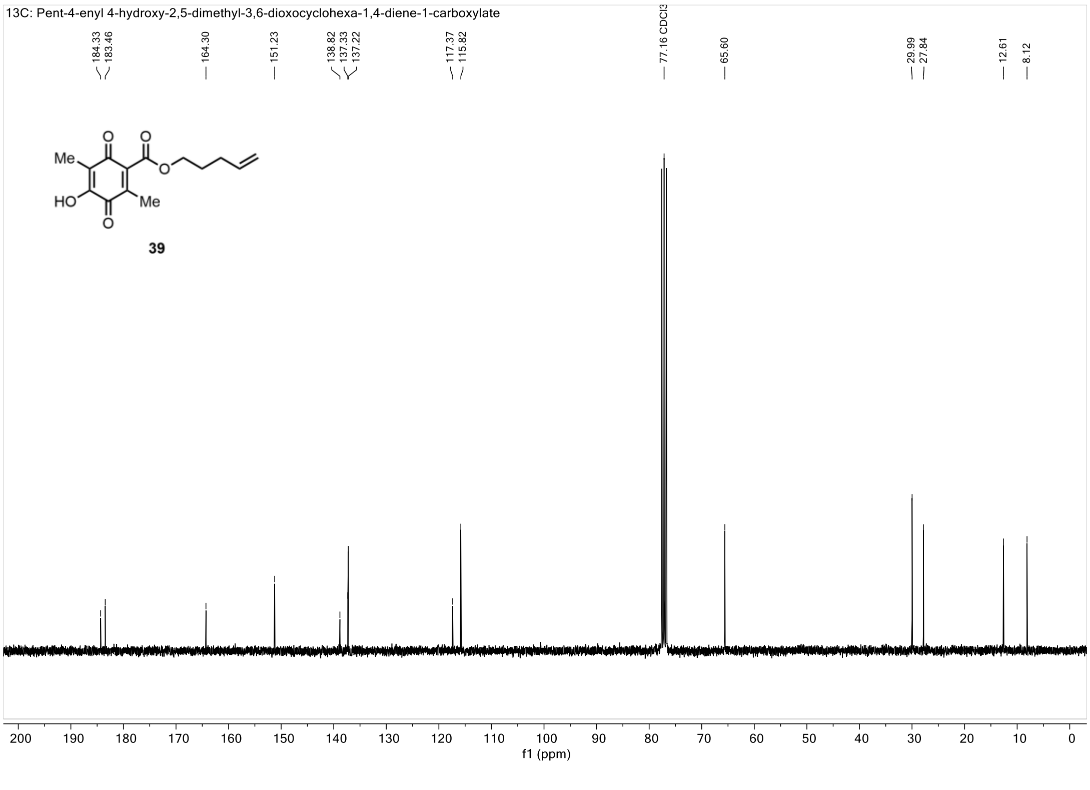

Fig. S63 ^{13}C-NMR spectrum of pent-4-enyl 4-hydroxy-2,5-dimethyl-3,6-dioxocyclohexa-1,4-diene-1-carboxylate (**39**), measured in $CDCl_3$ at 75 MHz.

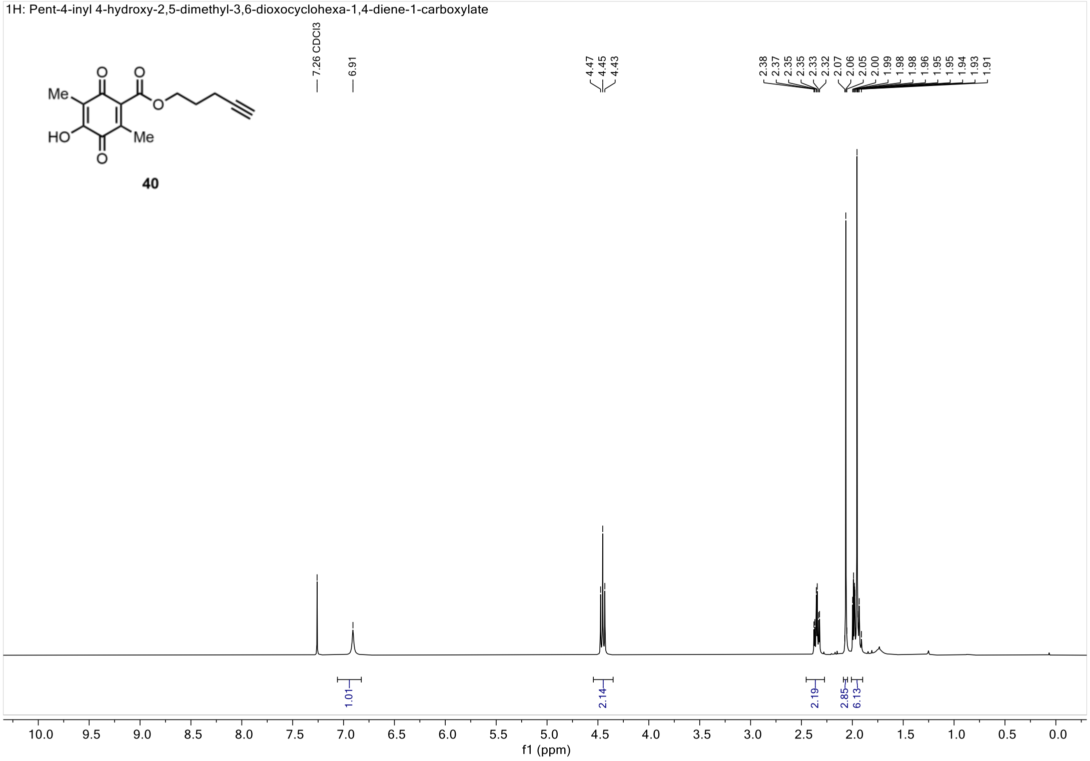

Fig. S64 ^{1}H-NMR spectrum of pent-4-inyl 4-hydroxy-2,5-dimethyl-3,6-dioxocyclohexa-1,4-diene-1-carboxylate (**40**), measured in $CDCl_3$ at 300 MHz.

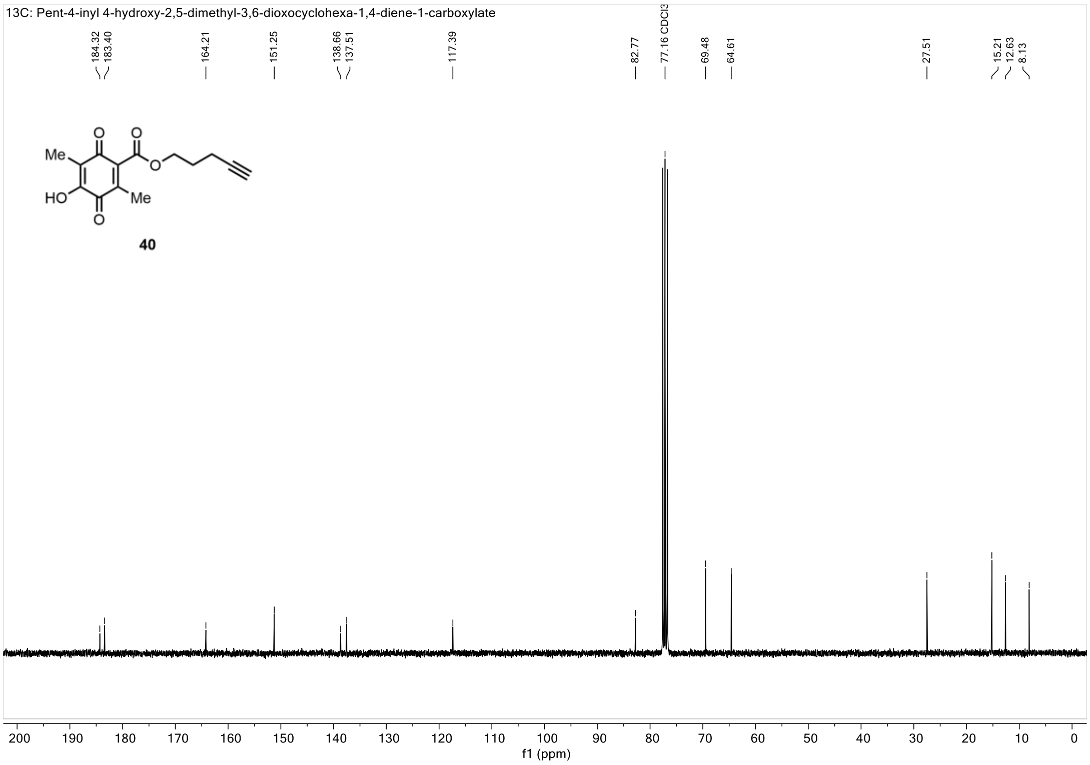

Fig. S65 ^{13}C-NMR spectrum of pent-4-inyl 4-hydroxy-2,5-dimethyl-3,6-dioxocyclohexa-1,4-diene-1-carboxylate (**40**), measured in $CDCl_3$ at 75 MHz.

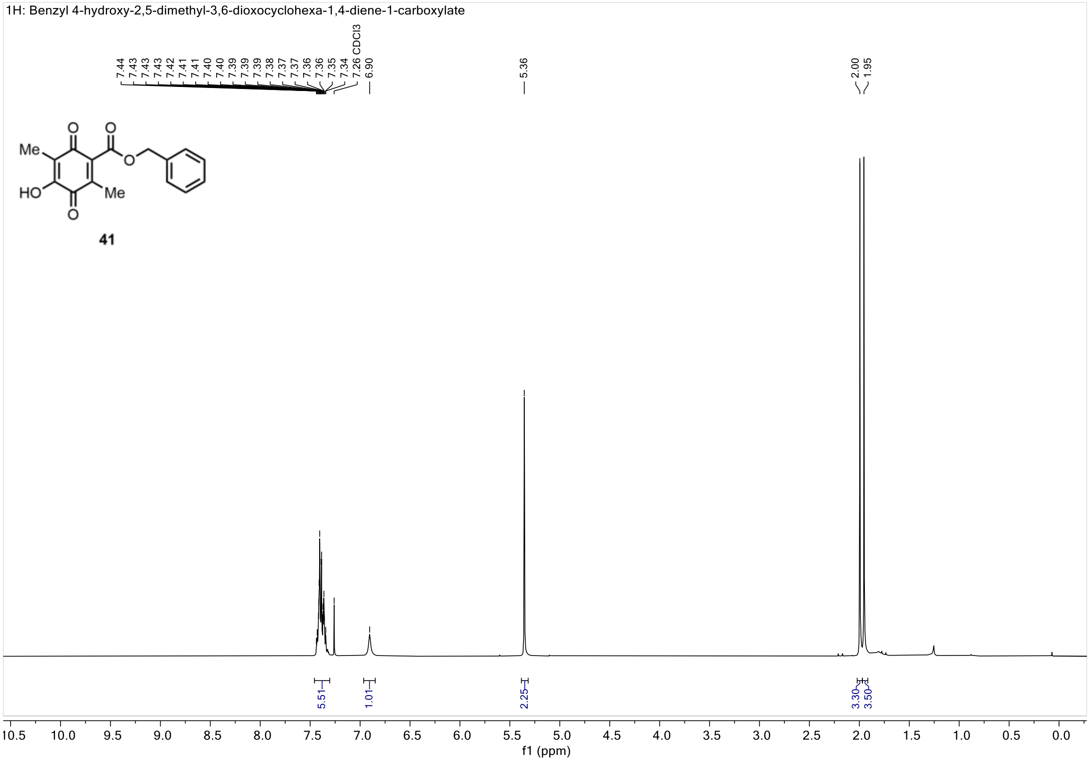

Fig. S66 ^{1}H-NMR spectrum of benzyl 4-hydroxy-2,5-dimethyl-3,6-dioxocyclohexa-1,4-diene-1-carboxylate (**41**), measured in $CDCl_3$ at 300 MHz.

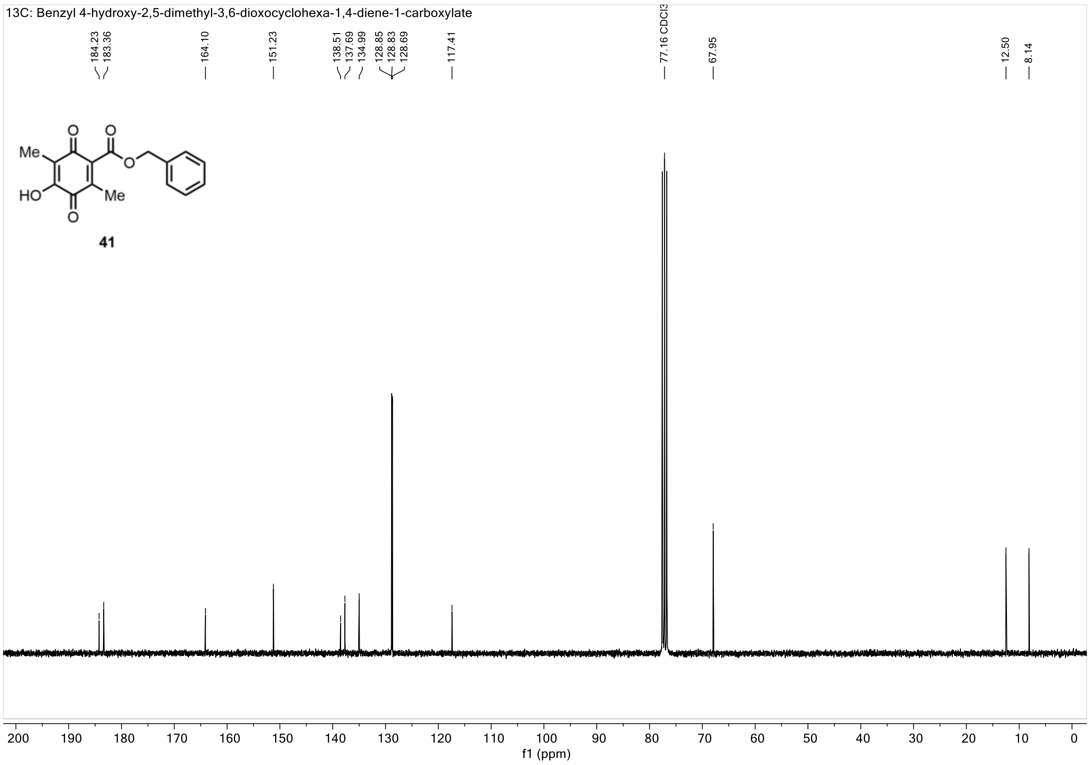

Fig. S67 ^{1}H-NMR spectrum of benzyl 4-hydroxy-2,5-dimethyl-3,6-dioxocyclohexa-1,4-diene-1-carboxylate (**41**), measured in $CDCl_3$ at 75 MHz.

5. Supplementary References

1. Milzarek, T. M. *et al.* Bypassing Biocatalytic Substrate Limitations in Oxidative Dearomatization Reactions by Transient Substrate Mimicking. *Org. Lett.* **21,** 4520–4524 (2019).

2. Tobias A. M. Gulder. Novel Bioactive Natural Products : Structural Elucidation, Biosynthesis and Synthesis as well as Stereochemical Analysis of Natural Products and Synthetic Compounds Using HPLC-CD. PhD Thesis, 2008.

3. Sib, A. & Gulder, T. A. M. Chemo-enzymatic Total Synthesis of Oxosorbicillinol, Sorrentanone, Rezishanones B and C, Sorbicatechol A, Bisvertinolone, and (+)-Epoxysorbicillinol. *Angew. Chem. Int. Ed.* **57,** 14650–14653 (2018).

4. Carpentier, C. *et al.* Dibenzofurans and Pseudodepsidones from the Lichen Stereocaulon paschale Collected in Northern Quebec. *J. Nat. Prod.* **80,** 210–214; 10.1021/acs.jnatprod.6b00831 (2017).

5. Satam, V., Harad, A., Rajule, R. & Pati, H. 2-Iodoxybenzoic acid (IBX): an efficient hypervalent iodine reagent. *Tetrahedron* **66,** 7659–7706; 10.1016/j.tet.2010.07.014 (2010).

6. van Dort, H. M. & Geursen, H. J. Salcomine-catalyzed oxidations of some phenols: A new method for the preparation of a number of para -benzoquinones. *Recl. Trav. Chim. Pays-Bas* **86,** 520–526; 10.1002/recl.19670860506 (1967).

7. Milzarek, T. M., Schuler, S., Matura, A. & Gulder, T. A. M. Evaluation of the Substrate Promiscuity of SorbC for the Chemo-Enzymatic Total Synthesis of Structurally Diverse Sorbicillinoids. *ACS Catal.* **12,** 1898–1904; 10.1021/acscatal.1c05196 (2022).

8. Brackman, G., Cos, P., Maes, L., Nelis, H. J. & Coenye, T. Quorum sensing inhibitors increase the susceptibility of bacterial biofilms to antibiotics in vitro and in vivo. *Antimicrobial Agents and Chemotherapy* **55,** 2655–2661; 10.1128/AAC.00045-11 (2011).

9. Andrejević, T. P. *et al.* Silver(i) complexes with different pyridine-4,5-dicarboxylate ligands as efficient agents for the control of cow mastitis associated pathogens. *Dalton Trans.* **49,** 6084–6096; 10.1039/D0DT00518E (2020).

10. Arthington-Skaggs, B. A., Jradi, H., Desai, T. & Morrison, C. J. Quantitation of ergosterol content: novel method for determination of fluconazole susceptibility of Candida albicans. *J. Clin. Microbiol.* **37,** 3332–3337; 10.1128/jcm.37.10.3332-3337.1999 (1999).
